# Global, regional, and national burden of head and neck cancer in males and associated risk factors from 1990 to 2021: a systematic analysis for the Global Burden of Disease Study 2021

**DOI:** 10.3389/fonc.2025.1607890

**Published:** 2025-10-31

**Authors:** Junjie Jiang, Zhongfang Xia, Wei Yao

**Affiliations:** Department of Otolaryngology, Wuhan Children’s Hospital, Tongji Medical College, Huazhong University of Science &Technology, Wuhan, China

**Keywords:** Global Burden of Disease (GBD), socio-demographic Index (SDI), head and neck cancer (HNC), male, risk factor

## Abstract

**Background:**

Head and neck cancer (HNC) is one of the most prevalent malignant tumors, with higher incidence and mortality rates in men than in women, particularly for lip and oral cavity, nasopharyngeal, laryngeal, and other pharyngeal cancers. This study investigates global trends in the occurrence of these cancers in men from 1990 to 2021 and analyzes their changing trends to guide healthcare policymakers in resource allocation.

**Methods:**

Using data from the 2021 Global Burden of Disease Study (GBD 2021), this study assesses the global prevalence, incidence, mortality, and disability-adjusted life years (DALYs) for male head and neck cancers. It also evaluates the relationship between cancer burden and economic development using the Socio-Demographic Index (SDI) and analyzes the risk factors for male head and neck cancer mortality and DALYs.

**Results:**

From 1990 to 2021, the impact of male head and neck cancers increased at varying rates. In 2021, there were 968,573 prevalent cases of lip and oral cavity cancer, 272,917 incident cases, 136,890 deaths, and 3,969,812 DALYs globally. The burden of nasopharyngeal, laryngeal, and other pharyngeal cancers was lower, with 385,913, 939,924, and 258,723 prevalent cases, respectively. The age-standardized incidence rates for all four cancers were positively correlated with the SDI. Key risk factors for male head and neck cancers include smoking and alcohol consumption. Additional risk factors include chewing tobacco for lip and oral cavity cancer deaths, formaldehyde exposure for nasopharyngeal cancer, and occupational exposure to sulfuric acid and asbestos for laryngeal cancer.

**Conclusions:**

Lip and oral cavity cancer remains the most burdensome, while nasopharyngeal cancer is increasing in East and Southeast Asia. Laryngeal cancer has declined in high-SDI regions, while other pharyngeal cancers are rising. Gender and lifestyle are key risk factors, underscoring the need for early prevention, particularly in resource-limited areas. As the global population ages, targeted prevention and improved healthcare infrastructure are essential.

## Background

Head and neck cancer (HNC) is the seventh most common cancer globally, with over 900,000 new cases reported in 2020 (1). Studies show that the incidence and mortality rates in men are significantly higher than in women (2), especially for cancers such as lip and oral cavity cancer, nasopharyngeal cancer, oropharyngeal cancer, and laryngeal cancer. According to the latest data from the World Health Organization (WHO) in 2022, lip and oral cavity cancer accounted for 2% of all new cancer cases, laryngeal cancer for 0.9%, nasopharyngeal cancer for 0.6%, oropharyngeal cancer and hypopharyngeal cancer for 0.5% and 0.4%, respectively (3). These cancers are not only closely related to gender differences but are also affected by various factors, including lifestyle habits and environmental influences (4). Therefore, head and neck cancer has emerged as a significant health issue for the male population.

Lip and oral cavity cancer, laryngeal cancer, nasopharyngeal cancer, oropharyngeal cancer, and hypopharyngeal cancer rank 16th, 20th, 23rd to 25th in global cancer incidence, indicating their significant position in the global cancer burden (3). Significant regional variations exist in the incidence and mortality rates of head and neck cancer worldwide. According to the Global Burden of Disease (GBD) 2019 data, high Socio-Demographic Index (SDI) regions have the greatest age-standardized incidence rate (ASIR) for HNC, but the lowest age-standardized death rate (ASDR). This phenomenon is closely related to regional lifestyles, healthcare resources, and public health policies, reflecting the gap between affluent and economically disadvantaged nations (5). In some countries, high-risk factors such as smoking, alcohol consumption, and HPV infection are more prevalent, leading to a higher incidence of HNC. In contrast, in other regions, despite similar risk factors, the mortality rates are better controlled due to improvements in public health policies and early screening.

Therefore, analyzing global and regional HNC data is crucial for identifying differences in disease control and prevention strategies, particularly in interventions for men and high-risk populations. To lay a scientific foundation for policy development, it is crucial to better understand the burden and associated risk factors of these cancers in the male population across various regions. Based on data from the 2021 GBD, this study aims to analyze the burden of male HNC, including lip and oral cavity cancer, nasopharyngeal cancer, laryngeal cancer, and other pharyngeal cancers, and its associated risk factors from 1990 to 2021 in 204 countries and regions, classified by gender and SDI. The study aims to provide robust data support to policymakers, assisting them in designing effective risk control strategies for high-risk male populations.

## Methods

### Data acquisition

GBD 2021 provides epidemiological data on 371 diseases and injuries globally (6, 7).

The methods for gathering and analysis are detailed in several published studies. The project follows the Guidelines for Accurate and Transparent Health Estimates Reporting (GATHER) guidelines to maintain the transparency and correctness of health evaluation reports. This study is based on data from the GBD 2021 database, covering relevant statistical indicators for lip and oral cancer, nasopharyngeal cancer, laryngeal cancer, and other pharyngeal cancers from 1990 to 2021. These indicators include the number of prevalent cases, incidence, mortality, disability-adjusted life years (DALYs), and corresponding age-standardized rates (ASRs). All data were extracted using the GBD visualization platform (http://ghdx.healthdata.org/GBD-results-tool).

The range of SDI is from 0 to 1, and it is used to assess the relationship between socio-economic status and health levels (8, 9). Based on the SDI values, countries and regions are classified into five tiers: low, lower-middle, middle, upper-middle, and high, to analyze the link between cancer burden and socio-economic development. By analyzing SDI rankings and scores, the differences in socio-economic status, education levels, and fertility rates across countries are revealed, providing a basis for identifying potential areas for health improvement.

The GBD 2021 database offers an extensive evaluation of how exposure to risk factors influences specific health outcomes (7). The study assesses 88 risk factors, Split into four levels. This study provides a summary of the Level 4 risk factors related to male head and neck cancers as follows: (1) Lip and oral cavity cancer: smoking, high alcohol use, chewing tobacco; (2) Nasopharyngeal cancer: smoking, high alcohol use, occupational exposure to formaldehyde; (3) Laryngeal cancer: smoking, high alcohol use, occupational exposure to sulfuric acid and asbestos; (4) Other pharyngeal cancers: smoking and high alcohol use.

### Statistical analysis

By applying the Estimated Annual Percentage Change (EAPC), this study analyzed the global trends in age-standardized rates, including the age-standardized prevalence rate (ASPR), incidence rate (ASIR), mortality rate (ASDR), and disability-adjusted life years (ASDALYR) for four types of cancer. The age-standardized rate (ASR) per 100,000 population was calculated using the following mathematical formula:


$$ASR=\frac{\sum_{i=1}^{A}a_{i}w_{i}}{\sum_{i=1}^{A}w_{i}}\times 100,000$$

(a_i_: The ASR (age-standardized rate) for the ith age group; w: The population size of the ith age group in the standard population; A: The total number of age groups).

The EAPC is estimated using a regression model that evaluates trends in age-standardized rates (ASR) over time (10). The regression formula is expressed as: Y=α+βX+e , where Y is the natural logarithm of the ASR, X represents the year, α is the intercept, β denotes the trend (slope), and e is the error term. The EAPC is calculated using the equation: EAPC = 100 × [exp( β ) - 1]. This value represents the annual percentage change in ASR. This value represents the annual percentage change in ASR. Positive EAPC values with 95% confidence intervals (CIs) above zero indicate an increasing trend, while negative values signify a decreasing trend. For all the indicators in this study, the uncertainty intervals (UIs) are reported as 95% uncertainty intervals (UI) derived from 1,000 posterior draws. These intervals provide a measure of variability in the data, accounting for the uncertainty in the model estimates. For the EAPC, 95% confidence intervals (CIs) are used to report the trend estimates.

This study conducted Spearman’s correlation analysis to explore the relationship between SDI and age-standardized incidence rates of laryngeal cancer. Data for this study was obtained from publicly accessible databases, obviating the need for clinical ethical review. All the statistical analyses were performed using R software (version 4.4.0).

## Result

### Lip and oral cavity cancer

**Figure 1:**
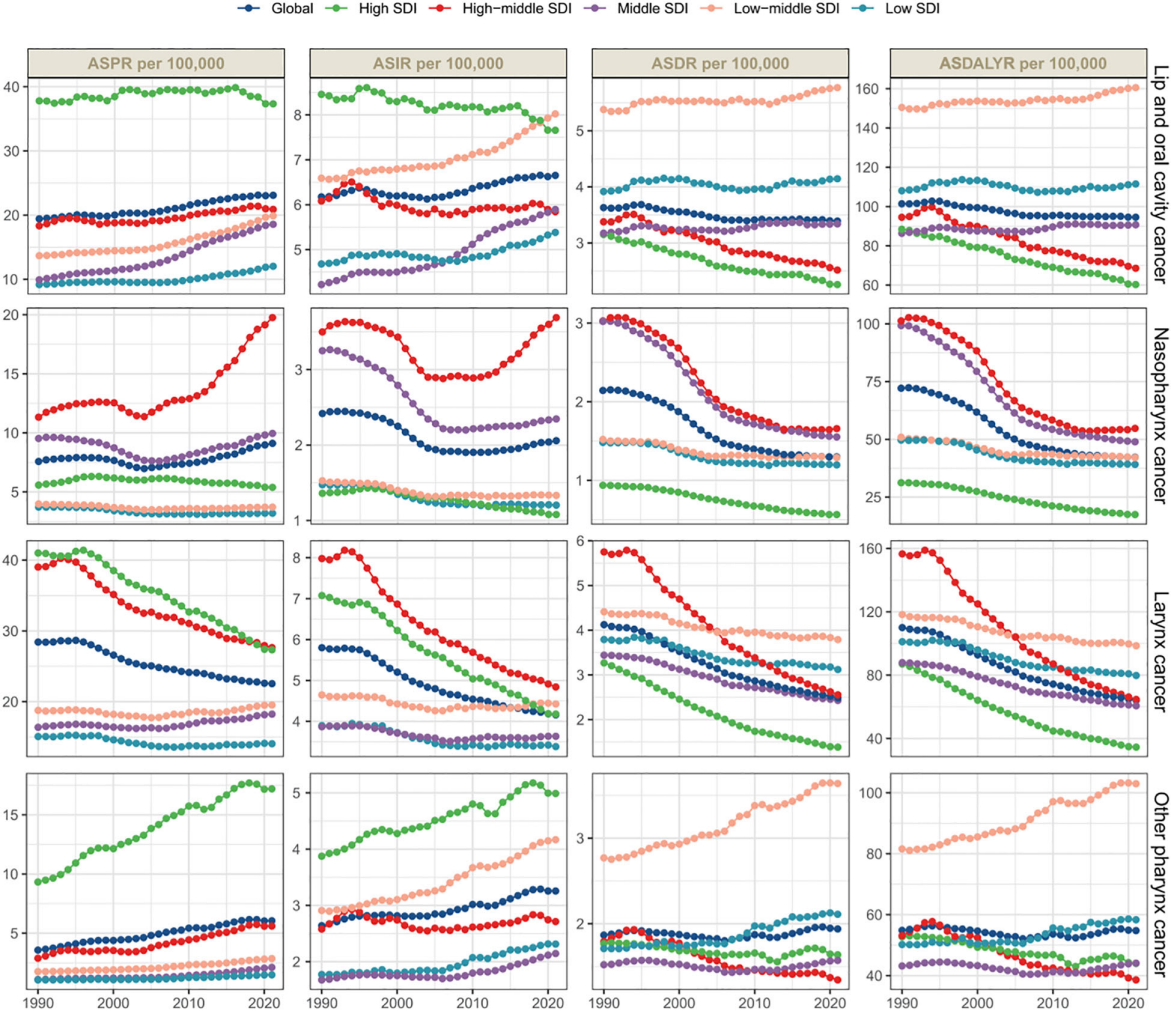
Global and regional trends in male head and neck cancer incidences, deaths, and disability-adjusted life years (DALYs) display the age-standardized prevalence rates (ASPRs), age-standardized incidence rates (ASIRs), age-standardized death rates (ASDRs), and age-standardized DALY rates (ASDALYR), highlighting the varying trends across SDI regions.

**Figure 2:**
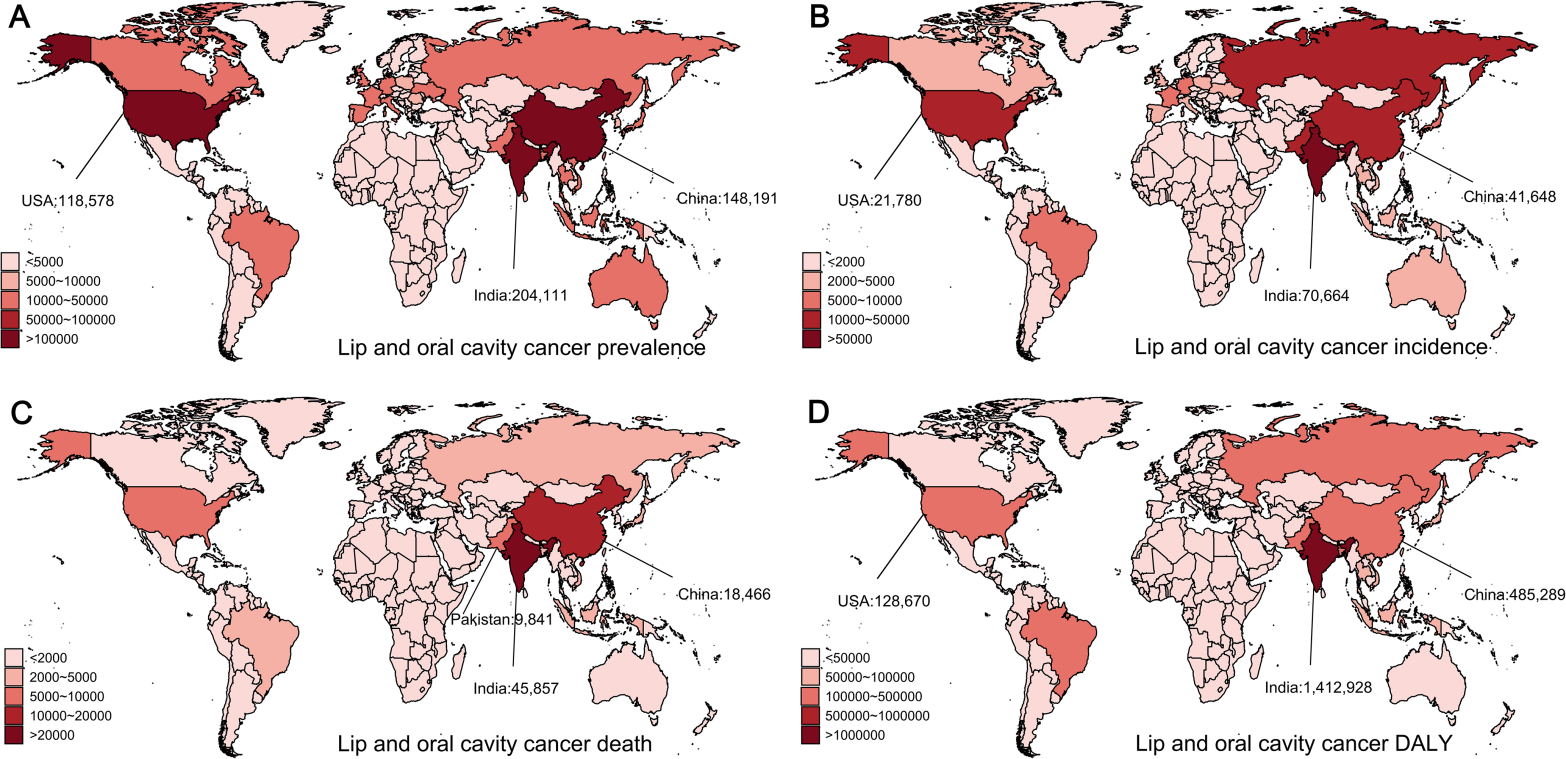
Global distribution of lip and oral cavity cancer prevalences, incidences, deaths, and disability-adjusted life years (DALYs) in 2021. **(A)** Prevalence of lip and oral cavity cancer. **(B)** Incidence of lip and oral cavity cancer. **(C)** Mortality from lip and oral cavity cancer. **(D)** DALYs from lip and oral cavity cancer.

In the last three decades, the global age-standardized prevalence rate (ASPR) of lip and oral cavity cancer has shown an increasing trend (**Figure 1**). Specifically, during the years 1990 to 2021, the number of global cases of lip and oral cavity cancer increased from 393,846 to 968,573, with the ASPR rising from 19.42 to 23.09 (EAPC = 0.62, 95% CI: [0.56, 0.67]). Areas with high SDI recorded the greatest prevalence rates and ASPR (**Figure 1**). In 2021, South Asia reported the greatest number of cases, with 260,373 cases (95% UI: 206,423–305,436) (**Table 1**). Australasia had the greatest ASPR, reaching 55.78 (95% UI: 47.84–64.34), nearly 2.4 times the global average. At the country level, India had the greatest number of prevalence cases (204,111, 95% UI: 160,011–242,292), followed by China (148,191, 95% UI: 110,241–194,522) and the United States (118,578, 95% UI: 112,447–123,612) (**Figure 2A**), with around 50% of the worldwide new cases attributed to these three countries. Globally, Taiwan, China had the greatest ASPR (**Supplementary Figure S1A**).

**Table 1:** Global and regional trends in lip and oral cavity cancer burden: Prevalence, incidence, mortality, and disability-adjusted life years (1990-2021).

Location	1990 prevalence cases (95% UI)	1990 ASPR (95% UI)	2021 prevalence cases (95% UI)	2021 ASPR (95% UI)	1990–2021 EAPC (95% CI)
Global	393846 (379155-408551)	19.42 (18.71-20.15)	968573 (879564-1049185)	23.09 (20.99-24.97)	0.62 (0.56 to 0.67)
High SDI	183036 (177657-188375)	37.8 (36.68-38.89)	326168 (312706-339380)	37.34 (35.82-38.77)	0.08 (0.01 to 0.16)
High-middle SDI	87635 (84123-91342)	18.34 (17.59-19.07)	191189 (171647-213834)	20.9 (18.83-23.31)	0.43 (0.34 to 0.52)
Middle SDI	59802 (55137-64876)	9.93 (9.17-10.76)	256142 (218361-292906)	18.57 (15.88-21.22)	2.14 (2 to 2.28)
Low-middle SDI	50319 (43099-57511)	13.69 (11.75-15.63)	157425 (128461-181418)	19.9 (16.41-22.87)	1.2 (1.08 to 1.33)
Low SDI	12617 (10279-14752)	9.18 (7.51-10.76)	36790 (28828-44268)	12.03 (9.52-14.46)	0.74 (0.6 to 0.88)
High-income Asia Pacific	12844 (12295-13462)	13.58 (12.98-14.21)	36234 (32968-39523)	21.23 (19.37-23.06)	1.52 (1.21 to 1.83)
High-income North America	79816 (77452-81891)	53.69 (52.14-55.11)	129298 (123344-134768)	44.99 (42.92-46.86)	-0.47 (-0.56 to -0.39)
Western Europe	112222 (107084-117705)	47.61 (45.44-49.87)	149335 (138322-159355)	41.04 (38.16-43.69)	-0.46 (-0.55 to -0.37)
Australasia	5872 (5198-6577)	53.9 (47.67-60.39)	12754 (10966-14724)	55.78 (47.84-64.34)	0.13 (-0.01 to 0.27)
Andean Latin America	362 (309-421)	3.22 (2.73-3.77)	1377 (1074-1738)	4.63 (3.61-5.85)	1.47 (1.28 to 1.67)
Tropical Latin America	7204 (6818-7563)	14.44 (13.62-15.19)	22534 (21011-24319)	18.24 (17.04-19.69)	0.68 (0.58 to 0.78)
Central Latin America	2291 (2192-2392)	5.11 (4.89-5.33)	7601 (6616-8630)	6.36 (5.54-7.2)	0.47 (0.36 to 0.57)
Southern Latin America	3186 (2862-3534)	14.55 (13.07-16.13)	5143 (4487-5872)	13.36 (11.67-15.23)	-0.09 (-0.36 to 0.18)
Caribbean	2149 (1984-2328)	16.48 (15.19-17.86)	4996 (4191-5847)	19.44 (16.35-22.68)	0.84 (0.69 to 0.98)
Central Europe	14913 (14116-15743)	21.77 (20.61-22.98)	30292 (27351-33137)	33.45 (30.15-36.63)	1.29 (1.11 to 1.47)
Eastern Europe	22885 (21681-24987)	19.84 (18.78-21.59)	37248 (32467-42807)	27.21 (23.77-31.25)	0.83 (0.63 to 1.03)
Central Asia	2313 (2151-2509)	10.49 (9.73-11.41)	3682 (3211-4189)	9 (7.87-10.18)	-0.38 (-0.72 to -0.03)
North Africa and Middle East	3093 (2641-3640)	3.08 (2.61-3.58)	13238 (11342-15160)	4.95 (4.27-5.64)	1.53 (1.46 to 1.6)
South Asia	75862 (66380-85549)	20.66 (18.04-23.32)	260373 (206423-305436)	31.48 (25.06-36.83)	1.32 (1.18 to 1.45)
Southeast Asia	14271 (12193-16275)	10.08 (8.64-11.46)	56457 (46904-66522)	16.31 (13.64-19.11)	1.5 (1.42 to 1.58)
East Asia	27392 (23012-32268)	5.51 (4.63-6.48)	178397 (141152-224934)	16.45 (13.08-20.65)	4.05 (3.86 to 4.24)
Oceania	102 (71-132)	5.17 (3.64-6.61)	333 (241-429)	6.65 (4.84-8.51)	0.94 (0.84 to 1.05)
Western Sub-Saharan Africa	915 (717-1114)	1.71 (1.35-2.08)	2872 (2141-3617)	2.4 (1.83-2.99)	1.05 (0.9 to 1.21)
Eastern Sub-Saharan Africa	3468 (2914-3992)	7.8 (6.53-8.99)	9954 (7487-12231)	9.65 (7.43-11.76)	0.61 (0.47 to 0.74)
Central Sub-Saharan Africa	575 (424-856)	4.56 (3.36-6.68)	1912 (1428-2532)	5.87 (4.48-7.61)	0.81 (0.55 to 1.07)
Southern Sub-Saharan Africa	2114 (1566-2602)	15.12 (10.95-18.7)	4541 (3819-5155)	15.38 (13.09-17.28)	-0.24 (-0.35 to -0.14)
	1990 incidence cases (95% UI)	1990 ASIR (95% UI)	2021 incidence cases (95% UI)	2021 ASIR (95% UI)	1990–2021 EAPC (95% CI)
Global	118387 (112777-124445)	6.17 (5.88-6.49)	272917 (245321-296016)	6.65 (5.99-7.21)	0.23 (0.16 to 0.29)
High SDI	40239 (38981-41402)	8.46 (8.18-8.7)	68826 (65517-71809)	7.66 (7.31-7.98)	-0.25 (-0.31 to -0.2)
High-middle SDI	27746 (26682-28937)	6.08 (5.83-6.34)	52866 (47658-58782)	5.84 (5.27-6.48)	-0.21 (-0.3 to -0.11)
Middle SDI	22621 (20849-24615)	4.22 (3.9-4.59)	77613 (66548-87891)	5.89 (5.08-6.66)	1.12 (1.02 to 1.22)
Low-middle SDI	21899 (18790-25090)	6.59 (5.62-7.54)	58881 (48622-67462)	8.02 (6.68-9.13)	0.58 (0.51 to 0.65)
Low SDI	5745 (4717-6786)	4.68 (3.86-5.56)	14496 (11582-17287)	5.38 (4.31-6.38)	0.29 (0.2 to 0.38)
High-income Asia Pacific	3742 (3584-3897)	4.19 (4-4.36)	10960 (9948-11869)	5.7 (5.2-6.16)	0.86 (0.54 to 1.18)
High-income North America	15611 (15138-16027)	10.56 (10.22-10.84)	23945 (22612-25017)	8.23 (7.78-8.58)	-0.74 (-0.85 to -0.63)
Western Europe	24624 (23574-25815)	10.45 (10-10.95)	30121 (27819-32159)	7.95 (7.38-8.48)	-0.88 (-0.94 to -0.82)
Australasia	1162 (1026-1308)	10.83 (9.61-12.19)	2275 (1965-2614)	9.69 (8.39-11.1)	-0.38 (-0.56 to -0.2)
Andean Latin America	150 (128-177)	1.47 (1.24-1.73)	443 (348-559)	1.55 (1.23-1.96)	0.35 (0.17 to 0.53)
Tropical Latin America	2619 (2474-2759)	5.71 (5.38-6.03)	7037 (6543-7598)	5.86 (5.43-6.32)	0.04 (-0.07 to 0.15)
Central Latin America	884 (847-922)	2.19 (2.1-2.29)	2444 (2139-2752)	2.12 (1.86-2.39)	-0.39 (-0.51 to -0.28)
Southern Latin America	1029 (920-1149)	4.85 (4.35-5.4)	1416 (1235-1613)	3.69 (3.23-4.2)	-0.67 (-0.91 to -0.42)
Caribbean	745 (686-810)	5.93 (5.46-6.43)	1513 (1293-1748)	5.95 (5.09-6.88)	0.25 (0.11 to 0.38)
Central Europe	4964 (4724-5252)	7.48 (7.12-7.9)	8349 (7598-9112)	9.15 (8.34-9.99)	0.54 (0.41 to 0.67)
Eastern Europe	10284 (9843-11040)	9.26 (8.85-9.9)	15199 (13344-17069)	11.01 (9.69-12.36)	0.25 (0.06 to 0.43)
Central Asia	862 (801-939)	4.22 (3.93-4.61)	1255 (1091-1449)	3.32 (2.91-3.79)	-0.68 (-0.93 to -0.43)
North Africa and Middle East	1101 (929-1291)	1.25 (1.04-1.45)	3592 (3103-4116)	1.49 (1.29-1.69)	0.52 (0.48 to 0.56)
South Asia	31824 (27674-36044)	9.68 (8.35-11)	92427 (73790-107478)	11.98 (9.63-13.89)	0.58 (0.49 to 0.66)
Southeast Asia	5216 (4461-5961)	4.17 (3.58-4.74)	16522 (13977-19048)	5.2 (4.44-5.99)	0.65 (0.62 to 0.69)
East Asia	10430 (8674-12391)	2.41 (2.01-2.84)	47857 (37470-60189)	4.58 (3.62-5.71)	2.5 (2.31 to 2.69)
Oceania	38 (27-50)	2.26 (1.59-2.86)	118 (84-154)	2.7 (1.94-3.46)	0.76 (0.66 to 0.86)
Western Sub-Saharan Africa	412 (326-502)	0.85 (0.68-1.04)	1139 (868-1422)	1.07 (0.84-1.31)	0.69 (0.6 to 0.78)
Eastern Sub-Saharan Africa	1613 (1355-1865)	4.08 (3.43-4.73)	3887 (2988-4749)	4.4 (3.47-5.3)	0.13 (0.07 to 0.19)
Central Sub-Saharan Africa	271 (199-399)	2.46 (1.87-3.48)	761 (572-993)	2.79 (2.13-3.58)	0.37 (0.2 to 0.55)
Southern Sub-Saharan Africa	807 (587-996)	6.31 (4.53-7.83)	1658 (1411-1863)	6.15 (5.33-6.87)	-0.35 (-0.51 to -0.18)
	1990 death cases (95% UI)	1990 ASDR (95% UI)	2021 death cases (95% UI)	2021 ASDR (95% UI)	1990–2021 EAPC (95% CI)
Global	66990 (62782-71608)	3.63 (3.4-3.89)	136890 (120656-149372)	3.39 (3-3.69)	-0.28 (-0.32 to -0.23)
High SDI	14658 (14191-15052)	3.15 (3.04-3.24)	20903 (19819-21890)	2.26 (2.15-2.36)	-1.06 (-1.11 to -1)
High-middle SDI	14714 (14079-15385)	3.38 (3.23-3.53)	22444 (20143-24822)	2.52 (2.26-2.78)	-1.07 (-1.14 to -1.01)
Middle SDI	15936 (14660-17390)	3.17 (2.92-3.46)	42459 (36636-47416)	3.34 (2.9-3.72)	0.14 (0.09 to 0.18)
Low-middle SDI	17035 (14537-19576)	5.38 (4.57-6.17)	40503 (33645-46285)	5.77 (4.83-6.56)	0.16 (0.12 to 0.21)
Low SDI	4563 (3779-5393)	3.92 (3.25-4.65)	10458 (8400-12440)	4.14 (3.35-4.89)	0.04 (-0.03 to 0.11)
High-income Asia Pacific	1285 (1241-1327)	1.5 (1.44-1.55)	3247 (2972-3434)	1.56 (1.44-1.65)	-0.23 (-0.57 to 0.12)
High-income North America	4423 (4283-4543)	3.03 (2.93-3.12)	5844 (5475-6103)	1.97 (1.84-2.05)	-1.33 (-1.51 to -1.14)
Western Europe	9571 (9164-9941)	4.08 (3.9-4.24)	9230 (8526-9804)	2.3 (2.14-2.44)	-1.87 (-1.96 to -1.78)
Australasia	285 (258-313)	2.74 (2.49-3.01)	469 (409-530)	1.89 (1.65-2.13)	-1.18 (-1.44 to -0.91)
Andean Latin America	114 (96-134)	1.16 (0.99-1.37)	267 (212-334)	0.96 (0.77-1.2)	-0.44 (-0.6 to -0.29)
Tropical Latin America	1772 (1675-1867)	4.06 (3.82-4.29)	4144 (3859-4453)	3.52 (3.27-3.79)	-0.43 (-0.55 to -0.32)
Central Latin America	629 (602-653)	1.64 (1.57-1.71)	1470 (1286-1657)	1.31 (1.15-1.47)	-0.99 (-1.1 to -0.87)
Southern Latin America	591 (529-662)	2.86 (2.57-3.19)	692 (609-783)	1.81 (1.6-2.05)	-1.16 (-1.39 to -0.93)
Caribbean	473 (437-518)	3.87 (3.58-4.23)	866 (737-1007)	3.43 (2.92-3.98)	-0.15 (-0.28 to -0.01)
Central Europe	3277 (3119-3450)	5.06 (4.82-5.32)	4515 (4145-4895)	4.94 (4.53-5.35)	-0.19 (-0.28 to -0.09)
Eastern Europe	5650 (5406-6018)	5.33 (5.09-5.67)	6670 (5873-7521)	4.85 (4.28-5.46)	-0.71 (-0.9 to -0.52)
Central Asia	626 (583-684)	3.18 (2.95-3.47)	827 (723-953)	2.28 (2.01-2.61)	-1.02 (-1.21 to -0.84)
North Africa and Middle East	749 (626-882)	0.91 (0.76-1.07)	1855 (1612-2124)	0.83 (0.72-0.94)	-0.4 (-0.45 to -0.34)
South Asia	24272 (21049-27547)	7.78 (6.68-8.9)	60952 (49383-70778)	8.26 (6.69-9.54)	0.06 (0 to 0.13)
Southeast Asia	3703 (3167-4226)	3.19 (2.74-3.63)	9632 (8244-11110)	3.25 (2.8-3.72)	0.01 (-0.01 to 0.04)
East Asia	7136 (5908-8496)	1.79 (1.5-2.11)	20771 (16202-26058)	2.07 (1.63-2.58)	0.73 (0.59 to 0.88)
Oceania	27 (19-36)	1.77 (1.24-2.26)	83 (58-108)	2.04 (1.45-2.65)	0.67 (0.57 to 0.77)
Western Sub-Saharan Africa	325 (258-397)	0.71 (0.57-0.86)	828 (643-1034)	0.83 (0.66-1.01)	0.46 (0.39 to 0.53)
Eastern Sub-Saharan Africa	1302 (1103-1522)	3.47 (2.93-4.02)	2870 (2234-3501)	3.5 (2.79-4.17)	-0.07 (-0.1 to -0.04)
Central Sub-Saharan Africa	221 (162-322)	2.13 (1.63-2.95)	572 (429-749)	2.28 (1.76-2.93)	0.21 (0.09 to 0.33)
Southern Sub-Saharan Africa	561 (406-695)	4.62 (3.32-5.74)	1087 (930-1218)	4.26 (3.71-4.75)	-0.53 (-0.78 to -0.28)
	1990 DALY cases (95% UI)	1990 ASDALYR (95% UI)	2021 DALY cases (95% UI)	2021ASDALYR (95% UI)	1990–2021 EAPC (95% CI)
Global	2065244 (1932213-2212857)	101.41 (94.96-108.49)	3969812 (3446429-4348773)	94.55 (82.23-103.44)	-0.3 (-0.34 to -0.25)
High SDI	424354 (411242-436620)	88.41 (85.63-90.92)	524907 (501601-549229)	60.27 (57.57-63)	-1.24 (-1.27 to -1.2)
High-middle SDI	451500 (432553-473005)	94.54 (90.54-98.94)	627848 (561698-694421)	68.58 (61.44-75.81)	-1.22 (-1.3 to -1.15)
Middle SDI	503551 (461761-550026)	86.36 (79.47-94.19)	1238654 (1059945-1380996)	90.72 (77.86-101.16)	0.14 (0.1 to 0.18)
Low-middle SDI	539029 (462782-617695)	150.48 (128.74-172.28)	1245784 (1010032-1435570)	160.55 (131.78-184.08)	0.18 (0.15 to 0.21)
Low SDI	144263 (119651-169531)	108.03 (89.55-127.16)	329159 (262644-392947)	111.51 (89.83-132.61)	-0.05 (-0.11 to 0.02)
High-income Asia Pacific	36698 (35587-38052)	39.27 (38.03-40.66)	67604 (62459-71675)	37.56 (34.95-39.86)	-0.51 (-0.86 to -0.16)
High-income North America	121152 (117406-124597)	82.5 (79.93-84.86)	143485 (136760-150293)	50.11 (47.79-52.39)	-1.54 (-1.71 to -1.36)
Western Europe	278852 (266480-290710)	118.73 (113.38-123.77)	227679 (212236-241951)	61.92 (57.99-65.73)	-2.17 (-2.23 to -2.1)
Australasia	7945 (7142-8774)	73.81 (66.51-81.36)	11511 (10108-13079)	49.62 (43.66-56.13)	-1.26 (-1.51 to -1.01)
Andean Latin America	3251 (2774-3808)	29.61 (24.96-34.92)	7020 (5559-8719)	23.9 (18.93-29.83)	-0.56 (-0.71 to -0.41)
Tropical Latin America	55366 (52620-58405)	113.77 (108.02-119.77)	120213 (112019-129308)	97.83 (91.27-105.16)	-0.54 (-0.67 to -0.4)
Central Latin America	17227 (16561-17867)	39.63 (38.04-41.17)	38053 (33126-43308)	32.04 (27.93-36.42)	-0.96 (-1.08 to -0.84)
Southern Latin America	17167 (15318-19145)	79.32 (70.92-88.4)	18490 (16150-20890)	48.1 (42.11-54.31)	-1.39 (-1.63 to -1.14)
Caribbean	12487 (11423-13899)	97.03 (88.86-107.82)	22804 (19311-26963)	88.76 (75.28-104.84)	-0.06 (-0.2 to 0.07)
Central Europe	101766 (97055-107160)	149.71 (142.81-157.56)	126048 (115339-136817)	141.4 (129.47-153.44)	-0.35 (-0.49 to -0.22)
Eastern Europe	180592 (172786-193942)	157.74 (151.11-169.2)	203321 (178486-229652)	146.98 (129.18-166.01)	-0.69 (-0.89 to -0.48)
Central Asia	19747 (18406-21339)	89.28 (83.18-97.23)	24967 (21747-28844)	60.81 (53.09-70.08)	-1.26 (-1.42 to -1.1)
North Africa and Middle East	22630 (19094-26888)	23.47 (19.68-27.68)	54673 (47399-62818)	20.99 (18.23-24)	-0.43 (-0.47 to -0.38)
South Asia	783369 (681307-888477)	219.81 (191.16-249.18)	1889668 (1506426-2201200)	233.17 (187.49-270.97)	0.1 (0.06 to 0.14)
Southeast Asia	112720 (96126-128911)	83.22 (71.07-95.29)	282093 (239120-325053)	83.89 (71.52-96.46)	-0.02 (-0.04 to 0)
East Asia	217546 (179390-260288)	45.99 (38.15-54.8)	556813 (434409-703385)	52.19 (40.96-65.38)	0.66 (0.52 to 0.81)
Oceania	921 (632-1236)	49.32 (34.21-64.98)	2773 (1935-3667)	57.43 (40.38-75.16)	0.7 (0.61 to 0.8)
Western Sub-Saharan Africa	10301 (8154-12614)	19.7 (15.63-24.03)	27218 (20512-34508)	23.44 (18.09-29.32)	0.52 (0.44 to 0.6)
Eastern Sub-Saharan Africa	40571 (34321-47643)	94.04 (79.66-109.72)	92061 (70193-114085)	93.15 (72.47-113.74)	-0.14 (-0.18 to -0.1)
Central Sub-Saharan Africa	6941 (5077-10308)	56.82 (42-82.51)	18602 (13759-24640)	59.54 (45.02-77.35)	0.12 (0 to 0.25)
Southern Sub-Saharan Africa	17995 (13323-22186)	131.82 (96.12-163.22)	34717 (29329-39360)	120.28 (102.71-135.44)	-0.59 (-0.85 to -0.33)

The global incidence of lip and oral cavity cancer increased from 118,387 to 272,917, with the age-standardized incidence rate (ASIR) rising from 6.17 to 6.65 (EAPC = 0.23, 95% CI: [0.16, 0.29]). Middle SDI areas had the greatest incidence rates, while Low-Middle SDI areas had the greatest ASIR (**Figure 1**). In 2021, South Asia reported the largest number of new cases, with 92,427 cases (95% UI: 73,790–107,478) (**Table 1**). South Asia had the greatest ASIR, reaching 11.98 (95% UI: 9.63–13.89), nearly 1.8 times the global average. At the country level, India had the greatest total of incidence cases (70,664, 95% UI: 55,689–83,630), followed by China (41,648, 95% UI: 31,154–54,223) and the United States (21,780, 95% UI: 20,563–22,773) (**Figure 2B**). Globally, Taiwan, China had the greatest ASIR (**Supplementary Figure S1B**).

The total number of deaths related to lip and oral cavity cancer increased from 66,990 to 136,890, although the age-standardized death rate (ASDR) decreased from 3.63 to 3.39 (EAPC = -0.28, 95% CI: [-0.32, -0.23]) (**Table 1**). In 2021, Middle SDI areas reported the greatest number of deaths, exceeding those in High SDI areas. Meanwhile, Low-Middle SDI nations showed the highest ASDR. South Asia recorded the greatest number of deaths, with 60,952 (95% UI: 49,383–70,778) fatalities and the highest ASDR at 8.26 (95% UI: 6.69–9.54). At the country level, India (45,857, 95% UI: 36,496–54,153), China (18,466, 95% UI: 13,780–23,840), and Pakistan (9,841, 95% UI: 6,809–13,411) (**Figure 2C**) accounted for approximately 50% of global deaths. This suggests a significant concentration of the burden in these countries. Interestingly, Palau had the greatest ASDR globally (**Supplementary Figure S1C**).

The DALYs reflect the trend in mortality rates. The global number of diagnosed cases of lip and oral cavity cancer increased from 2.06 million to 3.96 million, with the age-standardized disability-adjusted life year rate (ASDALYR) decreasing from 101.41 to 94.55. In 2021, Low-Middle SDI regions had the greatest number of DALYs and the greatest ASDALYR. South Asia contributed 1,889,668 (95% UI: 1,506,426–2,201,200) DALYs and had the greatest ASDALYR at 233.17 (95% UI: 187.49–270.97) (**Table 1**). At the national level, China, India, and the United States had the greatest DALYs (**Figure 2D**). Notably, Palau had the greatest ASDALYR globally (**Supplementary Figure S1D**).

### Nasopharyngeal cancer

**Figure 3:**
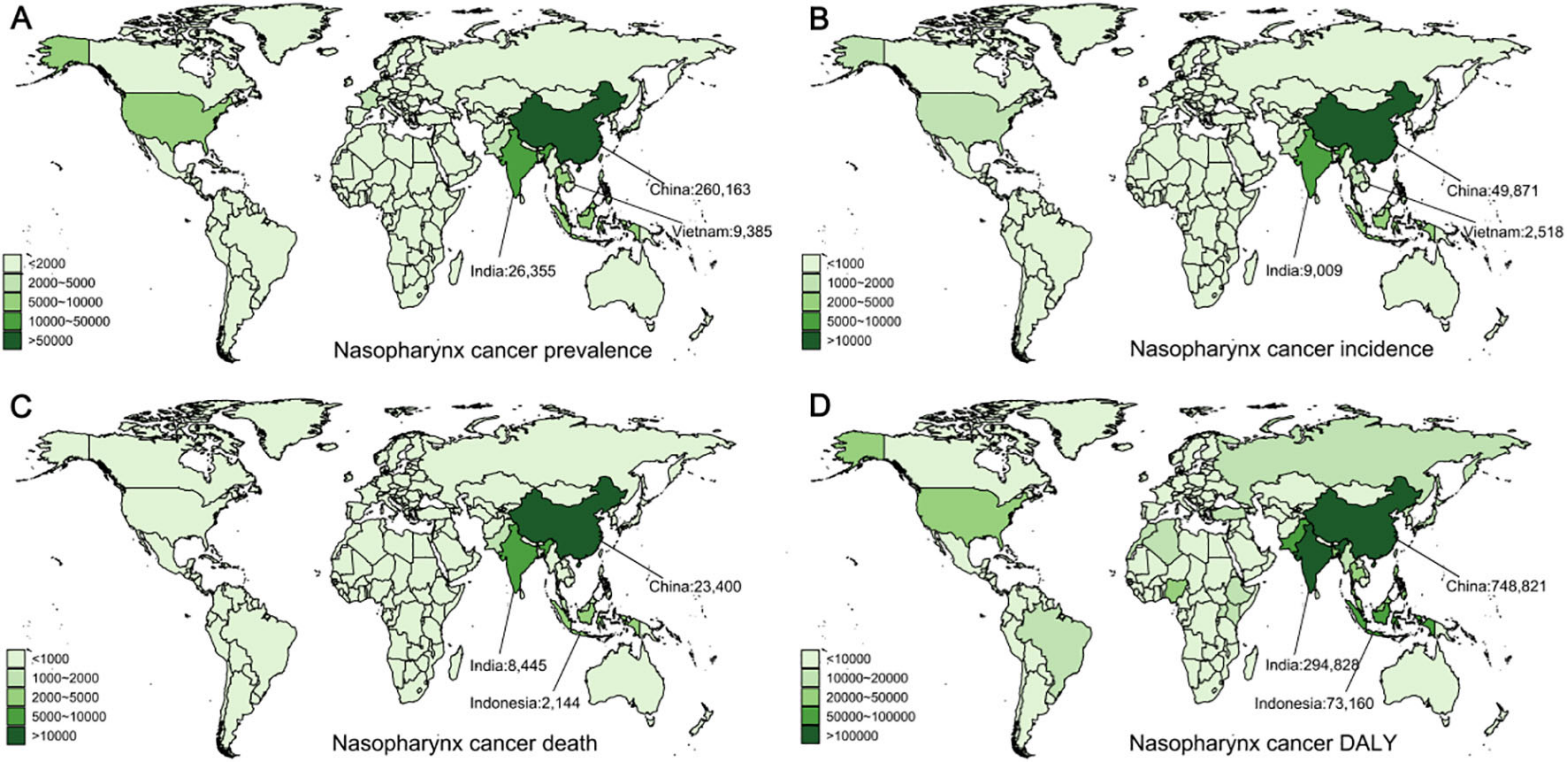
Global distribution of nasopharyngeal cancer prevalence, incidence, deaths, and disability-adjusted life years (DALYs) in 2021. **(A)** Prevalence of nasopharyngeal cancer. **(B)** Incidence of nasopharyngeal cancer. **(C)** Mortality from nasopharyngeal cancer. **(D)** DALYs from nasopharyngeal cancer.

Among the four types of male head and neck cancers, nasopharyngeal cancer (NPC) exhibits distinct regional characteristics, being most common in East Asia and Southeast Asia. The total number of nasopharyngeal cancer cases remains high and steadily increases, reaching 380,000 in 2021. The age-standardized prevalence rate (ASPR) increased from 7.58 in 1990 to 9.11 in 2021 (EAPC = 0.35, 95% CI: [0.12, 0.59]) (**Table 2**). The prevalence and ASPR were greatest in middle SDI regions. Regionally, East Asia reported the greatest number of incident cases in 2021, with 267,148 (95% UI: 203,693–341,194) and the highest ASPR of 27.28 (95% UI: 20.85–34.77), approximately three times the global norm. At the country level, China, India, and Vietnam accounted for the greatest number of NPC cases in 2021, reporting 260,163 (95% UI: 196,724–333,938), 26,355 (95% UI: 21,897–30,833), and 9,385 (95% UI: 6,670–12,669) cases, respectively (**Figure 3A**), together accounting for approximately 77% of the global total. Globally, Taiwan, China, had the greatest ASPR (**Supplementary Figure S2A**).

**Table 2:** Global and regional trends in nasopharynx cancer burden: Prevalence, incidence, mortality, and disability-adjusted life years (1990-2021).

Location	1990 prevalence cases (95% UI)	1990 ASPR (95% UI)	2021 prevalence cases (95% UI)	2021 ASPR (95% UI)	1990–2021 EAPC (95% CI)
Global	168999 (146141-190993)	7.58 (6.55-8.55)	385913 (321328-463787)	9.11 (7.6-10.94)	0.35 (0.12 to 0.59)
High SDI	27000 (25955-28167)	5.58 (5.36-5.82)	39517 (36775-42744)	5.39 (5.01-5.81)	-0.24 (-0.39 to -0.1)
High-middle SDI	57870 (47018-69701)	11.33 (9.21-13.68)	166382 (124954-217129)	19.76 (14.95-25.66)	1.45 (1.1 to 1.81)
Middle SDI	63039 (52786-72782)	9.53 (7.96-11)	138600 (113993-167238)	9.95 (8.21-11.97)	-0.08 (-0.41 to 0.24)
Low-middle SDI	15558 (12834-18461)	3.98 (3.28-4.74)	30607 (26036-35875)	3.71 (3.18-4.32)	-0.27 (-0.39 to -0.15)
Low SDI	5469 (4321-6692)	3.72 (2.94-4.56)	10683 (8145-13887)	3.2 (2.47-4.13)	-0.64 (-0.78 to -0.5)
High-income Asia Pacific	2100 (1994-2213)	2.16 (2.05-2.27)	3830 (3548-4121)	2.62 (2.41-2.85)	0.58 (0.14 to 1.02)
High-income North America	7193 (6964-7452)	4.88 (4.72-5.05)	10048 (9607-10518)	4.24 (4.06-4.45)	-0.61 (-0.71 to -0.51)
Western Europe	10829 (10273-11435)	4.85 (4.59-5.13)	10972 (9942-12271)	3.76 (3.4-4.22)	-0.96 (-1.15 to -0.77)
Australasia	1182 (1036-1336)	10.81 (9.48-12.23)	1395 (1086-1757)	7.15 (5.57-9.03)	-1.49 (-1.69 to -1.29)
Andean Latin America	54 (46-63)	0.45 (0.37-0.52)	142 (114-179)	0.47 (0.37-0.59)	0.3 (0.17 to 0.42)
Tropical Latin America	465 (435-500)	0.83 (0.77-0.89)	1373 (1262-1497)	1.11 (1.02-1.21)	0.63 (0.17 to 1.08)
Central Latin America	413 (392-435)	0.83 (0.79-0.87)	1010 (875-1172)	0.83 (0.72-0.96)	-0.38 (-0.54 to -0.21)
Southern Latin America	356 (317-404)	1.61 (1.43-1.83)	337 (268-421)	0.9 (0.71-1.12)	-1.6 (-1.7 to -1.5)
Caribbean	224 (202-248)	1.64 (1.48-1.82)	584 (493-684)	2.29 (1.93-2.68)	1.14 (0.96 to 1.32)
Central Europe	1256 (1181-1344)	1.84 (1.73-1.97)	1960 (1729-2212)	2.46 (2.18-2.78)	0.99 (0.53 to 1.46)
Eastern Europe	1776 (1635-2087)	1.54 (1.42-1.81)	1910 (1625-2245)	1.42 (1.21-1.67)	-0.61 (-0.81 to -0.41)
Central Asia	352 (315-397)	1.42 (1.27-1.59)	699 (586-840)	1.58 (1.33-1.89)	0.41 (0.18 to 0.64)
North Africa and Middle East	3381 (2873-3804)	3.03 (2.57-3.44)	8332 (7042-9816)	2.79 (2.36-3.3)	-0.34 (-0.39 to -0.3)
South Asia	17772 (15150-20910)	4.57 (3.88-5.39)	33703 (28095-39729)	3.95 (3.3-4.64)	-0.63 (-0.82 to -0.45)
Southeast Asia	11779 (9935-13898)	7.49 (6.31-8.87)	33159 (28414-38588)	9.04 (7.79-10.49)	0.47 (0.4 to 0.54)
East Asia	105802 (84849-127012)	19.38 (15.54-23.28)	267148 (203693-341194)	27.28 (20.85-34.77)	0.78 (0.35 to 1.21)
Oceania	75 (52-105)	3.78 (2.63-5.22)	156 (105-227)	3.09 (2.11-4.48)	-0.62 (-0.75 to -0.5)
Western Sub-Saharan Africa	1045 (757-1355)	1.82 (1.32-2.36)	2213 (1449-3125)	1.64 (1.1-2.29)	-0.46 (-0.58 to -0.34)
Eastern Sub-Saharan Africa	2469 (1926-3040)	5.04 (3.94-6.26)	5866 (4124-8440)	4.97 (3.51-7.12)	-0.15 (-0.21 to -0.09)
Central Sub-Saharan Africa	172 (125-232)	1.28 (0.93-1.74)	439 (291-639)	1.23 (0.83-1.78)	-0.11 (-0.23 to 0)
Southern Sub-Saharan Africa	305 (250-381)	2.08 (1.69-2.62)	636 (538-734)	2.08 (1.77-2.38)	-0.22 (-0.38 to -0.05)
	1990 incidence cases (95% UI)	1990 ASIR (95% UI)	2021 incidence cases (95% UI)	2021 ASIR (95% UI)	1990–2021 EAPC (95% CI)
Global	51320 (44302-57944)	2.42 (2.09-2.72)	86483 (73983-101789)	2.06 (1.76-2.42)	-0.85 (-1.07 to -0.63)
High SDI	6548 (6293-6834)	1.36 (1.31-1.42)	8453 (7839-9143)	1.08 (1-1.17)	-0.91 (-1.01 to -0.81)
High-middle SDI	17062 (13957-20512)	3.5 (2.86-4.2)	31674 (24343-40654)	3.69 (2.84-4.73)	-0.33 (-0.66 to 0.01)
Middle SDI	19951 (16686-23047)	3.25 (2.71-3.76)	32067 (26889-38195)	2.35 (1.97-2.78)	-1.39 (-1.67 to -1.11)
Low-middle SDI	5673 (4683-6734)	1.53 (1.26-1.82)	10497 (9014-12168)	1.33 (1.15-1.54)	-0.49 (-0.6 to -0.39)
Low SDI	2066 (1636-2524)	1.47 (1.17-1.81)	3755 (2888-4858)	1.2 (0.94-1.54)	-0.81 (-0.95 to -0.68)
High-income Asia Pacific	654 (620-689)	0.7 (0.66-0.73)	1211 (1115-1299)	0.7 (0.65-0.76)	-0.17 (-0.54 to 0.2)
High-income North America	1471 (1425-1525)	1 (0.97-1.03)	1914 (1826-2007)	0.77 (0.73-0.8)	-0.99 (-1.06 to -0.93)
Western Europe	2712 (2577-2854)	1.19 (1.13-1.25)	2340 (2116-2621)	0.73 (0.67-0.82)	-1.68 (-1.79 to -1.56)
Australasia	216 (192-242)	1.99 (1.76-2.23)	241 (187-302)	1.19 (0.93-1.5)	-1.81 (-1.96 to -1.66)
Andean Latin America	20 (17-23)	0.18 (0.15-0.21)	48 (38-60)	0.16 (0.13-0.21)	-0.11 (-0.24 to 0.01)
Tropical Latin America	156 (146-169)	0.29 (0.27-0.32)	427 (392-465)	0.35 (0.32-0.38)	0.23 (-0.22 to 0.68)
Central Latin America	148 (140-155)	0.32 (0.31-0.34)	338 (293-392)	0.28 (0.25-0.33)	-0.78 (-0.94 to -0.62)
Southern Latin America	124 (110-141)	0.58 (0.51-0.66)	103 (82-130)	0.27 (0.22-0.34)	-2.11 (-2.21 to -2)
Caribbean	81 (73-90)	0.62 (0.56-0.69)	201 (169-237)	0.79 (0.67-0.93)	0.85 (0.7 to 1)
Central Europe	432 (406-463)	0.64 (0.6-0.69)	604 (536-681)	0.71 (0.63-0.8)	0.38 (-0.06 to 0.81)
Eastern Europe	667 (615-780)	0.6 (0.55-0.7)	716 (611-840)	0.53 (0.45-0.62)	-0.81 (-1.04 to -0.57)
Central Asia	126 (113-141)	0.55 (0.49-0.62)	237 (200-284)	0.58 (0.49-0.69)	0.21 (0.02 to 0.41)
North Africa and Middle East	1154 (980-1311)	1.12 (0.94-1.29)	2375 (1999-2805)	0.87 (0.73-1.02)	-0.96 (-1.01 to -0.9)
South Asia	6482 (5506-7632)	1.75 (1.48-2.08)	11523 (9659-13498)	1.41 (1.19-1.65)	-0.87 (-1.04 to -0.7)
Southeast Asia	3906 (3293-4621)	2.7 (2.27-3.2)	9549 (8189-11067)	2.74 (2.37-3.16)	-0.07 (-0.12 to -0.02)
East Asia	31440 (25219-37755)	6.27 (5.04-7.51)	51415 (39385-65470)	5.14 (3.95-6.53)	-1.15 (-1.55 to -0.75)
Oceania	27 (18-37)	1.44 (1.01-1.97)	54 (36-78)	1.14 (0.77-1.65)	-0.7 (-0.84 to -0.57)
Western Sub-Saharan Africa	384 (279-500)	0.7 (0.51-0.91)	757 (502-1059)	0.6 (0.4-0.83)	-0.65 (-0.76 to -0.53)
Eastern Sub-Saharan Africa	942 (734-1161)	2.03 (1.59-2.52)	2040 (1441-2924)	1.87 (1.34-2.66)	-0.41 (-0.47 to -0.36)
Central Sub-Saharan Africa	67 (49-91)	0.53 (0.39-0.73)	160 (107-232)	0.49 (0.34-0.71)	-0.28 (-0.38 to -0.18)
Southern Sub-Saharan Africa	111 (91-140)	0.81 (0.66-1.03)	230 (196-264)	0.81 (0.7-0.92)	-0.24 (-0.49 to 0.01)
	1990 death cases (95% UI)	1990 ASDR (95% UI)	2021 death cases (95% UI)	2021 ASDR (95% UI)	1990–2021 EAPC (95% CI)
Global	43851 (37811-49404)	2.14 (1.85-2.41)	53937 (47076-61333)	1.3 (1.14-1.47)	-2 (-2.18 to -1.81)
High SDI	4481 (4279-4696)	0.94 (0.9-0.98)	4940 (4578-5371)	0.56 (0.52-0.61)	-1.86 (-1.94 to -1.79)
High-middle SDI	14227 (11644-17062)	3.03 (2.48-3.62)	14832 (11887-18461)	1.66 (1.33-2.06)	-2.54 (-2.78 to -2.3)
Middle SDI	17611 (14762-20405)	3.02 (2.54-3.5)	20698 (17714-24228)	1.55 (1.33-1.81)	-2.55 (-2.78 to -2.32)
Low-middle SDI	5491 (4545-6517)	1.52 (1.26-1.81)	9856 (8478-11452)	1.29 (1.12-1.5)	-0.59 (-0.68 to -0.49)
Low SDI	2024 (1608-2474)	1.48 (1.18-1.82)	3579 (2759-4633)	1.2 (0.94-1.53)	-0.84 (-0.97 to -0.71)
High-income Asia Pacific	575 (544-605)	0.63 (0.6-0.66)	1109 (1017-1185)	0.56 (0.52-0.6)	-0.72 (-1.05 to -0.39)
High-income North America	763 (738-788)	0.52 (0.5-0.54)	867 (821-912)	0.31 (0.29-0.33)	-1.79 (-1.89 to -1.69)
Western Europe	1927 (1833-2031)	0.82 (0.78-0.87)	1353 (1229-1512)	0.36 (0.33-0.41)	-2.76 (-2.83 to -2.69)
Australasia	88 (79-98)	0.82 (0.74-0.91)	87 (68-107)	0.38 (0.29-0.47)	-2.6 (-2.68 to -2.52)
Andean Latin America	20 (17-23)	0.19 (0.16-0.22)	45 (36-57)	0.16 (0.13-0.2)	-0.32 (-0.45 to -0.19)
Tropical Latin America	144 (134-155)	0.28 (0.26-0.3)	375 (344-410)	0.31 (0.28-0.34)	-0.01 (-0.46 to 0.44)
Central Latin America	143 (136-151)	0.33 (0.31-0.35)	316 (274-364)	0.27 (0.24-0.31)	-1 (-1.16 to -0.84)
Southern Latin America	117 (103-133)	0.56 (0.49-0.64)	90 (72-113)	0.24 (0.19-0.3)	-2.42 (-2.54 to -2.31)
Caribbean	79 (70-88)	0.63 (0.56-0.7)	190 (160-225)	0.75 (0.63-0.88)	0.68 (0.54 to 0.81)
Central Europe	403 (378-433)	0.6 (0.57-0.65)	525 (467-589)	0.59 (0.53-0.67)	-0.04 (-0.46 to 0.38)
Eastern Europe	655 (604-762)	0.6 (0.55-0.69)	705 (604-829)	0.52 (0.45-0.61)	-0.87 (-1.1 to -0.63)
Central Asia	121 (108-136)	0.55 (0.49-0.63)	221 (187-264)	0.56 (0.48-0.67)	0.11 (-0.08 to 0.3)
North Africa and Middle East	1082 (913-1235)	1.11 (0.93-1.28)	1956 (1645-2296)	0.77 (0.65-0.89)	-1.33 (-1.4 to -1.27)
South Asia	6279 (5315-7411)	1.75 (1.47-2.07)	10814 (9093-12633)	1.36 (1.15-1.59)	-0.97 (-1.13 to -0.81)
Southeast Asia	3571 (3001-4225)	2.6 (2.19-3.09)	7875 (6741-9101)	2.36 (2.03-2.71)	-0.42 (-0.47 to -0.38)
East Asia	26388 (21162-31630)	5.62 (4.54-6.69)	24349 (18793-30705)	2.33 (1.82-2.92)	-3.45 (-3.74 to -3.15)
Oceania	25 (17-35)	1.42 (1-1.95)	50 (34-74)	1.12 (0.75-1.62)	-0.72 (-0.86 to -0.58)
Western Sub-Saharan Africa	375 (274-487)	0.7 (0.52-0.91)	709 (473-984)	0.59 (0.4-0.8)	-0.72 (-0.83 to -0.61)
Eastern Sub-Saharan Africa	921 (720-1135)	2.05 (1.6-2.54)	1926 (1357-2763)	1.85 (1.33-2.63)	-0.46 (-0.51 to -0.41)
Central Sub-Saharan Africa	66 (48-90)	0.55 (0.4-0.75)	154 (104-224)	0.5 (0.34-0.73)	-0.31 (-0.4 to -0.21)
Southern Sub-Saharan Africa	107 (87-136)	0.82 (0.66-1.04)	218 (187-251)	0.8 (0.7-0.91)	-0.29 (-0.55 to -0.03)
	1990 DALY cases (95% UI)	1990 ASDALYR (95% UI)	2021 DALY cases (95% UI)	2021ASDALYR (95% UI)	1990–2021 EAPC (95% CI)
Global	1597695 (1380769-1798792)	72.12 (62.35-81.31)	1788775 (1552540-2053374)	42.26 (36.7-48.46)	-2.1 (-2.29 to -1.91)
High SDI	150569 (143793-157816)	31.22 (29.81-32.73)	140072 (129753-152082)	17.42 (16.15-18.9)	-2.12 (-2.2 to -2.04)
High-middle SDI	509727 (418364-611099)	101.28 (83.09-121.23)	483563 (384845-602273)	54.75 (43.69-67.9)	-2.62 (-2.87 to -2.36)
Middle SDI	654796 (552013-753951)	99.26 (83.32-114.61)	679427 (581267-793453)	48.92 (41.94-57.05)	-2.66 (-2.9 to -2.43)
Low-middle SDI	205570 (170634-243260)	50.92 (42.11-60.44)	348901 (296294-408492)	42.11 (36.01-49.03)	-0.65 (-0.75 to -0.56)
Low SDI	76383 (60355-92720)	49.65 (39.44-60.83)	135772 (102982-178175)	39.13 (30.04-50.77)	-0.94 (-1.06 to -0.82)
High-income Asia Pacific	18689 (17608-19696)	19.3 (18.21-20.31)	24564 (22856-26271)	14.58 (13.65-15.59)	-1.26 (-1.58 to -0.94)
High-income North America	24506 (23750-25308)	16.79 (16.28-17.34)	25535 (24246-26740)	9.89 (9.42-10.35)	-1.84 (-1.94 to -1.74)
Western Europe	61590 (58596-64928)	26.81 (25.5-28.28)	37440 (33984-41817)	11.22 (10.22-12.49)	-2.97 (-3.04 to -2.9)
Australasia	2801 (2504-3104)	25.85 (23.11-28.68)	2530 (1970-3135)	12.11 (9.44-15.08)	-2.52 (-2.59 to -2.44)
Andean Latin America	710 (598-817)	5.5 (4.62-6.34)	1342 (1064-1677)	4.44 (3.53-5.56)	-0.56 (-0.7 to -0.42)
Tropical Latin America	5742 (5368-6194)	10.02 (9.34-10.8)	13344 (12216-14568)	10.79 (9.89-11.79)	-0.06 (-0.52 to 0.39)
Central Latin America	4988 (4743-5254)	9.6 (9.13-10.11)	9466 (8181-10967)	7.76 (6.71-8.98)	-1.07 (-1.22 to -0.92)
Southern Latin America	3773 (3341-4278)	17.09 (15.14-19.41)	2674 (2145-3335)	7.09 (5.7-8.86)	-2.54 (-2.65 to -2.44)
Caribbean	2443 (2184-2749)	17.92 (16.05-20.13)	5558 (4653-6566)	21.73 (18.2-25.69)	0.69 (0.55 to 0.84)
Central Europe	13803 (12938-14793)	20.24 (18.96-21.74)	16015 (14235-17979)	19.06 (16.92-21.44)	-0.2 (-0.63 to 0.23)
Eastern Europe	23086 (21303-26945)	20.12 (18.56-23.5)	22798 (19484-26732)	17.11 (14.64-20.05)	-0.97 (-1.22 to -0.72)
Central Asia	4754 (4229-5380)	18.37 (16.42-20.62)	8120 (6802-9876)	18.39 (15.49-22.27)	0 (-0.17 to 0.18)
North Africa and Middle East	40818 (34650-46035)	35.5 (30.14-40.33)	67784 (56914-80202)	23.55 (19.72-27.78)	-1.46 (-1.51 to -1.41)
South Asia	238044 (202445-279004)	59.11 (50.17-69.63)	382479 (318686-448642)	44.71 (37.35-52.36)	-1.05 (-1.19 to -0.9)
Southeast Asia	131201 (110848-155211)	83.65 (70.52-99.07)	269220 (229578-314036)	74.66 (63.77-86.98)	-0.49 (-0.54 to -0.44)
East Asia	964322 (773733-1154529)	180.64 (145.12-216.9)	780121 (597089-986478)	74.84 (57.63-94.61)	-3.46 (-3.78 to -3.15)
Oceania	880 (598-1230)	44.17 (30.38-61.3)	1759 (1174-2603)	34.73 (23.43-50.86)	-0.74 (-0.88 to -0.6)
Western Sub-Saharan Africa	14083 (10240-18321)	23.73 (17.32-30.87)	28263 (18457-39621)	20.3 (13.49-28.18)	-0.64 (-0.76 to -0.53)
Eastern Sub-Saharan Africa	35282 (27488-43280)	68.04 (53.15-84.08)	76457 (53717-110259)	61.79 (43.45-88.47)	-0.47 (-0.52 to -0.41)
Central Sub-Saharan Africa	2392 (1747-3219)	16.99 (12.39-23.01)	5722 (3769-8358)	15.38 (10.35-22.27)	-0.33 (-0.43 to -0.23)
Southern Sub-Saharan Africa	3788 (3118-4778)	25.08 (20.41-31.81)	7582 (6405-8769)	24.58 (21.03-28.3)	-0.3 (-0.56 to -0.03)

The number of incidence NPC cases globally increased from 51,320 to 86,483, with the age-standardized incidence rate (ASIR) decreasing from 2.42 to 2.06 (EAPC = -0.85, 95% CI: [-1.07, -0.63]). The greatest incidence rate was observed in middle SDI regions, while the highest ASIR occurred in high SDI regions (**Figure 3B**). In 2021, East Asia recorded the highest number of incidence cases, totaling 51,415 (95% UI: 39,385–65,470) (**Table 2**). East Asia had the greatest ASIR, reaching 5.14 (95% UI: 3.95–6.53), which is 2.5 times higher than the global standard. At the country level, China reported the greatest number of new cases (49,871, 95% UI: 37,877–63,915), followed by India (9,009, 95% UI: 7,541–10,523) and Vietnam (2,518, 95% UI: 1,789–3,400) (**Figure 3B**), with these three countries accounting for 71% of global cases. Globally, Malaysia had the greatest ASIR (**Supplementary Figure S2B**).

Deaths from NPC rose from 43,851 in 1990 to 53,936 in 2021, while the age-standardized death rate (ASDR) decreased from 2.14 to 1.30 (EAPC = -2.00, 95% CI: [-2.18, -1.81]) (**Table 2**). Middle SDI regions had the largest number of deaths, while the highest ASDR was consistently observed in high SDI regions. Geographically, East Asia reported the greatest total of deaths in 2021, and Southeast Asia had the greatest ASDR globally. At the country level, China (23,400, 95% UI: 17,889–29,725), India (8,445, 95% UI: 7,063–9,883), and Indonesia (2,144, 95% UI: 1,385–3,253) reported the most deaths in 2021 (**Figure 3C**), with these three countries accounting for 63% of global deaths. Globally, Malaysia had the greatest ASDR (**Supplementary Figure S2C**).

The DALYs for NPC mirror the trend in mortality. The global number of DALYs from NPC rose from 1.59 million in 1990 to 1.78 million in 2021, while the age-standardized disability-adjusted life year rate (ASDALYR) dropped from 72.12 to 42.26 (EAPC = -2.1, 95% CI: [-2.29, -1.91]) (**Table 2**). The highest ASDALYR is consistently recorded by middle SDI regions. Regionally, East Asia reported the greatest DALYs and ASDALYR in 2021, both globally (**Table 2**). Notably, the Caribbean continues to be the sole region where all four indicators are at the highest level. At the country level, China (748,821, 95% UI: 566,352–952,744), India (294,828, 95% UI: 245,674–346,224), and Indonesia (73,160, 95% UI: 45,659–114,147) reported the greatest number of nasopharyngeal cancer cases in 2021 (**Figure 3D**), with these three countries accounting for approximately 63% of global cases. Globally, Malaysia maintains the greatest ASDALYR for nasopharyngeal cancer (**Supplementary Figure S2D**).

### Laryngeal cancer

Among the four types of male head and neck cancers, laryngeal cancer (LC) ranks second in terms of global cancer impact, second only to lip and oral cavity cancer.

**Figure 4:**
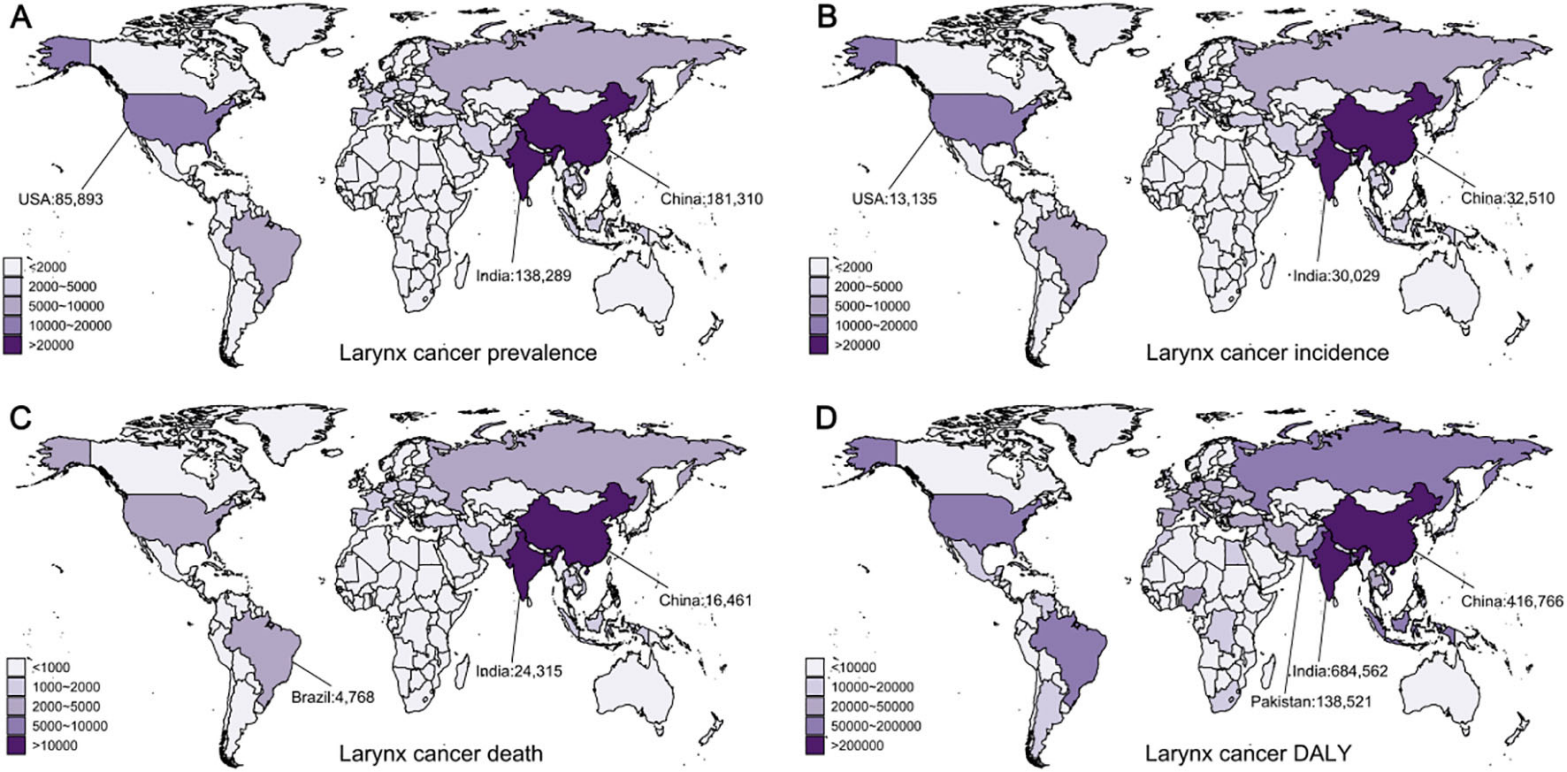
Global distribution of laryngeal cancer prevalence, incidence, deaths, and disability-adjusted life years (DALYs) in 2021. **(A)** Prevalence of laryngeal cancer. **(B)** Incidence of laryngeal cancer. **(C)** Mortality from laryngeal cancer. **(D)** DALYs from laryngeal cancer.

Between 1990 and 2021, the global prevalence of LC rose from 549,371 to 939,924, while the age-standardized prevalence rate (ASPR) decreased from 28.43 to 22.54 (EAPC = -0.88, 95% CI: [-0.93, -0.82]) (**Table 3**). In middle SDI regions, the incidence of laryngeal cancer increased rapidly, exceeding that of high SDI regions in 2021, leading in total prevalent cases, and reporting the highest ASPR for LC. East Asia accounted for the greatest number of cases in 2021 (187,471, 95% UI: 140,156–240,459), whereas Central Europe had the greatest ASPR at 48.04 (95% UI: 43.99–52.29). In 2021, China had the highest number of new laryngeal cancer cases globally, with 181,310 (95% UI: 133,833–234,747), followed by India with 138,289 (95% UI: 118,080–161,374), and the United States with 85,893 (95% UI: 81,762–89,567) (**Figure 4A**). Together, these nations made up around 43% of the world’s new cases. Monaco observed the greatest ASPR globally (**Supplementary Figure S3A**).

**Table 3:** Global and regional trends in larynx cancer burden: Prevalence, incidence, mortality, and disability-adjusted life years (1990-2021).

Location	1990 prevalence cases (95% UI)	1990 ASPR (95% UI)	2021 prevalence cases (95% UI)	2021 ASPR (95% UI)	1990–2021 EAPC (95% CI)
Global	549372 (522107-579828)	28.43 (27-29.99)	939924 (876345-1011203)	22.54 (21.03-24.21)	-0.88 (-0.93 to -0.82)
High SDI	197515 (190815-204787)	41.01 (39.57-42.55)	253770 (241267-264228)	27.33 (26.03-28.43)	-1.41 (-1.51 to -1.32)
High-middle SDI	182391 (174158-191553)	39.02 (37.23-41.06)	258381 (231879-287217)	27.65 (24.86-30.71)	-1.29 (-1.37 to -1.2)
Middle SDI	87797 (80058-95705)	16.38 (14.98-17.82)	246727 (217312-278858)	18.24 (16.12-20.52)	0.28 (0.19 to 0.37)
Low-middle SDI	62194 (53422-73054)	18.76 (16.08-21.98)	142671 (128200-160558)	19.53 (17.6-21.89)	0.08 (-0.01 to 0.17)
Low SDI	18661 (15223-23118)	15.07 (12.37-18.56)	37136 (31597-43256)	14.06 (12.07-16.35)	-0.36 (-0.46 to -0.26)
High-income Asia Pacific	25220 (23029-27304)	27.69 (25.36-29.9)	36798 (32695-40801)	18.27 (16.17-20.42)	-1.56 (-1.78 to -1.33)
High-income North America	66852 (64523-69022)	45 (43.45-46.51)	93239 (88943-97022)	31.01 (29.62-32.25)	-1.47 (-1.6 to -1.34)
Western Europe	140726 (134326-146996)	58.25 (55.62-60.86)	141980 (133757-150816)	36.42 (34.32-38.67)	-1.53 (-1.62 to -1.44)
Australasia	2251 (2059-2477)	20.81 (19.02-22.91)	2998 (2651-3350)	12.58 (11.06-14.16)	-1.58 (-1.7 to -1.45)
Andean Latin America	793 (677-922)	8.02 (6.87-9.32)	1715 (1312-2217)	6.07 (4.66-7.82)	-1.14 (-1.43 to -0.85)
Tropical Latin America	12085 (11557-12629)	25.96 (24.8-27.13)	33986 (31700-36297)	27.86 (26-29.74)	0.17 (0.07 to 0.27)
Central Latin America	6111 (5896-6334)	15.19 (14.65-15.76)	12047 (10536-13888)	10.32 (9.04-11.87)	-1.71 (-1.87 to -1.55)
Southern Latin America	8676 (7970-9440)	40.34 (37.1-43.95)	8968 (8140-9888)	23.04 (20.93-25.39)	-1.94 (-2.12 to -1.75)
Caribbean	3609 (3296-3991)	28.47 (26.02-31.45)	9649 (8213-11391)	37.44 (32-44.11)	1.03 (0.89 to 1.16)
Central Europe	33403 (31560-35659)	48.37 (45.74-51.58)	44783 (41006-48749)	48.04 (43.99-52.29)	-0.12 (-0.27 to 0.04)
Eastern Europe	60726 (58383-63322)	53.43 (51.26-55.83)	51238 (45433-57328)	36.1 (32.08-40.32)	-1.7 (-1.91 to -1.49)
Central Asia	6553 (6275-6869)	30.45 (29.17-31.89)	5709 (5138-6314)	14.31 (12.92-15.79)	-2.45 (-2.53 to -2.37)
North Africa and Middle East	18609 (15668-22003)	20.74 (17.62-24.64)	55655 (48926-62945)	23.24 (20.53-26.24)	0.35 (0.3 to 0.41)
South Asia	76427 (64819-90484)	23.28 (19.71-27.56)	178908 (154922-206722)	23.28 (20.2-26.89)	-0.17 (-0.3 to -0.04)
Southeast Asia	14915 (12890-16791)	11.92 (10.31-13.45)	48479 (41511-56853)	14.87 (12.85-17.33)	0.72 (0.66 to 0.77)
East Asia	59986 (48776-71404)	13.41 (10.96-15.82)	187471 (140156-240459)	16.88 (12.75-21.52)	0.9 (0.74 to 1.06)
Oceania	67 (50-88)	4.43 (3.37-5.73)	158 (120-208)	4.09 (3.18-5.36)	-0.34 (-0.4 to -0.29)
Western Sub-Saharan Africa	4265 (3413-5275)	8.95 (7.24-10.92)	9823 (7742-11962)	9.96 (8.01-12)	0.48 (0.41 to 0.55)
Eastern Sub-Saharan Africa	4369 (3545-5324)	10.86 (8.86-13.16)	8405 (6442-10980)	9.44 (7.32-12.07)	-0.62 (-0.69 to -0.56)
Central Sub-Saharan Africa	1341 (965-1773)	11.87 (8.85-15.46)	3110 (2290-4066)	11.28 (8.58-14.55)	-0.2 (-0.34 to -0.05)
Southern Sub-Saharan Africa	2388 (1929-3108)	18.87 (15.3-24.53)	4806 (4161-5514)	17.87 (15.65-20.33)	-0.43 (-0.63 to -0.23)
	1990 incidence cases (95% UI)	1990 ASIR (95% UI)	2021 incidence cases (95% UI)	2021 ASIR (95% UI)	1990–2021 EAPC (95% CI)
Global	109879 (104446-116063)	5.8 (5.5-6.12)	171789 (159470-186042)	4.16 (3.86-4.5)	-1.21 (-1.29 to -1.14)
High SDI	33876 (32820-34981)	7.08 (6.85-7.31)	38928 (36852-40809)	4.18 (3.96-4.37)	-1.81 (-1.88 to -1.74)
High-middle SDI	36508 (34844-38261)	7.98 (7.6-8.39)	44810 (40075-49889)	4.84 (4.33-5.38)	-1.82 (-1.92 to -1.73)
Middle SDI	19763 (18053-21543)	3.87 (3.54-4.2)	47811 (41909-54188)	3.64 (3.21-4.1)	-0.29 (-0.37 to -0.21)
Low-middle SDI	14917 (12739-17680)	4.65 (3.96-5.5)	31439 (28001-35495)	4.43 (3.95-4.98)	-0.19 (-0.27 to -0.12)
Low SDI	4648 (3705-5805)	3.9 (3.11-4.83)	8574 (7202-10071)	3.38 (2.86-3.96)	-0.57 (-0.67 to -0.48)
High-income Asia Pacific	4089 (3715-4445)	4.57 (4.16-4.95)	5458 (4764-6124)	2.67 (2.32-3.02)	-2 (-2.21 to -1.79)
High-income North America	10888 (10575-11186)	7.33 (7.11-7.53)	14187 (13440-14746)	4.71 (4.47-4.9)	-1.71 (-1.82 to -1.6)
Western Europe	24604 (23553-25630)	10.21 (9.78-10.64)	21301 (19885-22744)	5.43 (5.08-5.8)	-2.03 (-2.11 to -1.96)
Australasia	418 (382-461)	3.9 (3.56-4.29)	476 (416-540)	1.98 (1.72-2.25)	-2.16 (-2.27 to -2.04)
Andean Latin America	199 (170-232)	2.08 (1.77-2.42)	372 (283-477)	1.34 (1.02-1.71)	-1.63 (-1.91 to -1.34)
Tropical Latin America	2736 (2617-2863)	6.06 (5.79-6.34)	6851 (6381-7309)	5.7 (5.3-6.08)	-0.22 (-0.32 to -0.13)
Central Latin America	1463 (1408-1516)	3.77 (3.62-3.91)	2573 (2247-2972)	2.25 (1.97-2.59)	-2.14 (-2.28 to -1.99)
Southern Latin America	1760 (1621-1921)	8.25 (7.61-9.01)	1593 (1424-1765)	4.1 (3.66-4.54)	-2.33 (-2.49 to -2.16)
Caribbean	798 (726-883)	6.39 (5.82-7.06)	1919 (1623-2267)	7.5 (6.35-8.85)	0.63 (0.51 to 0.75)
Central Europe	6881 (6500-7329)	10.07 (9.51-10.7)	8077 (7338-8830)	8.62 (7.83-9.42)	-0.63 (-0.77 to -0.49)
Eastern Europe	12566 (12109-13037)	11.21 (10.75-11.65)	9523 (8356-10747)	6.72 (5.9-7.59)	-2.17 (-2.38 to -1.96)
Central Asia	1449 (1386-1520)	6.92 (6.62-7.26)	1148 (1027-1282)	2.96 (2.65-3.29)	-2.74 (-2.84 to -2.64)
North Africa and Middle East	4119 (3418-4935)	4.78 (4-5.8)	10231 (8965-11647)	4.42 (3.9-5.05)	-0.28 (-0.35 to -0.22)
South Asia	18262 (15279-21694)	5.77 (4.8-6.87)	39014 (33487-45232)	5.22 (4.49-6.03)	-0.48 (-0.6 to -0.36)
Southeast Asia	3320 (2836-3764)	2.76 (2.35-3.13)	9449 (8058-11066)	3.01 (2.59-3.5)	0.25 (0.21 to 0.3)
East Asia	13264 (10659-15871)	3.16 (2.58-3.77)	33549 (25168-43354)	3.12 (2.37-4)	0.06 (-0.09 to 0.2)
Oceania	16 (11-21)	1.12 (0.83-1.48)	36 (27-48)	1 (0.76-1.35)	-0.44 (-0.5 to -0.39)
Western Sub-Saharan Africa	1043 (820-1301)	2.28 (1.82-2.81)	2274 (1786-2785)	2.42 (1.95-2.91)	0.34 (0.27 to 0.4)
Eastern Sub-Saharan Africa	1125 (902-1383)	2.89 (2.33-3.54)	1955 (1480-2557)	2.29 (1.75-2.94)	-0.96 (-1.03 to -0.89)
Central Sub-Saharan Africa	346 (244-463)	3.21 (2.35-4.2)	738 (535-978)	2.86 (2.14-3.68)	-0.42 (-0.55 to -0.29)
Southern Sub-Saharan Africa	532 (432-702)	4.3 (3.51-5.71)	1066 (915-1220)	4.09 (3.56-4.64)	-0.45 (-0.77 to -0.14)
	1990 death cases (95% UI)	1990 ASDR (95% UI)	2021 death cases (95% UI)	2021 ASDR (95% UI)	1990–2021 EAPC (95% CI)
Global	75262 (70749-80447)	4.12 (3.87-4.4)	100393 (93351-108830)	2.49 (2.31-2.69)	-1.79 (-1.87 to -1.71)
High SDI	15292 (14792-15800)	3.27 (3.15-3.38)	13244 (12384-13910)	1.39 (1.3-1.45)	-2.9 (-2.98 to -2.81)
High-middle SDI	25181 (23841-26515)	5.75 (5.44-6.08)	22964 (20765-25348)	2.56 (2.32-2.81)	-2.88 (-2.98 to -2.79)
Middle SDI	16643 (15185-18119)	3.44 (3.15-3.73)	30547 (27128-34294)	2.43 (2.17-2.72)	-1.24 (-1.29 to -1.19)
Low-middle SDI	13669 (11627-16213)	4.41 (3.74-5.23)	25958 (23049-29145)	3.79 (3.37-4.25)	-0.52 (-0.57 to -0.47)
Low SDI	4356 (3500-5431)	3.79 (3.07-4.7)	7545 (6317-8883)	3.12 (2.63-3.66)	-0.72 (-0.8 to -0.64)
High-income Asia Pacific	1443 (1284-1596)	1.74 (1.56-1.91)	1476 (1282-1643)	0.66 (0.57-0.73)	-3.61 (-3.78 to -3.43)
High-income North America	3755 (3633-3856)	2.54 (2.45-2.61)	4038 (3818-4222)	1.33 (1.26-1.39)	-2.33 (-2.4 to -2.27)
Western Europe	12178 (11692-12651)	5.11 (4.89-5.3)	7908 (7316-8428)	1.9 (1.76-2.02)	-3.22 (-3.34 to -3.11)
Australasia	251 (229-278)	2.39 (2.19-2.64)	230 (199-259)	0.9 (0.79-1.02)	-3.22 (-3.32 to -3.11)
Andean Latin America	188 (161-218)	2.02 (1.73-2.35)	293 (224-371)	1.08 (0.83-1.36)	-2.21 (-2.46 to -1.97)
Tropical Latin America	2261 (2161-2362)	5.2 (4.96-5.44)	4855 (4535-5165)	4.14 (3.85-4.4)	-0.71 (-0.8 to -0.61)
Central Latin America	1296 (1247-1339)	3.46 (3.32-3.58)	1948 (1702-2233)	1.75 (1.53-1.99)	-2.64 (-2.77 to -2.5)
Southern Latin America	1311 (1217-1430)	6.28 (5.83-6.84)	1009 (909-1107)	2.61 (2.35-2.87)	-2.79 (-2.94 to -2.64)
Caribbean	626 (571-691)	5.1 (4.66-5.62)	1264 (1078-1490)	5 (4.28-5.88)	0.02 (-0.08 to 0.13)
Central Europe	5172 (4899-5493)	7.75 (7.34-8.22)	4765 (4350-5190)	5.02 (4.58-5.47)	-1.56 (-1.67 to -1.45)
Eastern Europe	9111 (8799-9416)	8.4 (8.09-8.7)	5662 (4979-6405)	4.06 (3.57-4.59)	-2.92 (-3.13 to -2.72)
Central Asia	1183 (1136-1238)	5.88 (5.65-6.16)	853 (764-951)	2.33 (2.09-2.59)	-2.99 (-3.11 to -2.86)
North Africa and Middle East	3365 (2800-4061)	4.11 (3.45-5.01)	5852 (5133-6683)	2.7 (2.38-3.08)	-1.42 (-1.47 to -1.36)
South Asia	16684 (13883-19894)	5.48 (4.54-6.55)	31963 (27536-36964)	4.43 (3.84-5.1)	-0.82 (-0.91 to -0.74)
Southeast Asia	2727 (2348-3093)	2.39 (2.04-2.71)	6069 (5263-7040)	2.06 (1.79-2.39)	-0.51 (-0.54 to -0.49)
East Asia	10874 (8739-12986)	2.78 (2.28-3.3)	16920 (12730-21679)	1.66 (1.27-2.1)	-1.68 (-1.77 to -1.59)
Oceania	13 (10-18)	1.06 (0.78-1.39)	30 (23-41)	0.92 (0.7-1.25)	-0.49 (-0.53 to -0.45)
Western Sub-Saharan Africa	982 (776-1222)	2.24 (1.79-2.77)	2025 (1619-2462)	2.26 (1.85-2.71)	0.2 (0.13 to 0.27)
Eastern Sub-Saharan Africa	1058 (854-1302)	2.82 (2.28-3.44)	1724 (1307-2234)	2.12 (1.63-2.7)	-1.11 (-1.17 to -1.04)
Central Sub-Saharan Africa	328 (232-437)	3.18 (2.35-4.15)	657 (475-866)	2.73 (2.04-3.54)	-0.53 (-0.64 to -0.42)
Southern Sub-Saharan Africa	453 (368-594)	3.83 (3.13-5.02)	853 (736-978)	3.44 (3-3.9)	-0.62 (-0.98 to -0.26)
	1990 DALY cases (95% UI)	1990 ASDALYR (95% UI)	2021 DALY cases(95% UI)	2021ASDALYR (95% UI)	1990–2021 EAPC (95% CI)
Global	2181146 (2048786-2333042)	110.04 (103.4-117.38)	2694179 (2491890-2926037)	64.13 (59.35-69.57)	-1.93 (-2.01 to -1.84)
High SDI	418631 (405149-432993)	87.04 (84.21-90.01)	316573 (298930-332917)	34.6 (32.71-36.35)	-3.1 (-3.17 to -3.02)
High-middle SDI	742007 (704808-779520)	156.56 (148.47-164.63)	601731 (541101-663944)	64.7 (58.29-71.21)	-3.18 (-3.29 to -3.06)
Middle SDI	484303 (441300-528492)	87.9 (80.17-95.62)	821659 (727232-923094)	60.69 (53.76-68.18)	-1.33 (-1.38 to -1.28)
Low-middle SDI	403783 (344209-479413)	118.26 (100.82-140.22)	732917 (650192-831897)	98.61 (87.5-111.37)	-0.62 (-0.67 to -0.56)
Low SDI	128901 (103065-161826)	101.14 (81.28-126.53)	217735 (181976-256634)	79.81 (66.97-93.78)	-0.91 (-0.99 to -0.83)
High-income Asia Pacific	37272 (32667-41798)	41.16 (36.27-45.85)	28774 (25325-32285)	14.06 (12.35-15.9)	-3.88 (-4.03 to -3.72)
High-income North America	98490 (95442-101475)	66.66 (64.53-68.72)	98640 (93951-103402)	33.28 (31.8-34.85)	-2.49 (-2.55 to -2.42)
Western Europe	329380 (315817-343360)	137.58 (131.83-143.48)	183640 (170754-195860)	47.42 (44.2-50.55)	-3.47 (-3.58 to -3.37)
Australasia	6473 (5872-7156)	59.85 (54.39-66.1)	5054 (4440-5722)	20.91 (18.37-23.73)	-3.43 (-3.54 to -3.33)
Andean Latin America	4722 (4040-5503)	46.63 (39.88-54.21)	6833 (5175-8697)	24.08 (18.31-30.59)	-2.37 (-2.63 to -2.1)
Tropical Latin America	67643 (64707-70678)	142.68 (136.43-149.01)	136260 (127556-145061)	111.39 (104.13-118.55)	-0.83 (-0.94 to -0.72)
Central Latin America	33053 (31787-34229)	80.58 (77.42-83.57)	46913 (40812-54340)	40.12 (34.98-46.35)	-2.71 (-2.84 to -2.57)
Southern Latin America	37141 (34270-40549)	171.66 (158.48-187.41)	25110 (22542-27865)	64.58 (58.01-71.63)	-3.2 (-3.35 to -3.05)
Caribbean	15997 (14597-17664)	125.66 (114.63-138.76)	32297 (27359-38531)	125.41 (106.33-149.21)	0.14 (0.03 to 0.25)
Central Europe	155826 (147600-165772)	225.47 (213.67-239.95)	126372 (114937-138018)	135.96 (123.6-148.39)	-1.83 (-1.96 to -1.7)
Eastern Europe	289183 (279501-299180)	250.86 (241.88-259.93)	162159 (142446-183809)	114.67 (100.9-130.02)	-3.16 (-3.38 to -2.94)
Central Asia	36725 (35204-38454)	167.23 (160.12-174.8)	24917 (22167-27782)	61.41 (54.76-68.22)	-3.32 (-3.44 to -3.19)
North Africa and Middle East	95449 (78905-113899)	103.47 (85.87-124.75)	159073 (138580-180857)	64.86 (56.79-73.7)	-1.58 (-1.63 to -1.53)
South Asia	501975 (419231-597265)	148.26 (123.66-176.62)	903595 (775597-1052134)	116.02 (99.73-134.81)	-0.94 (-1.03 to -0.85)
Southeast Asia	77969 (67118-88453)	60.48 (51.93-68.43)	168086 (144266-197090)	51.08 (44.2-59.42)	-0.58 (-0.6 to -0.55)
East Asia	310167 (247387-372880)	68.09 (54.78-81.37)	429393 (318532-556282)	39.46 (29.46-50.77)	-1.79 (-1.9 to -1.69)
Oceania	380 (272-511)	24.23 (17.75-32.48)	836 (616-1140)	20.87 (15.73-28.5)	-0.54 (-0.58 to -0.49)
Western Sub-Saharan Africa	28263 (22006-35491)	57.54 (45.33-71.62)	57951 (45058-71238)	56.75 (45.29-69.3)	0.09 (0.02 to 0.15)
Eastern Sub-Saharan Africa	31553 (25463-38977)	76.13 (61.48-93.7)	52558 (39493-69401)	56.55 (42.89-73.38)	-1.16 (-1.23 to -1.1)
Central Sub-Saharan Africa	9656 (6691-13037)	82.7 (58.75-110.22)	19884 (14299-26379)	68.97 (50.47-90.48)	-0.63 (-0.74 to -0.52)
Southern Sub-Saharan Africa	13831 (11291-18066)	105.27 (85.67-138.41)	25833 (21958-30089)	93.24 (80-107.45)	-0.68 (-1.05 to -0.32)

During this period, the global incidence of LC cases grew from 109,878 to 171,788, while the age-standardized incidence rate (ASIR) fell from 5.8 to 4.16 (EAPC = -1.21, 95% CI: [-1.29, -1.14]) (**Table 3**). In regions with middle SDI, the incidence of LC rose rapidly, surpassing middle-high SDI regions in 2021, leading in total incident cases. Meanwhile, regions with middle-high SDI continued to report the highest ASIR for LC. In different regions, South Asia reported the highest number of cases in 2021 (39,013, 95% UI: 33,487–45,232), while Central Europe had the highest ASIR at 8.62 (95% UI: 7.83–9.42). At the national level, China contributed the largest number of new LC cases in 2021 (32,510, 95% UI: 24,068–42,390), followed by India (30,029, 95% UI: 25,462–35,264) and the United States (13,135, 95% UI: 12,472–13,684) (**Figure 4B**). Together, these three nations made up about 44% of the world’s new cases. Monaco had the highest ASIR globally (**Supplementary Figure S3B**).

Over the past 32 years, deaths related to laryngeal cancer have increased from 75,262 to 100,393, while the age-standardized death rate (ASDR) dropped from 4.12 to 2.49 (EAPC = -1.79, 95% CI: [-1.87, -1.71]) (**Table 3**). The mortality rate has shown a declining trend across all SDI areas, with middle SDI areas reporting the greatest number of deaths in 2021, while low-middle SDI areas reported the greatest ASDR in 2021. In different regions, South Asia had the greatest number of LC deaths (31,963, 95% UI: 27,536–36,964) and ASDR (4.43, 95% UI: 3.84–5.10). At the country level, India reported the greatest laryngeal cancer mortality (24,315, 95% UI: 20,675–28,494), followed by China (16,461, 95% UI: 12,221–21,222) and Brazil (4,768, 95% UI: 4,534–5,165) (**Figure 4C**). Cuba had the greatest ASDR worldwide (**Supplementary Figure S3C**).

LC DALYs showed a similar pattern, steadily rising from 1990 to 2021, while the age-standardized disability-adjusted life year rate (ASDALYR) declined (EAPC = -1.93, 95% CI: [-1.87, -1.71]) (**Table 3**). In 2021, the global total of LC DALYs totaled 2.69 million, with an ASDALYR of 64.13 (95% UI: 59.35–69.57). Middle SDI areas accounted for the greatest number of DALYs in 2021, with 821,659 (95% UI: 727,232–923,094) DALYs, while low-middle SDI areas reported the greatest ASDALYR at 98.61 (95%UI: 87.50–111.37). Regionally, South Asia reported the greatest number of disability-adjusted life years (903,594, 95%UI: 775,597–1,052,134), while Central Europe recorded the greatest ASDALYR at 135.96 (95% UI: 123.60–148.39). At the country level, India reported the greatest laryngeal cancer DALYs in 2021 (684,562, 95% UI: 578,937–805,391), followed by China (416,766, 95%UI: 304,820–544,630) and Pakistan (138,521, 95%UI: 96,382–192,188) (**Figure 4D**), accounting for approximately 46% of the global total. Montenegro reported the greatest ASDALYR globally (**Supplementary Figure S3D**).

### Other pharyngeal cancer

**Figure 5:**
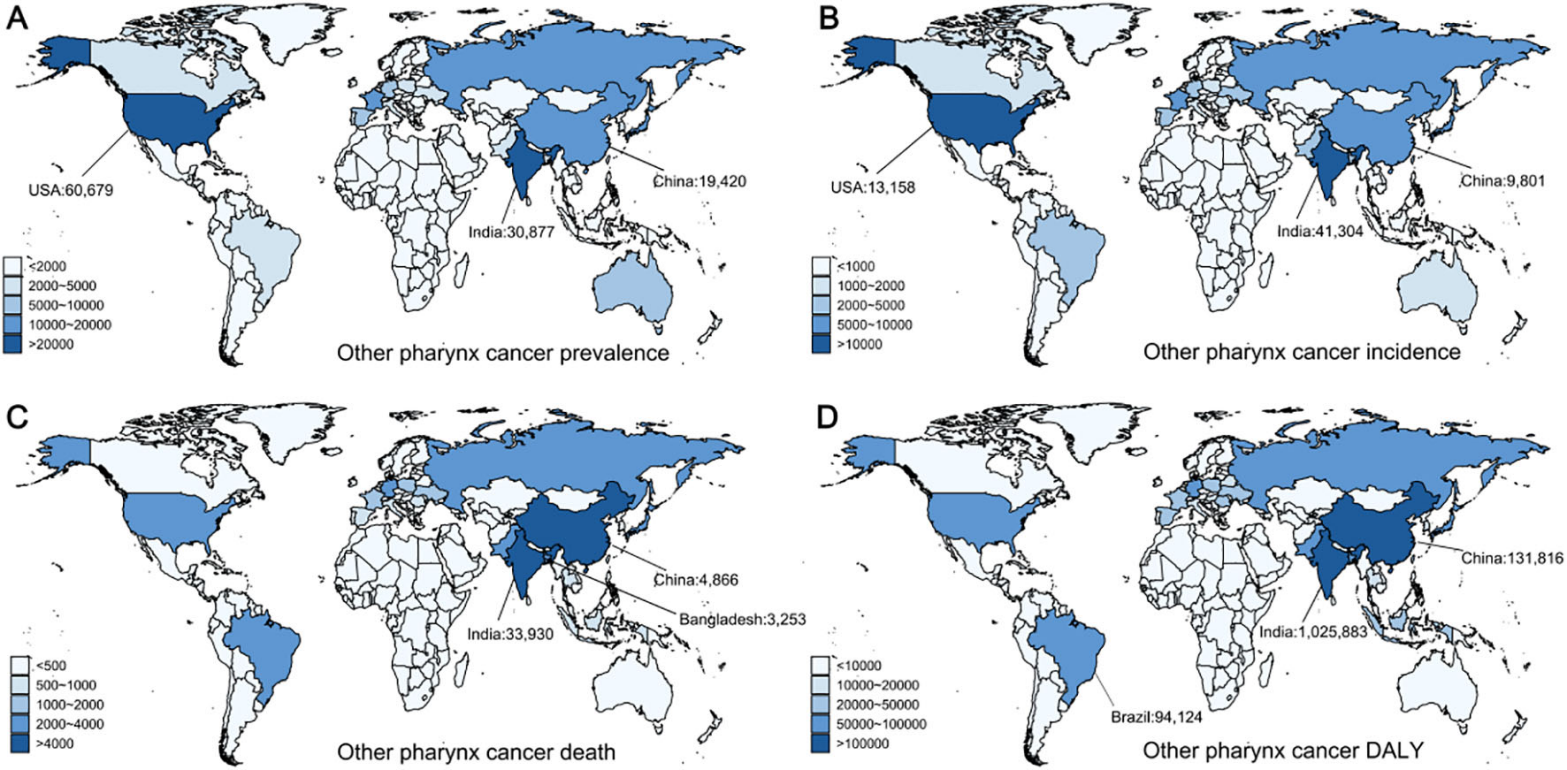
Global distribution of other pharyngeal cancer prevalence, incidence, deaths, and disability-adjusted life years (DALYs) in 2021. **(A)** Prevalence of other pharyngeal cancer. **(B)** Incidence of other pharyngeal cancer. **(C)** Mortality from other pharyngeal cancer. **(D)** DALYs from other pharyngeal cancer.

Other pharyngeal cancer is the fastest-growing type among the four male head and neck cancers. Global prevalence cases increased from 73,250 in 1990 to 258,722 in 2021. During this period, the ASPR increased from 3.57 to 6.05 (EAPC = 1.78, 95% CI: [1.7, 1.87]) (**Table 4**). High SDI regions reported the greatest number of cases and the greatest ASPR, with the rate continuing to rise significantly (EAPC = 2.13, 95% CI: [1.98, 2.28]). Regionally, high-income North America recorded the greatest number of cases in 2021, with a total of 64,680 (95% UI: 62,223–67,094), whereas Australasia had the greatest ASPR at 29.03 (95% UI: 24.63–33.83). Globally, the United States reported the greatest number of new cases in 2021 (60,679, 95% UI: 58,437–63,056), with India (30,877, 95% UI: 26,118–35,913) and China (19,420, 95% UI: 14,451–25,821) ranking second and third, respectively (**Figure 5A**). Together, these nations represented approximately 43% of the global new cases. Portugal had the highest ASPR (**Supplementary Figure S4A**).

**Table 4:** Global and regional trends in other pharynx cancer burden: Prevalence, incidence, mortality, and disability-adjusted life years (1990-2021).

Location	1990 prevalence cases (95% UI)	1990 ASPR (95% UI)	2021 prevalence cases (95% UI)	2021 ASPR (95% UI)	1990–2021 EAPC (95% CI)
Global	73251 (70410-76388)	3.57 (3.43-3.73)	258723 (247045-272324)	6.05 (5.78-6.36)	1.78 (1.7 to 1.87)
High SDI	45269 (43830-46752)	9.33 (9.02-9.63)	150139 (143648-157182)	17.2 (16.47-18)	2.13 (1.98 to 2.28)
High-middle SDI	14237 (13622-15032)	2.88 (2.76-3.05)	52782 (48280-57913)	5.6 (5.13-6.15)	2.09 (1.87 to 2.31)
Middle SDI	6191 (5610-6776)	1.09 (0.99-1.19)	29732 (26245-33075)	2.11 (1.87-2.35)	1.99 (1.74 to 2.24)
Low-middle SDI	6118 (4897-7655)	1.76 (1.41-2.2)	21716 (18549-25093)	2.85 (2.44-3.3)	1.66 (1.54 to 1.77)
Low SDI	1370 (1031-1869)	1.06 (0.79-1.43)	4140 (3294-5336)	1.49 (1.18-1.91)	1.16 (1.01 to 1.31)
High-income Asia Pacific	3176 (2940-3397)	3.22 (2.99-3.44)	22888 (21008-24838)	12.68 (11.63-13.73)	4.47 (3.88 to 5.05)
High-income North America	23335 (22778-23915)	16.04 (15.64-16.44)	64680 (62223-67094)	22.76 (21.92-23.61)	1.35 (1.25 to 1.46)
Western Europe	19610 (18337-20863)	8.42 (7.88-8.98)	59510 (54572-65379)	16.92 (15.52-18.53)	2.09 (1.76 to 2.43)
Australasia	1743 (1549-1938)	15.99 (14.24-17.77)	6547 (5545-7602)	29.03 (24.63-33.83)	1.97 (1.67 to 2.28)
Andean Latin America	35 (31-42)	0.34 (0.3-0.4)	106 (82-134)	0.36 (0.28-0.45)	0.07 (-0.54 to 0.69)
Tropical Latin America	1111 (1050-1174)	2.31 (2.19-2.45)	4029 (3742-4314)	3.24 (3.01-3.47)	0.93 (0.77 to 1.09)
Central Latin America	188 (181-197)	0.45 (0.43-0.47)	660 (578-758)	0.55 (0.48-0.63)	0.46 (0.28 to 0.64)
Southern Latin America	412 (365-461)	1.9 (1.68-2.12)	566 (479-662)	1.47 (1.24-1.72)	-0.57 (-0.91 to -0.22)
Caribbean	285 (263-308)	2.22 (2.05-2.41)	732 (625-867)	2.82 (2.41-3.33)	1.05 (0.64 to 1.47)
Central Europe	2328 (2208-2464)	3.38 (3.22-3.57)	8703 (7803-9658)	9.93 (8.89-11.03)	3.46 (3.24 to 3.69)
Eastern Europe	6597 (6258-7117)	5.5 (5.22-5.95)	21939 (19399-24250)	15.89 (14.06-17.55)	3.67 (3.05 to 4.29)
Central Asia	250 (226-280)	1.15 (1.04-1.29)	356 (310-408)	0.87 (0.77-0.99)	-0.96 (-1.17 to -0.74)
North Africa and Middle East	234 (196-274)	0.25 (0.21-0.29)	1232 (1030-1514)	0.45 (0.38-0.55)	2.08 (1.95 to 2.22)
South Asia	9065 (7498-11125)	2.62 (2.16-3.21)	36385 (31056-41783)	4.56 (3.9-5.22)	1.79 (1.65 to 1.93)
Southeast Asia	1073 (876-1308)	0.82 (0.67-0.99)	5110 (4130-6179)	1.46 (1.19-1.75)	1.84 (1.65 to 2.04)
East Asia	3363 (2703-4159)	0.72 (0.59-0.89)	24047 (18963-30440)	2.14 (1.69-2.7)	3.77 (3.32 to 4.22)
Oceania	2 (2-3)	0.13 (0.1-0.19)	6 (5-8)	0.14 (0.11-0.18)	0.37 (0.23 to 0.51)
Western Sub-Saharan Africa	92 (70-116)	0.17 (0.13-0.22)	242 (185-314)	0.21 (0.16-0.27)	0.61 (0.46 to 0.76)
Eastern Sub-Saharan Africa	229 (176-293)	0.55 (0.42-0.7)	605 (425-882)	0.62 (0.44-0.9)	0.32 (0.26 to 0.38)
Central Sub-Saharan Africa	32 (24-41)	0.27 (0.2-0.35)	89 (63-125)	0.29 (0.21-0.42)	0.34 (0.07 to 0.61)
Southern Sub-Saharan Africa	90 (73-115)	0.67 (0.53-0.86)	290 (243-344)	1.01 (0.85-1.18)	1.35 (1.21 to 1.48)
	1990 incidence cases (95% UI)	1990 ASIR (95% UI)	2021 incidence cases (95% UI)	2021 ASIR (95% UI)	1990–2021 EAPC (95% CI)
Global	52517 (49128-56661)	2.64 (2.47-2.85)	137066 (128148-146458)	3.26 (3.05-3.48)	0.64 (0.56 to 0.71)
High SDI	18697 (17979-19454)	3.87 (3.73-4.03)	44967 (42602-47173)	4.99 (4.74-5.23)	0.87 (0.8 to 0.94)
High-middle SDI	12324 (11644-13136)	2.58 (2.44-2.75)	25485 (23267-27774)	2.71 (2.48-2.95)	-0.05 (-0.2 to 0.1)
Middle SDI	9226 (8334-10119)	1.68 (1.52-1.84)	29328 (26084-32575)	2.15 (1.91-2.38)	0.6 (0.45 to 0.76)
Low-middle SDI	9942 (7940-12449)	2.91 (2.32-3.65)	30912 (26352-35679)	4.17 (3.56-4.79)	1.26 (1.19 to 1.34)
Low SDI	2262 (1696-3086)	1.77 (1.33-2.41)	6233 (4965-8006)	2.31 (1.83-2.95)	0.95 (0.82 to 1.07)
High-income Asia Pacific	1541 (1470-1603)	1.64 (1.57-1.71)	8462 (7805-9052)	4.3 (4-4.6)	3.09 (2.66 to 3.52)
High-income North America	6036 (5879-6183)	4.11 (4-4.21)	14300 (13741-14819)	4.93 (4.74-5.11)	0.8 (0.63 to 0.97)
Western Europe	11495 (10847-12204)	4.86 (4.59-5.15)	19923 (18225-21725)	5.37 (4.92-5.86)	0.13 (-0.03 to 0.29)
Australasia	503 (453-555)	4.63 (4.18-5.11)	1388 (1180-1608)	5.99 (5.08-6.96)	0.81 (0.45 to 1.18)
Andean Latin America	56 (49-66)	0.56 (0.48-0.65)	119 (92-149)	0.42 (0.32-0.52)	-1.03 (-1.55 to -0.5)
Tropical Latin America	1593 (1508-1681)	3.42 (3.24-3.61)	4433 (4118-4730)	3.63 (3.37-3.88)	0.07 (-0.09 to 0.24)
Central Latin America	283 (271-296)	0.7 (0.67-0.73)	750 (658-856)	0.64 (0.57-0.73)	-0.46 (-0.64 to -0.28)
Southern Latin America	501 (446-560)	2.34 (2.08-2.62)	427 (369-495)	1.11 (0.95-1.28)	-2.09 (-2.43 to -1.75)
Caribbean	356 (328-386)	2.84 (2.61-3.07)	677 (576-789)	2.64 (2.24-3.07)	0.05 (-0.37 to 0.47)
Central Europe	2642 (2505-2793)	3.85 (3.66-4.06)	6254 (5613-6899)	6.88 (6.17-7.58)	1.85 (1.71 to 1.99)
Eastern Europe	5248 (4910-5767)	4.52 (4.24-4.96)	10537 (9365-11751)	7.47 (6.66-8.33)	1.5 (1.25 to 1.76)
Central Asia	358 (323-400)	1.71 (1.54-1.91)	458 (402-523)	1.18 (1.04-1.33)	-1.27 (-1.47 to -1.07)
North Africa and Middle East	339 (282-400)	0.38 (0.31-0.45)	1071 (893-1308)	0.43 (0.36-0.53)	0.52 (0.46 to 0.57)
South Asia	14573 (12026-17915)	4.31 (3.54-5.29)	49124 (42000-56353)	6.32 (5.41-7.24)	1.26 (1.18 to 1.34)
Southeast Asia	1628 (1334-1978)	1.28 (1.05-1.55)	5439 (4450-6516)	1.64 (1.35-1.94)	0.72 (0.6 to 0.85)
East Asia	4649 (3710-5796)	1.05 (0.85-1.29)	11897 (9348-15010)	1.09 (0.86-1.37)	-0.14 (-0.54 to 0.27)
Oceania	4 (3-5)	0.21 (0.16-0.3)	9 (7-12)	0.21 (0.17-0.28)	0.27 (0.12 to 0.42)
Western Sub-Saharan Africa	146 (113-185)	0.28 (0.22-0.36)	352 (270-452)	0.31 (0.24-0.4)	0.36 (0.24 to 0.49)
Eastern Sub-Saharan Africa	378 (292-484)	0.92 (0.71-1.17)	919 (646-1337)	0.98 (0.7-1.41)	0.12 (0.08 to 0.16)
Central Sub-Saharan Africa	54 (40-69)	0.47 (0.34-0.59)	139 (98-196)	0.48 (0.34-0.69)	0.19 (-0.05 to 0.43)
Southern Sub-Saharan Africa	133 (107-170)	1.01 (0.81-1.31)	386 (325-456)	1.4 (1.19-1.64)	1.05 (0.82 to 1.28)
	1990 death cases (95% UI)	1990 ASDR (95% UI)	2021 death cases (95% UI)	2021 ASDR (95% UI)	1990–2021 EAPC (95% CI)
Global	36171 (33086-39869)	1.87 (1.71-2.05)	80437 (73959-87131)	1.94 (1.79-2.1)	0.05 (-0.04 to 0.13)
High SDI	8438 (8097-8817)	1.77 (1.7-1.85)	15228 (14311-16071)	1.64 (1.54-1.73)	-0.26 (-0.35 to -0.16)
High-middle SDI	8273 (7749-8893)	1.79 (1.68-1.92)	12480 (11415-13523)	1.34 (1.23-1.45)	-1.2 (-1.33 to -1.07)
Middle SDI	8049 (7285-8819)	1.52 (1.38-1.66)	20893 (18508-23334)	1.57 (1.4-1.75)	-0.09 (-0.2 to 0.02)
Low-middle SDI	9236 (7370-11560)	2.77 (2.21-3.47)	26273 (22421-30220)	3.64 (3.11-4.19)	0.99 (0.94 to 1.04)
Low SDI	2126 (1593-2902)	1.71 (1.28-2.32)	5480 (4355-7013)	2.11 (1.68-2.69)	0.8 (0.69 to 0.9)
High-income Asia Pacific	735 (708-762)	0.82 (0.79-0.85)	3375 (3119-3567)	1.6 (1.48-1.69)	2.09 (1.76 to 2.42)
High-income North America	1612 (1564-1652)	1.09 (1.06-1.12)	3094 (2952-3215)	1.04 (0.99-1.08)	0.02 (-0.26 to 0.3)
Western Europe	6089 (5756-6439)	2.56 (2.43-2.71)	7384 (6718-8119)	1.89 (1.72-2.08)	-1.21 (-1.34 to -1.07)
Australasia	154 (140-169)	1.43 (1.31-1.58)	288 (246-333)	1.19 (1.02-1.37)	-0.72 (-1.13 to -0.3)
Andean Latin America	53 (46-61)	0.53 (0.47-0.62)	92 (72-116)	0.33 (0.26-0.41)	-1.64 (-2.13 to -1.16)
Tropical Latin America	1351 (1282-1424)	3 (2.84-3.16)	3242 (3008-3457)	2.7 (2.51-2.89)	-0.42 (-0.58 to -0.26)
Central Latin America	254 (243-266)	0.65 (0.62-0.68)	575 (505-656)	0.5 (0.44-0.57)	-0.99 (-1.18 to -0.81)
Southern Latin America	385 (346-428)	1.83 (1.64-2.04)	264 (227-303)	0.69 (0.59-0.79)	-2.76 (-3.12 to -2.41)
Caribbean	290 (267-316)	2.36 (2.17-2.57)	480 (407-564)	1.89 (1.6-2.22)	-0.43 (-0.85 to -0.02)
Central Europe	1956 (1853-2069)	2.88 (2.73-3.04)	3610 (3255-3988)	3.91 (3.52-4.32)	0.97 (0.88 to 1.06)
Eastern Europe	3275 (3048-3625)	2.93 (2.73-3.23)	5046 (4457-5753)	3.57 (3.15-4.06)	0.42 (0.26 to 0.58)
Central Asia	303 (273-338)	1.5 (1.35-1.69)	363 (317-417)	0.97 (0.85-1.11)	-1.48 (-1.69 to -1.27)
North Africa and Middle East	294 (244-350)	0.34 (0.29-0.41)	717 (597-875)	0.31 (0.26-0.38)	-0.29 (-0.34 to -0.25)
South Asia	13408 (11020-16518)	4.07 (3.33-5.03)	40586 (34660-46479)	5.35 (4.59-6.12)	0.92 (0.87 to 0.97)
Southeast Asia	1437 (1180-1744)	1.17 (0.96-1.4)	3948 (3274-4704)	1.25 (1.05-1.47)	0.11 (0.02 to 0.2)
East Asia	3916 (3118-4886)	0.94 (0.76-1.15)	5817 (4596-7271)	0.56 (0.45-0.7)	-2.11 (-2.5 to -1.73)
Oceania	3 (2-5)	0.2 (0.15-0.28)	8 (6-11)	0.2 (0.15-0.26)	0.2 (0.06 to 0.35)
Western Sub-Saharan Africa	135 (104-170)	0.27 (0.21-0.34)	303 (237-387)	0.28 (0.22-0.35)	0.19 (0.08 to 0.3)
Eastern Sub-Saharan Africa	355 (275-455)	0.88 (0.69-1.12)	810 (567-1182)	0.9 (0.64-1.29)	-0.03 (-0.06 to 0)
Central Sub-Saharan Africa	51 (38-66)	0.46 (0.34-0.59)	125 (87-179)	0.45 (0.32-0.66)	0.08 (-0.14 to 0.3)
Southern Sub-Saharan Africa	114 (92-147)	0.9 (0.72-1.17)	310 (262-365)	1.17 (1-1.36)	0.84 (0.56 to 1.12)
	1990 DALY cases (95% UI)	1990 ASDALYR (95% UI)	2021 DALY cases (95% UI)	2021ASDALYR (95% UI)	1990–2021 EAPC (95% CI)
Global	1129299 (1033227-1247782)	54.85 (50.2-60.59)	2333897 (2128450-2539518)	54.77 (50-59.57)	-0.11 (-0.19 to -0.03)
High SDI	255095 (243951-267352)	52.73 (50.41-55.29)	389579 (366155-413104)	43.98 (41.39-46.63)	-0.64 (-0.72 to -0.55)
High-middle SDI	259473 (243423-278326)	53.12 (49.79-56.97)	363067 (332010-393250)	38.59 (35.32-41.77)	-1.34 (-1.47 to -1.21)
Middle SDI	253050 (229445-277618)	43.18 (39.07-47.2)	616853 (545011-688151)	44.09 (39.04-49.07)	-0.14 (-0.25 to -0.03)
Low-middle SDI	292738 (234521-366165)	81.58 (65.26-102.11)	794586 (676662-919383)	103.01 (87.91-118.81)	0.86 (0.81 to 0.91)
Low SDI	67427 (50446-92180)	50.21 (37.55-68.56)	167455 (133061-215414)	58.33 (46.32-74.75)	0.55 (0.46 to 0.64)
High-income Asia Pacific	20477 (19720-21264)	21.41 (20.65-22.21)	70086 (65280-74211)	36.42 (33.99-38.63)	1.62 (1.25 to 1.99)
High-income North America	46234 (44864-47665)	31.57 (30.62-32.57)	82975 (79444-86523)	28.85 (27.64-30.08)	-0.08 (-0.33 to 0.18)
Western Europe	187512 (176917-198450)	79.78 (75.32-84.43)	193465 (176185-214598)	52.9 (48.14-58.54)	-1.61 (-1.75 to -1.47)
Australasia	4351 (3950-4803)	40.03 (36.19-44.25)	7661 (6571-8821)	33.38 (28.71-38.3)	-0.63 (-1.03 to -0.22)
Andean Latin America	1486 (1296-1752)	13.81 (12.06-16.2)	2398 (1881-3059)	8.21 (6.47-10.47)	-1.81 (-2.3 to -1.32)
Tropical Latin America	42477 (40275-44746)	86.8 (82.32-91.41)	95698 (88783-102413)	77.2 (71.65-82.54)	-0.51 (-0.7 to -0.33)
Central Latin America	6966 (6668-7310)	16.26 (15.57-17.04)	14853 (13013-17091)	12.46 (10.93-14.32)	-1.02 (-1.21 to -0.83)
Southern Latin America	11351 (10070-12743)	52.17 (46.29-58.58)	7145 (6124-8325)	18.49 (15.81-21.6)	-2.99 (-3.34 to -2.63)
Caribbean	7813 (7148-8572)	60.82 (55.63-66.75)	12967 (10881-15449)	50.19 (42.17-59.66)	-0.34 (-0.75 to 0.07)
Central Europe	63310 (60016-66988)	91.42 (86.82-96.54)	104928 (93755-116151)	117.19 (104.83-129.58)	0.72 (0.59 to 0.85)
Eastern Europe	105626 (98241-117224)	89.79 (83.7-99.67)	154223 (135560-176945)	109.15 (95.94-124.99)	0.35 (0.18 to 0.52)
Central Asia	9474 (8549-10586)	42.7 (38.62-47.64)	10797 (9382-12473)	26.1 (22.87-29.97)	-1.74 (-1.95 to -1.52)
North Africa and Middle East	8812 (7392-10465)	9.15 (7.63-10.87)	20925 (17274-25854)	8.01 (6.64-9.82)	-0.38 (-0.43 to -0.34)
South Asia	429706 (354029-531512)	120.45 (99.02-148.55)	1226521 (1046018-1407575)	152.46 (130.08-174.74)	0.79 (0.74 to 0.84)
Southeast Asia	43707 (35912-53082)	32.12 (26.42-38.93)	117291 (96567-141467)	33.82 (28.1-40.49)	0.07 (-0.03 to 0.18)
East Asia	118742 (94078-148674)	24.83 (19.82-31.03)	160699 (125795-203152)	14.66 (11.54-18.39)	-2.16 (-2.55 to -1.76)
Oceania	108 (78-155)	5.66 (4.18-8.03)	268 (202-364)	5.56 (4.23-7.47)	0.15 (0.01 to 0.3)
Western Sub-Saharan Africa	4500 (3453-5752)	8.27 (6.36-10.51)	10347 (7951-13358)	8.62 (6.71-11.04)	0.17 (0.06 to 0.29)
Eastern Sub-Saharan Africa	11290 (8714-14566)	26.01 (20.12-33.36)	26643 (18541-39302)	26.51 (18.52-38.74)	-0.03 (-0.06 to 0)
Central Sub-Saharan Africa	1620 (1176-2087)	13 (9.55-16.61)	4069 (2825-5787)	12.64 (8.85-18.1)	0.02 (-0.2 to 0.24)
Southern Sub-Saharan Africa	3737 (3027-4736)	26.96 (21.75-34.58)	9937 (8305-11840)	34.08 (28.65-40.18)	0.75 (0.46 to 1.03)

Additionally, the incidence of other pharyngeal cancer increased from 52,517 in 1990 to 137,066 in 2021. During this period, the ASIR rose from 2.64 to 3.26 (EAPC = 0.64, 95% CI: [0.56, 0.71]) (**Table 4**). The highest ASIR and the greatest number of cases were reported by High SDI areas, with the rate continuing to rise significantly (EAPC = 0.87, 95% CI: [0.8, 0.94]). Regionally, Western Europe recorded the most cases in 2021, with a total of 19,923, while Australasia had the greatest ASIR at 5.99 (95% UI: 5.08–6.96). At the country level, India reported the greatest number of new cases in 2021 (41,304, 95% UI: 34,958–47,944), followed by the United States (13,158, 95% UI: 13,741–14,818) and China (9,801, 95% UI: 7,292–12,916) (**Figure 5B**). Together, these three countries made up about 47% of the global new cases. The greatest ASIR was observed in Belarus (**Supplementary Figure S4B**).

From 1990 to 2021, the global number of deaths from other pharyngeal cancer increased from 36,170 to 80,436, with the ASDR rising from 1.87 to 1.94 (EAPC = 0.05, 95% CI: [-0.04, 0.13]) (**Table 4**). Mortality rates increased across all SDI areas, with the greatest number of deaths reported in Low-middle SDI areas (26,273, 95% UI: 22,421–30,220), where the ASDR was also the greatest. In 2021, the greatest number of deaths was reported by South Asia, totaling 40,586 (95% UI: 34,660–46,479), with an ASDR of 5.35 (95% UI: 4.59–6.12). At the country level, India reported the greatest number of deaths in 2021 (33,930, 95% UI: 28,707–39,300), followed by China (4,866, 95% UI: 3,650–6,347) and Bangladesh (3,253, 95% UI: 1,905–5,368) (**Figure 5C**). The aforementioned countries made up about 52% of global deaths. Belarus had the greatest ASDR globally (**Supplementary Figure S4C**).

Over the past three decades, the DALYs for other pharyngeal cancer rose from 1.12 million in 1990 to 2.33 million in 2021 (**Table 4**). Accordingly, the ASDALYR dropped from 54.85 to 54.77 (EAPC = -0.11, 95% CI: [-0.19, -0.03]). The Low-middle SDI regions recorded the greatest DALY and ASDALYR. In 2021, South Asia reported the greatest number of disability-adjusted life years among all regions, totaling 1,226,521 (95% UI: 1,046,018–1,407,575), with the greatest ASDALYR. At the country level, India reported the greatest DALY in 2021 (1,025,883, 95% UI: 866,643–1,193,706), followed by China (131,816, 95% UI: 98,012–174,031) and Brazil (94,124, 95% UI: 87,238–100,802) (**Figure 5D**), together accounting for approximately 51% of the global total. Similarly, Belarus had the greatest ASDALYR globally (**Supplementary Figure S4D**).

### SDI and male head and neck cancers

We use scatter plots to display the dynamic relationship between SDI and age-standardized rates (ASPR, ASIR, ASDR, and ASDALYR) across 21 global regions over the past 30 years, in order to analyze the impact of socioeconomic development (measured by SDI) on the trends of the four types of male head and neck cancers. In most regions, SDI is positively correlated with the ASPR of nasopharyngeal cancer and other pharyngeal cancers, both showing an upward trend as SDI rises.

**Figure 6:**
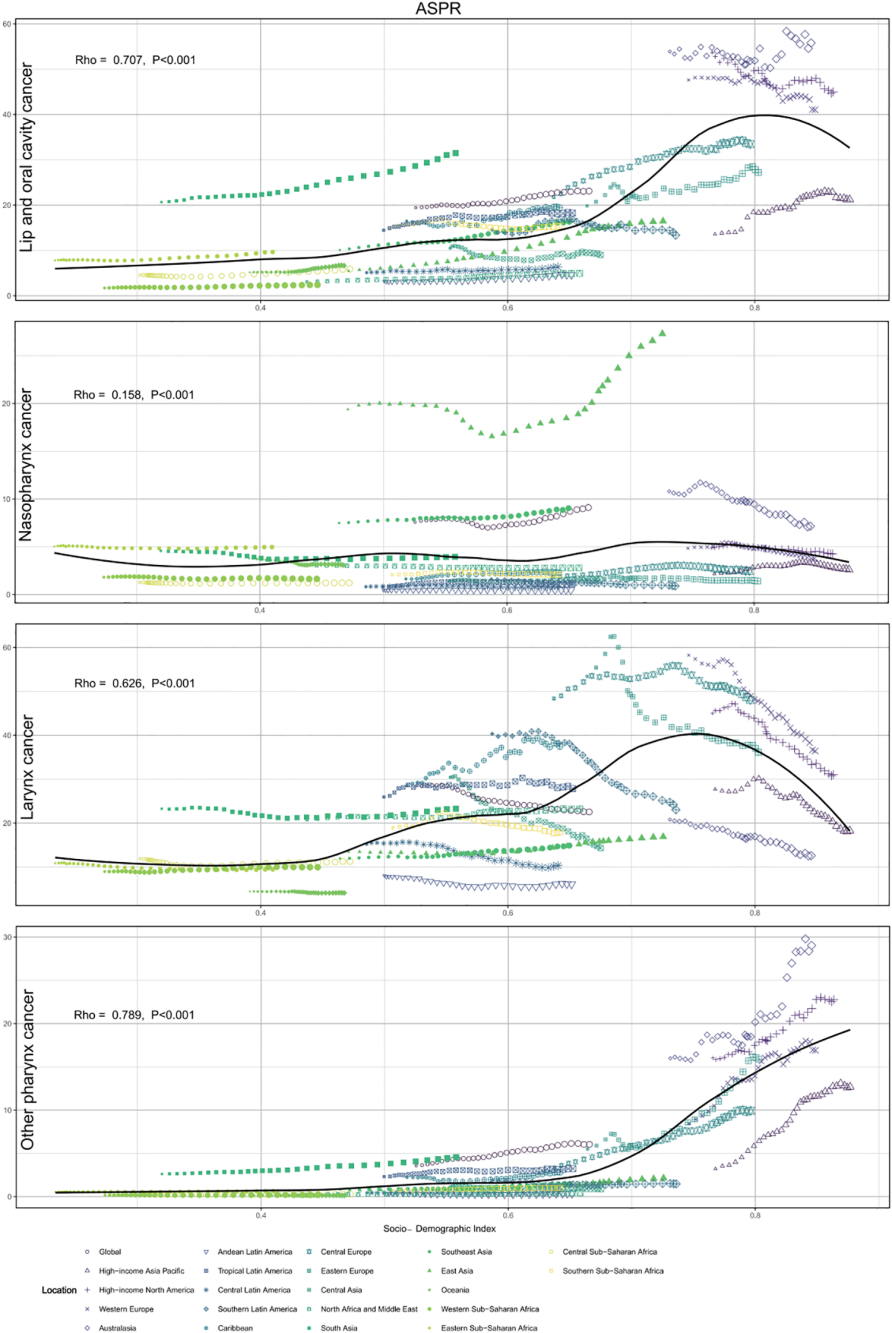
Global trends in age-standardized prevalence rates (ASPRs) and socio-demographic index (SDI) from 1990 to 2021. The Rho values (Spearman’s rho correlation coefficient) indicate the strength of the correlation between the SDI and ASPRs.

The relationship between SDI and oral and lip cancer is not linear but follows an inverted U-shape. Below an SDI of 0.8, higher SDI is typically associated with higher ASPR, whereas above this threshold, the relationship reverses, with further increases in SDI linked to a decline in ASPR. A similar trend is observed in laryngeal cancer, where in regions such as Western Europe, Central Europe, and Eastern Europe, an increase in SDI is associated with a decrease in ASPR (**Figure 6**). Additionally, SDI is strongly positively correlated with the ASIR of the other three cancers, except for nasopharyngeal cancer (**Supplementary Figure S5**). Regarding ASDR, SDI is positively correlated with the ASDR of oral and lip cancer and other pharyngeal cancers, but negatively correlated with the ASDR of nasopharyngeal cancer and laryngeal cancer (**Supplementary Figure S6**). For ASDALYR, SDI is negatively correlated with the ASDALYR of nasopharyngeal cancer and laryngeal cancer, but positively correlated with other pharyngeal cancers (**Supplementary Figure S7**).

### Age structure

Given that age is a crucial factor in tumor burden, we examined the age-based distribution of male head and neck cancers globally. For lip and oral cancer, the greatest prevalence was seen in the 55–59 range, with the highest incidence occurring in the 65–69 range. Mortality peaked at 60-64, and DALY was greatest in the 55–59 range (**Supplementary Figure S8**). For nasopharyngeal cancer, the greatest prevalence, incidence, and DALY occurred in the 50–54 range, while mortality peaked in the 55–59 range. Laryngeal cancer showed its greatest prevalence and DALY in the 60–64 range, while incidence and mortality peaked in the 65–69 range. For other pharyngeal cancers, the greatest prevalence and DALY occurred in the 55–59 range, while incidence and mortality peaked in the 60–64 range.

Overall, the ASIR, ASDR, and ASDALYR of most male head and neck cancers show an increasing trend with age (**Supplementary Figure S9**). Notably, the peak ASPR for lip and oral cancer occurs in the 70–74 range, while the peak ASIR is observed in the 90–94 range, and the peak ASDALYR is seen in the 65–69 range. For nasopharyngeal cancer, the peaks for ASPR, ASIR, ASDR, and ASDALYR are observed in the 50-54, 65-69, 85-89, and 55–59 ranges, respectively. For laryngeal cancer, the peak values for ASPR, ASIR, ASDR, and ASDALYR occur in the 70-74, 85-89, 90-94, and 70–74 ranges, respectively. For other pharyngeal cancers, the peak ASPR and ASIR are observed in the 70–74 range, while the peak ASDR is seen in the 85–89 range, and the peak ASDALYR is observed in the 60–64 range. Although the age distribution of cancer varies slightly across different SDI regions, the overall trends are generally consistent across regions (**Supplementary Figures S10**, **S19**).

### Attributable risk factors

This study analyzed the risk factors for male head and neck cancers. The findings indicate that the risk factors for male head and neck cancers are highly concentrated. For lip and oral cancer, the primary risk factors include smoking, high alcohol consumption, and chewing tobacco. Smoking is the most significant cause of death and disability for lip and oral cancer globally and across all SDI regions, accounting for 32.2% and 30.2% of cases, respectively. In East Asia, smoking is responsible for the highest proportion of deaths and DALYs (51.6% and 50.5%), while Sub-Saharan Africa has the lowest (deaths 10.8%, DALYs 10.2%). Alcohol consumption significantly impacts lip and oral cancer, accounting for 26.3% of global deaths and 30.2% of DALYs. Central Europe has the greatest proportion (45.7% and 47.2%), while North Africa and the Middle East have the lowest (5.2%). Chewing tobacco is another major cause of death and disability, with the greatest proportions in South Asia (deaths 29.7%, DALYs 29.4%) and the lowest in Southern South America (deaths 0.3%, DALYs 0.3%) (**Supplementary Figure S20**).

For nasopharyngeal cancer, the key risk factors include smoking, alcohol consumption, and occupational contact with formaldehyde. Smoking accounts for 23.8% of global deaths and 20.9% of DALYs from nasopharyngeal cancer, with the greatest proportions of smoking-related deaths and DALYs in East Asia (33% and 29.7%, respectively), and the lowest in Sub-Saharan Africa (deaths 3.4%, DALYs 3.2%). Alcohol consumption also plays a major role in nasopharyngeal cancer deaths and DALYs, accounting for 26.3% and 30.2% globally, with Central Europe showing the greatest proportions (deaths 45.7%, DALYs 45.8%), while North Africa and the Middle East report the lowest (deaths 5.3%, DALYs 5.9%). Occupational exposure to formaldehyde accounts for 0.8% of global deaths and 1.1% of DALYs, with East Asia reporting the greatest proportions (1% and 1.2%, respectively) (**Supplementary Figure S21**).

For laryngeal cancer, the key risk factors are smoking, heavy alcohol consumption, and occupational exposures to sulfuric acid and asbestos. Smoking accounts for 72.4% of global laryngeal cancer deaths and 71.6% of DALYs, with the greatest proportions of smoking-related deaths and DALYs in East Asia (86.2% and 85.9%, respectively), and the lowest in Sub-Saharan Africa (deaths 36.1%, DALYs 36.6%). Heavy alcohol consumption is responsible for 14% of global deaths and 14.6% of DALYs, with the greatest proportions in Central Europe (deaths 25.5%, DALYs 26.4%), and the lowest in North Africa and the Middle East (deaths 2.3%, DALYs 2.5%). Occupational exposure to sulfuric acid and asbestos accounts for 3.2% and 3.8%, and 3.2% and 2.4% of global deaths and DALYs, respectively (**Supplementary Figure S22**).

For other pharyngeal cancers, the primary risk factors are smoking and high alcohol consumption. Smoking accounts for 41.9% of global deaths and 41.6% of DALYs, with the greatest proportions in East Asia (deaths 57.5%, DALYs 57.6%) and the lowest in Sub-Saharan Africa (deaths 17%, DALYs 17%). High alcohol consumption accounts for 26.5% of global deaths and 27.5% of DALYs, with the greatest proportions in Central Europe (deaths 44.3%, DALYs 45.4%) and the lowest in North Africa and the Middle East (deaths 4.5%, DALYs 5.1%) (**Supplementary Figure S23**).

The four types of male head and neck cancers mentioned above show significant regional differences. Deaths and disabilities caused by smoking are primarily concentrated in East Asia, while those caused by alcohol consumption are more prominent in Central Europe. Additionally, relevant regions should pay attention to occupational exposures and other related factors, and remain vigilant. Specifically, in high-risk regions like East Asia and Central Europe, health interventions should concentrate on reducing smoking, alcohol consumption, and occupational exposures. Furthermore, tailored cancer prevention and control strategies should be developed for these regions, taking into account the differences in risk factors, to effectively decrease the incidence and death rates of the related cancers.

## Discussion

This study examines the epidemiological patterns and associated risk factors for male head and neck cancers, including lip and oral cavity cancer, nasopharyngeal cancer, laryngeal cancer, and other pharyngeal cancers. The results indicate that lip and oral cavity cancer contributes the most to the global disease burden, followed by laryngeal cancer, other pharyngeal cancers, and nasopharyngeal cancer. In the last thirty years, the incidence and mortality rates of these cancers have steadily risen globally. However, when adjusting for demographic shifts and population growth, the ASPR showed different trends. Specifically, the ASPR for lip and oral cavity cancer, nasopharyngeal cancer, and other pharyngeal cancers increased, while the ASPR for laryngeal cancer decreased. Meanwhile, the ASIR for lip and oral cavity cancer and other pharyngeal cancers increased, whereas the ASIR for nasopharyngeal cancer and laryngeal cancer declined. Notably, the ASDR and ASDALYR for lip and oral cavity cancer, nasopharyngeal cancer, and laryngeal cancer generally decreased. Although the ASDR for other pharyngeal cancers increased, the ASDALYR showed a general decline. The ASPR for all four head and neck cancers increased with higher SDI. Finally, we found that the ASIR and ASDR for most male head and neck cancers increased with age, indicating that age is a significant factor in cancer burden. Especially among individuals aged 65 and above, the rates of incidence and mortality for laryngeal cancer, lip and oral cavity cancer, and other types have significantly increased. Additionally, the major attributable risk factors for these cancers were generally consistent, primarily including smoking and alcohol consumption, with slight variations in risk factors between different cancer types. However, significant differences persist across regions with high and low SDI.

These results align with earlier studies, particularly regarding the steady increase in the prevalence and incidence rates of lip and oral cavity cancer globally from 1990 to 2021, while the ASDR and ASDALYR have continued to decline. The increase in the incidence and prevalence of lip and oral cavity cancer is primarily attributed to the widespread adoption and advancement of screening technologies, as well as improved health awareness, which has led to an increase in early diagnosed cases. At the same time, advancements in treatment techniques, including immunotherapy and targeted therapy, and minimally invasive surgery, have shown substantial improvement cure rates, further reducing the ASDR and ASDALYR. These changes clarify the discrepancy between the increasing ASPR and ASIR, and the decreasing ASDR and ASDALYR, emphasizing the beneficial effects of early detection and advancements in treatment.

The incidence of lip and oral cavity cancer is closely related to the SDI. In high-SDI regions such as Australia, North America, and Western Europe, both the ASPR and ASIR substantially exceed the global average. Additionally, both the ASPR and ASIR increase with age. Smoking, alcohol consumption, and chewing tobacco have been identified as the three major Level 4 risk factors for lip and oral cavity cancer. These factors are closely associated with gender, with smoking and alcohol consumption having a more significant impact on men, which is consistent with previous studies indicating that smoking and alcohol consumption are the primary causative factors for lip and oral cavity cancer (11–13). In India, betel nut and chewing tobacco are often used as part of religious and cultural practices, which has led to a high incidence of oral cancer in the country (14), a similar situation is observed in certain regions of China (15).

Compared to lip and oral cavity cancer, the worldwide impact of nasopharyngeal cancer (NPC) is relatively lighter. Although the ASPR of nasopharyngeal cancer continues to rise globally, its ASIR, ASDR and ASDALYR have rapidly decreased. The burden of nasopharyngeal cancer shows a mild relationship with the SDI (Rho = 0.158, P< 0.001), and there are significant regional variations (16).

Notably, in East Asia, the ASPR is nearly three times the global average. The high incidence of nasopharyngeal cancer in this region can be traced back to several factors: as a virus-associated malignancy, NPC is primarily caused by Epstein-Barr virus (EBV) infection (17). In East Asia, particularly in southern China, the higher EBV infection rate increases the risk of nasopharyngeal cancer (18, 19). Additionally, genetic susceptibility is more pronounced in East Asian populations, making them more sensitive to EBV infection (20, 21). Dietary habits also play a significant role, as traditional preserved foods, salted fish, and smoked foods contain carcinogens such as nitrites, which, when consumed over extended periods, could elevate the likelihood of nasopharyngeal cancer (22). Furthermore, occupational exposure to environmental pollutants, such as formaldehyde, and unhealthy lifestyle habits, including smoking and alcohol consumption, also contribute to the increased risk of nasopharyngeal cancer (23), which aligns with the risk factors identified in this study. Finally, the widespread early screening and diagnosis in East Asia may also contribute to the higher reported incidence of nasopharyngeal cancer.

The ASPR, ASIR, ASDR, and ASDALYR for laryngeal cancer have all decreased globally, with the greatest contribution to this trend from regions with a higher SDI. Additionally, the ASIR, ASDR, and ASDALYR in other SDI regions also continue to decrease. While ASPR is generally positively correlated with SDI, in regions where the SDI exceeds 0.74, the ASPR for laryngeal cancer shows a declining trend. Principal risk factors for laryngeal cancer-related mortality include smoking, alcohol consumption, and certain occupational exposures, such as asbestos and chemicals (24, 25). Although the international burden of laryngeal cancer has reduced, it remains among the leading cancers in men, with a high disability rate, particularly in areas with high smoking rates. Effective early diagnosis and screening measures are still limited in many countries. In recent years, advances in treatment, including genetic testing, personalized neoadjuvant chemotherapy, targeted therapy, immunotherapy, and cutting-edge robotic surgery, have contributed to reducing laryngeal cancer mortality and disability rates to some extent (26, 27).

The prevalence and incidence of other pharyngeal cancers have steadily increased globally, especially in regions with higher SDI. In fact, the ASPR and ASIR for other pharyngeal cancers are positively correlated with SDI, and there has been a significant rise in economically developed areas, including Australia, Western Europe, and North America. Use of tobacco and alcohol are key risk factors for the mortality of other pharyngeal cancers (28). Beyond these established risks, prolonged contact with occupational carcinogens, air pollution, and environmental factors, as well as chronic inflammation such as the prolonged irritation of the throat caused by gastroesophageal reflux disease (GERD), also increases the risk of developing this disease (29). Viral infections (such as HPV) and genetic susceptibility are also important contributing factors (30). As these risk factors persist, public health measures should focus on reducing smoking and alcohol consumption, controlling exposure to occupational carcinogens, improving air quality, by encouraging early intervention and regular screening, it is possible to significantly lower the risk of other pharyngeal cancers.

In recent decades, the global challenge posed by head and neck cancers in men has significantly increased, particularly against the backdrop of rising cancer incidence and disease rates due to population aging. As the global population continues to age, such a trend is anticipated to intensify in the near future, presenting unprecedented risks to global health and healthcare frameworks. It is estimated that between 2018 and 2030, the economic losses caused by head and neck cancers worldwide will exceed $500 billion (31), further highlighting the immense pressure on medical resource allocation and treatment strategies. With the outbreak of the COVID-19 pandemic and the intensification of global armed conflicts, the demand for healthcare resources has surged, placing enormous pressure on cancer prevention and treatment (32, 33). In response to this challenge, countries need to take proactive measures, optimize resource allocation, promote the development of early diagnostic technologies, and strengthen research on personalized treatment. These strategies can effectively alleviate the treatment burden of cancer and improve treatment efficiency.

Specifically, in high-burden countries such as China and India, especially in rural and remote areas, early screening for lip, oral, throat, and nasopharyngeal cancers should be promoted. At the same time, health education should be strengthened, particularly in areas related to smoking, alcohol consumption, and unhealthy dietary habits, to help the public recognize the association between these factors and head and neck cancers. Population-dense countries like China and India need to further strengthen tobacco control measures, particularly in rural and remote areas. In addition to intensifying smoking bans in public places, governments should reduce smoking rates through measures such as increasing tobacco taxes and restricting tobacco advertising. India also needs to enhance regulation of betel nut and chewing tobacco, especially in areas where betel nut consumption is prevalent, and raise public awareness of their harms. Given the deficiencies in head and neck cancer screening in China and India, it is recommended that governments strengthen the research and promotion of early diagnostic technologies, particularly in low-resource areas, by implementing more cost-effective screening methods such as oral and laryngoscopic examinations. Furthermore, through cooperation with international partners, modern screening technologies could be introduced to improve cancer treatment standards.

This study has several limitations. First, although the GBD 2021 data is statistically processed and widely comprehensive, data deficiencies and coefficient differences across regions, particularly in low-income countries, may lead to an underestimation of the actual cancer burden. Second, the lack of statistical data on cancer pathological subtypes has affected the analysis of subtypes. Lastly, the risk factor analysis in the GBD 2021 database is based solely on the available level 4 risk factors, which may introduce bias.

## Conclusion

This study analyzes the global burden of four types of male head and neck cancers: lip and oral cavity cancer, nasopharyngeal cancer, laryngeal cancer, and other pharyngeal cancers. The results indicate that lip and oral cavity cancer bears the greatest global disease burden, while the incidence of nasopharyngeal cancer is on the rise, exhibiting distinct regional characteristics, with a higher prevalence in East and Southeast Asia. The incidence of laryngeal cancer has decreased in most regions, with the most significant decline observed in high-SDI regions. Meanwhile, the incidence of other pharyngeal cancers is increasing.

Key factors such as gender and lifestyle have significantly increased the cancer burden. Addressing these challenges requires the expansion of early prevention and diagnosis, especially in regions with limited medical resources. As the global population ages, there is an urgent need to implement targeted measures for different cancer types and improve cancer care infrastructure. The study emphasizes the importance of formulating public health policies, improving lifestyle habits, and conducting ongoing research.

## Data Availability

The raw data supporting the conclusions of this article will be made available by the authors, without undue reservation.
