# Supplementary material for: Global, regional, and national burden of head and neck cancer in males and associated risk factors from 1990 to 2021: a systematic analysis for the Global Burden of Disease Study 2021
Source: Front Oncol. 2025 Oct 31;15:1607890. doi: 10.3389/fonc.2025.1607890 (PMC12615187; doi:10.3389/fonc.2025.1607890)
Supplement: Supplementary file 1 [file DataSheet1.docx]

**Supplementary information**

***Supplementary Figures***

**Figure S1:** Age-standardized rates of lip and oral cavity cancer in 204 countries or territories in 2021.**A.** Age-standardized prevalence rates of lip and oral cavity cancer; **B.** Age-standardized incidence rates of lip and oral cavity cancer; **C.** Age-standardized death rates of lip and oral cavity cancer; **D.** Age-standardized disability-adjusted life-year rates of lip and oral cavity cancer. Annotations: ASPR, Age-standardized prevalence rate; ASIR, Age-standardized incidence rate; ASDR, Age-standardized death rate; DALY, disability-adjusted life year.

**Figure S2:** Age-standardized rates of nasopharynx cancer in 204 countries or territories in 2021. **A.** Age-standardized prevalence rates of nasopharynx cancer; **B.** Age-standardized incidence rates of nasopharynx cancer; **C.** Age-standardized death rates of nasopharynx cancer; **D.** Age-standardized disability-adjusted life-year rates of nasopharynx cancer. Annotations: ASPR, Age-standardized prevalence rate; ASIR, Age-standardized incidence rate; ASDR, Age-standardized death rate; DALY, disability-adjusted life year.

**Figure S3:** Age-standardized rates of larynx cancer in 204 countries or territories in 2021. **A.** Age-standardized prevalence rates of larynx cancer; **B.** Age-standardized incidence rates of larynx cancer; **C.** Age-standardized death rates of larynx cancer; **D.** Age-standardized disability-adjusted life-year rates of larynx cancer. Annotations: ASPR, Age-standardized prevalence rate; ASIR, Age-standardized incidence rate; ASDR, Age-standardized death rate; DALY, disability-adjusted life year.

**Figure S4:** Age-standardized rates of other pharynx cancer in 204 countries or territories in 2021. **A.** Age-standardized prevalence rates of other pharynx cancer; **B.** Age-standardized incidence rates of other pharynx cancer; **C.** Age-standardized death rates of other pharynx cancer; **D.** Age-standardized disability-adjusted life-year rates of other pharynx cancer. Annotations: ASPR, Age-standardized prevalence rate; ASIR, Age-standardized incidence rate; ASDR, Age-standardized death rate; DALY, disability-adjusted life year.

**Figure S5:** The correlation analyses of age-standardized rates and SDI from 1990 to 2021. The correlations between age standardized ASIR rates and SDI were analyzed based on the data of 21 geographic regions. Annotation: ASIR, Age-standardized incidence rate; Rho. Spearman’s Rho correlation coefficient; SDI, socio-demographic index.

**Figure S6:** The correlation analyses of age-standardized rates and SDI from 1990 to 2021. The correlations between age standardized death rates and SDI were analyzed based on the data of 21 geographic regions. Annotation: ASDR, Age-standardized death rate; Rho. Spearman’s Rho correlation coefficient; SDI, socio-demographic index.

**Figure S7:** The correlation analyses of age-standardized rates and SDI from 1990 to 2021. The correlations between age-standardized DALY rates and SDI were analyzed based on the data of 21 geographic regions. Annotation: ASDALYR, age-standardized DALY rate; DALY, disability adjusted life year; Rho. Spearman’s Rho correlation coefficient; SDI, socio-demographic index.

**Figure S8**: Age distribution of incidence, mortality, and disability-adjusted life years (DALYs) for male head and neck cancers globally in 2021, across five-year age groups, including lip and oral, nasopharyngeal , laryngeal, and other pharyngeal cancers. Annotation: SDI, socio-demographic index.

**Figure S9**: Age distribution of age-standardized rates of incidence, mortality, and DALYs for male head and neck cancers globally in 2021, across five-year age groups, including lip and oral, nasopharyngeal , laryngeal, and other pharyngeal cancers. Annotation: SDI, socio-demographic index.

**Figure S10**: Age distribution of incidence, mortality, and disability-adjusted life years (DALYs) for male head and neck cancers in high SDI regions in 2021, across five-year age groups, including lip and oral, nasopharynx , larynx, and other pharynx cancers. Annotation: SDI, socio-demographic index.

**Figure S11**: Age distribution of age-standardized rates of incidence, mortality, and DALYs for male head and neck cancers in high SDI regions in 2021, across five-year age groups, including lip and oral, nasopharynx , larynx, and other pharynx cancers.Annotation: SDI, socio-demographic index

**Figure S12**: Age distribution of incidence, mortality, and disability-adjusted life years (DALYs) for male head and neck cancers in high-middle SDI regions in 2021, across five-year age groups, including lip and oral, nasopharynx , larynx, and other pharynx cancers. Annotation: SDI, socio-demographic index.

**Figure S13**: Age distribution of age-standardized rates of incidence, mortality, and DALYs for male head and neck cancers in high-middle SDI regions in 2021, across five-year age groups, including lip and oral, nasopharynx , larynx, and other pharynx cancers.Annotation: SDI, socio-demographic index

**Figure S14**: Age distribution of incidence, mortality, and disability-adjusted life years (DALYs) for male head and neck cancers in middle SDI regions in 2021, across five-year age groups, including lip and oral, nasopharynx , larynx, and other pharynx cancers. Annotation: SDI, socio-demographic index.

**Figure S15**: Age distribution of age-standardized rates of incidence, mortality, and DALYs for male head and neck cancers in middle SDI regions in 2021, across five-year age groups, including lip and oral, nasopharynx , larynx, and other pharynx cancers.Annotation: SDI, socio-demographic index

**Figure S16**: Age distribution of incidence, mortality, and disability-adjusted life years (DALYs) for male head and neck cancers in low-middle SDI regions in 2021, across five-year age groups, including lip and oral, nasopharynx , larynx, and other pharynx cancers. Annotation: SDI, socio-demographic index.

**Figure S17**: Age distribution of age-standardized rates of incidence, mortality, and DALYs for male head and neck cancers in low-middle SDI regions in 2021, across five-year age groups, including lip and oral, nasopharynx , larynx, and other pharynx cancers.Annotation: SDI, socio-demographic index

**Figure S18**: Age distribution of incidence, mortality, and disability-adjusted life years (DALYs) for male head and neck cancers in low SDI regions in 2021, across five-year age groups, including lip and oral, nasopharynx , larynx, and other pharynx cancers. Annotation: SDI, socio-demographic index.

**Figure S19**: Age distribution of age-standardized rates of incidence, mortality, and DALYs for male head and neck cancers in low SDI regions in 2021, across five-year age groups, including lip and oral, nasopharynx , larynx, and other pharynx cancers.Annotation: SDI, socio-demographic index

**Figure S20**: Percentage of age-standardized DALYs rates and ASDR of lip and oral cavity cancer attributable to smoking, high alcohol use and chewing tobacco.

**Figure S21**: Percentage of age-standardized DALYs rates and ASDR of nasopharynx cancer attributable to smoking, high alcohol use and occupational exposure to formaldehyde.

**Figure S22**: Percentage of age-standardized DALYs rates and ASDR of larynx cancer attributable to smoking, high alcohol use ,Occupational exposure to sulfuric acid and Occupational exposure to asbestos.

**Figure S23**: Percentage of age-standardized DALYs rates and ASDR of other pharynx cancer attributable to smoking and high alcohol use.

**Figure S1**

**
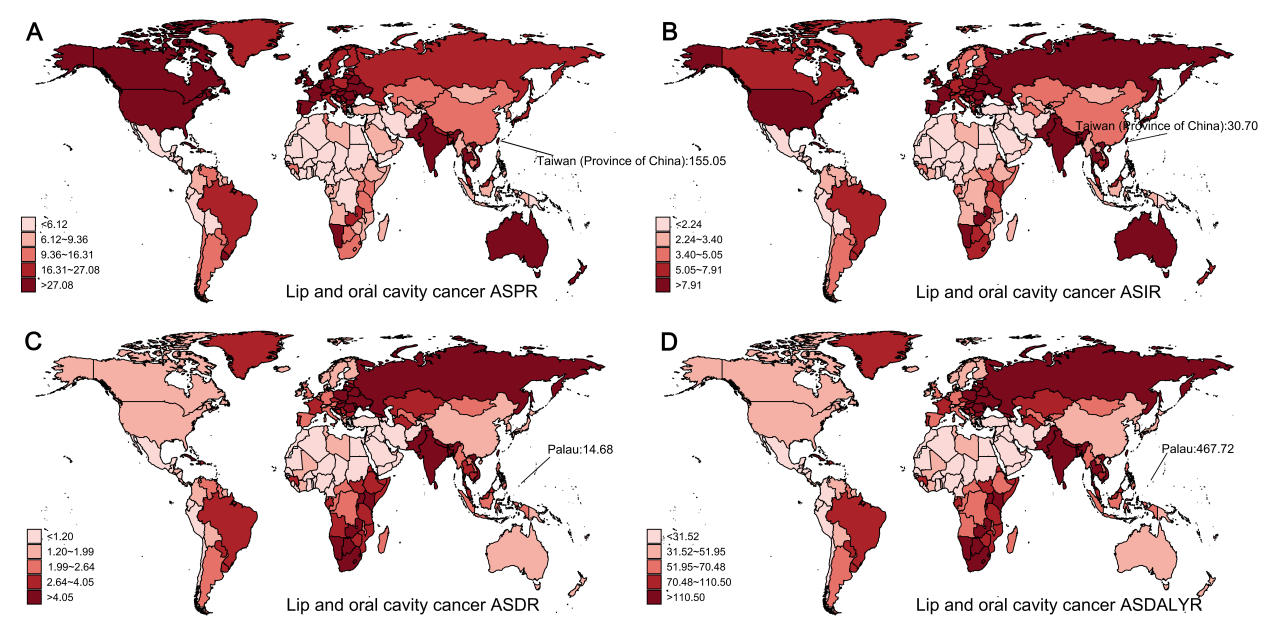
Figure S2**


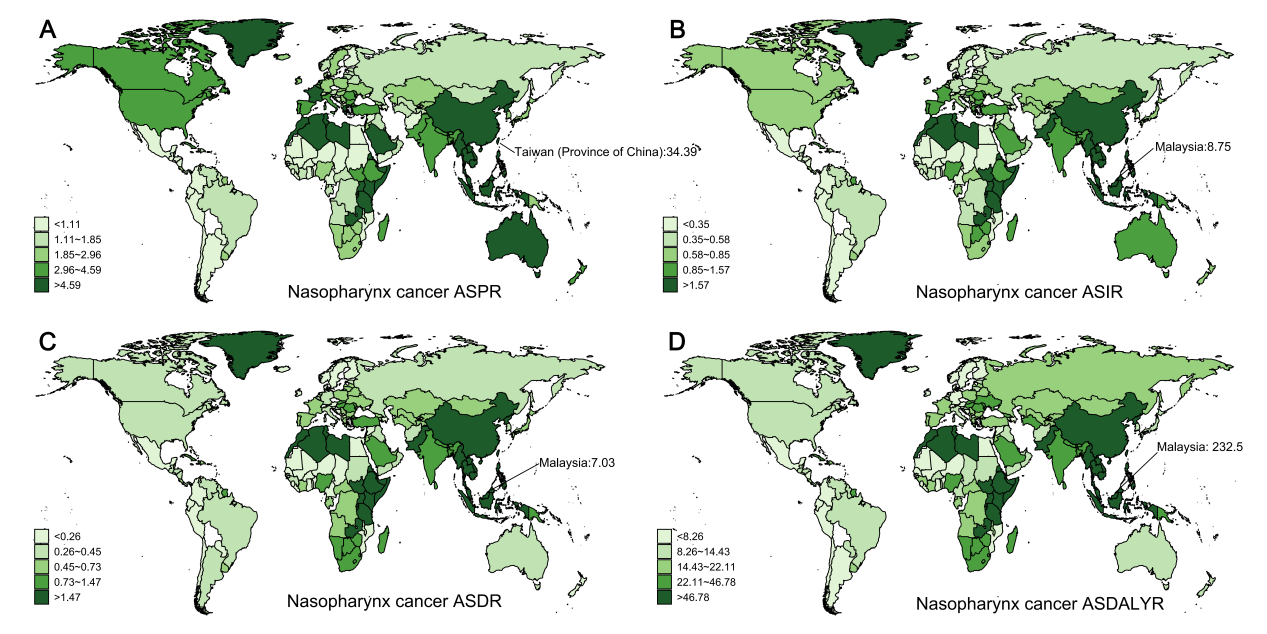


**Figure S3**

**
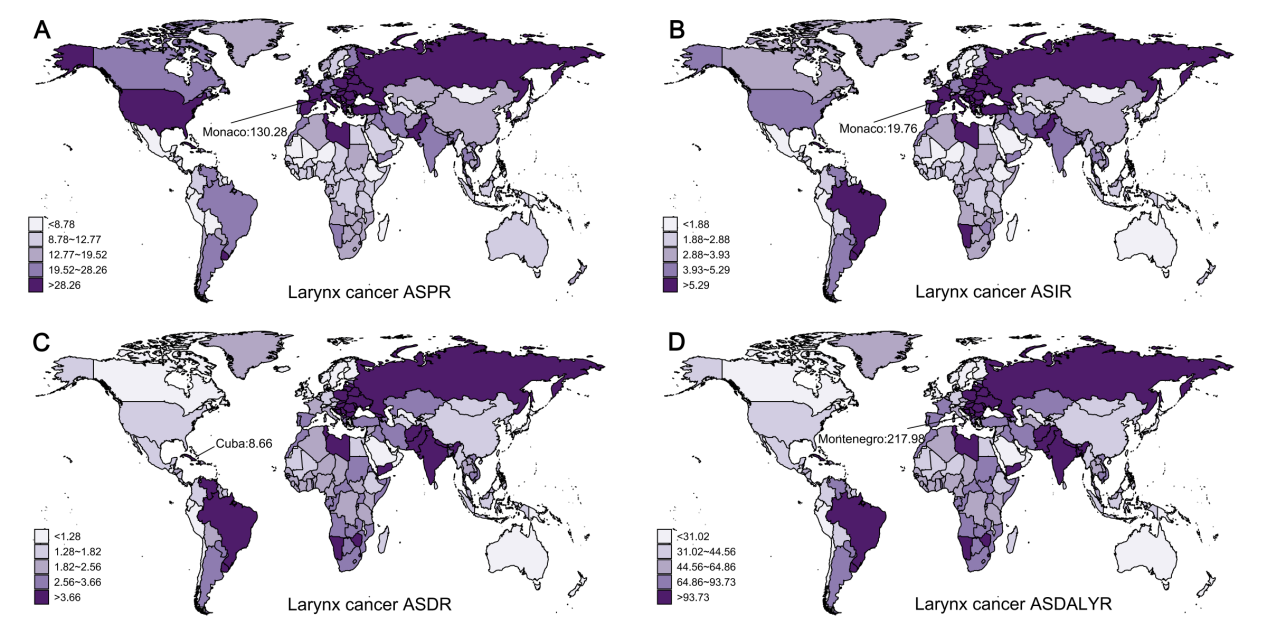
**

**Figure S4**

**
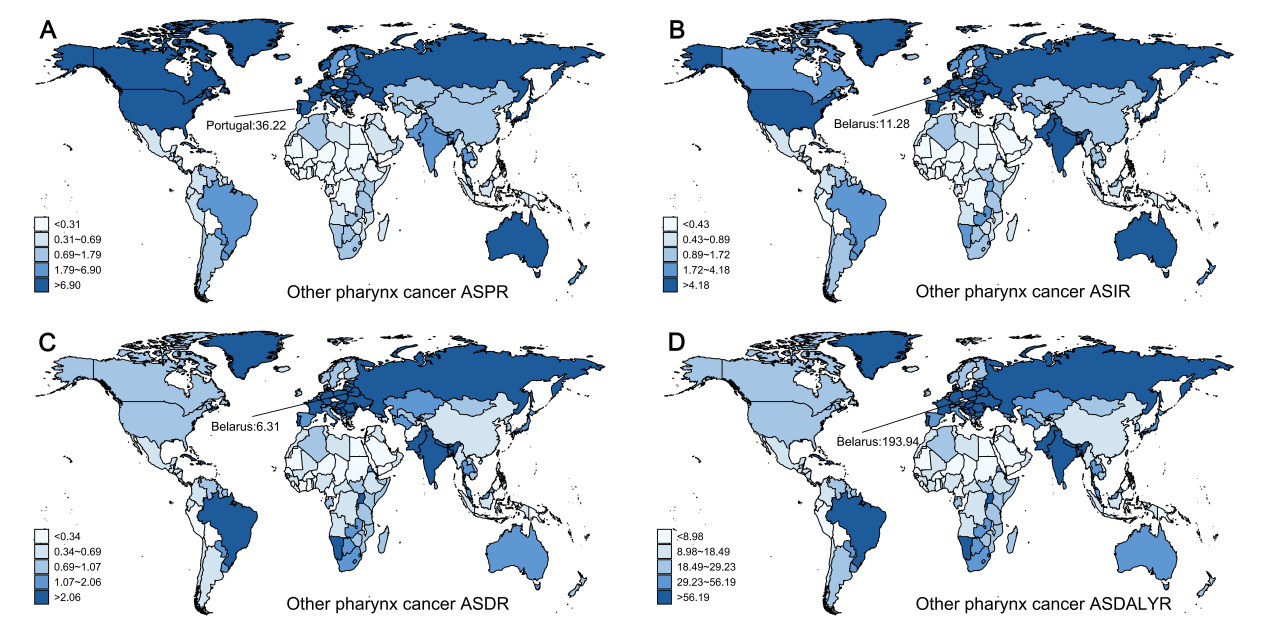
**

**Figure S5**

**
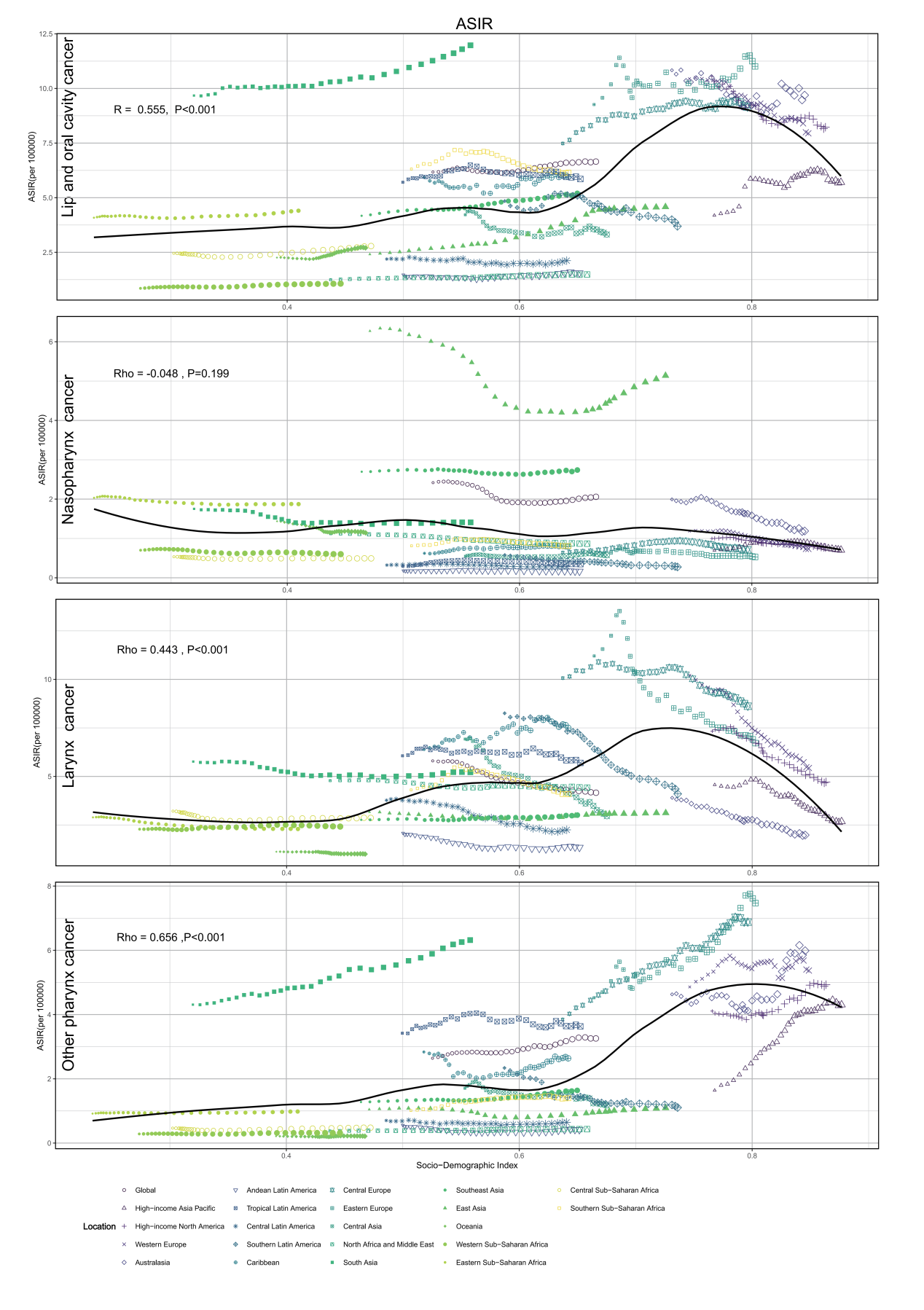
**

**Figure S6**

**

**

**Figure S7**

**
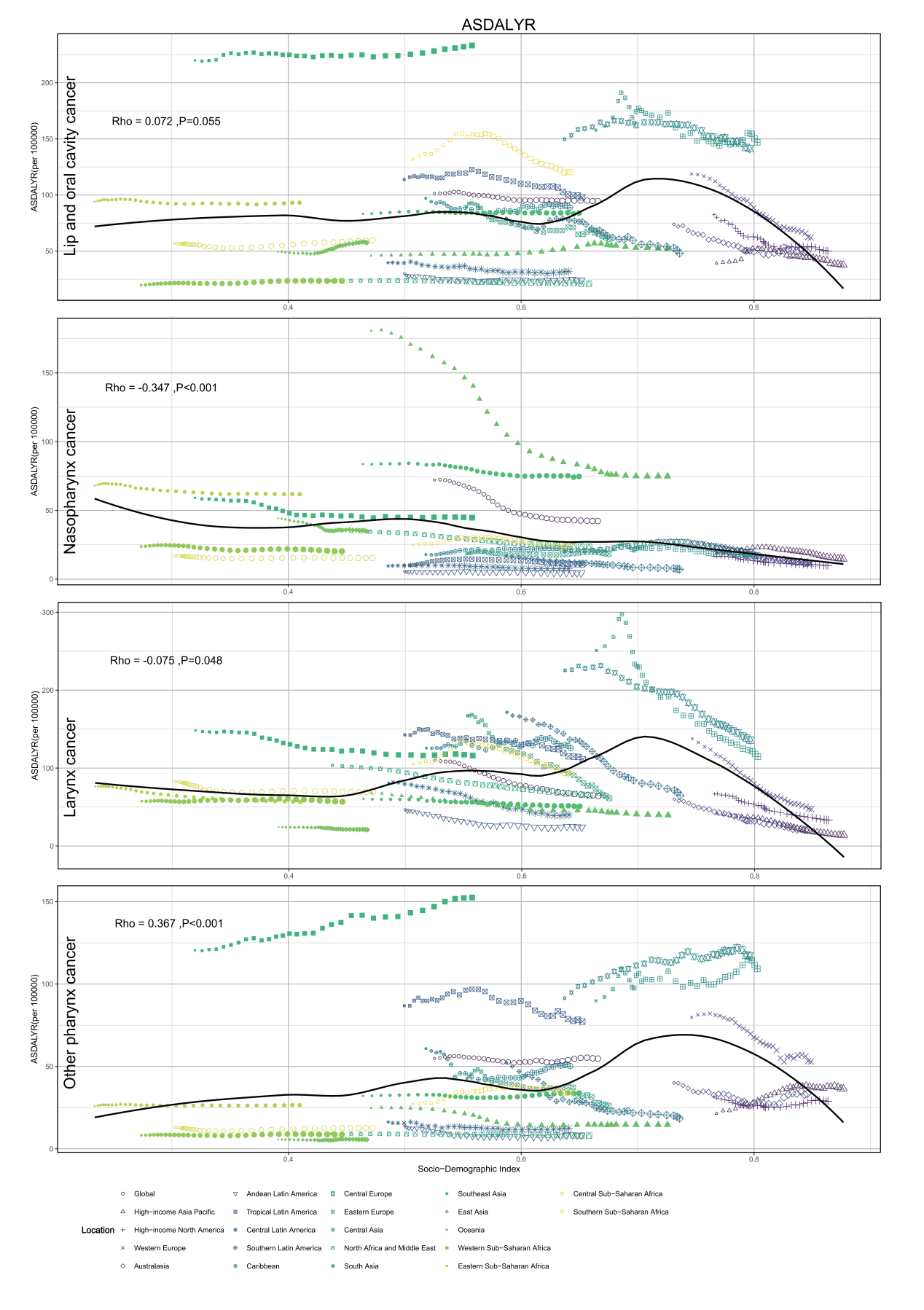
**

**Figure S8**


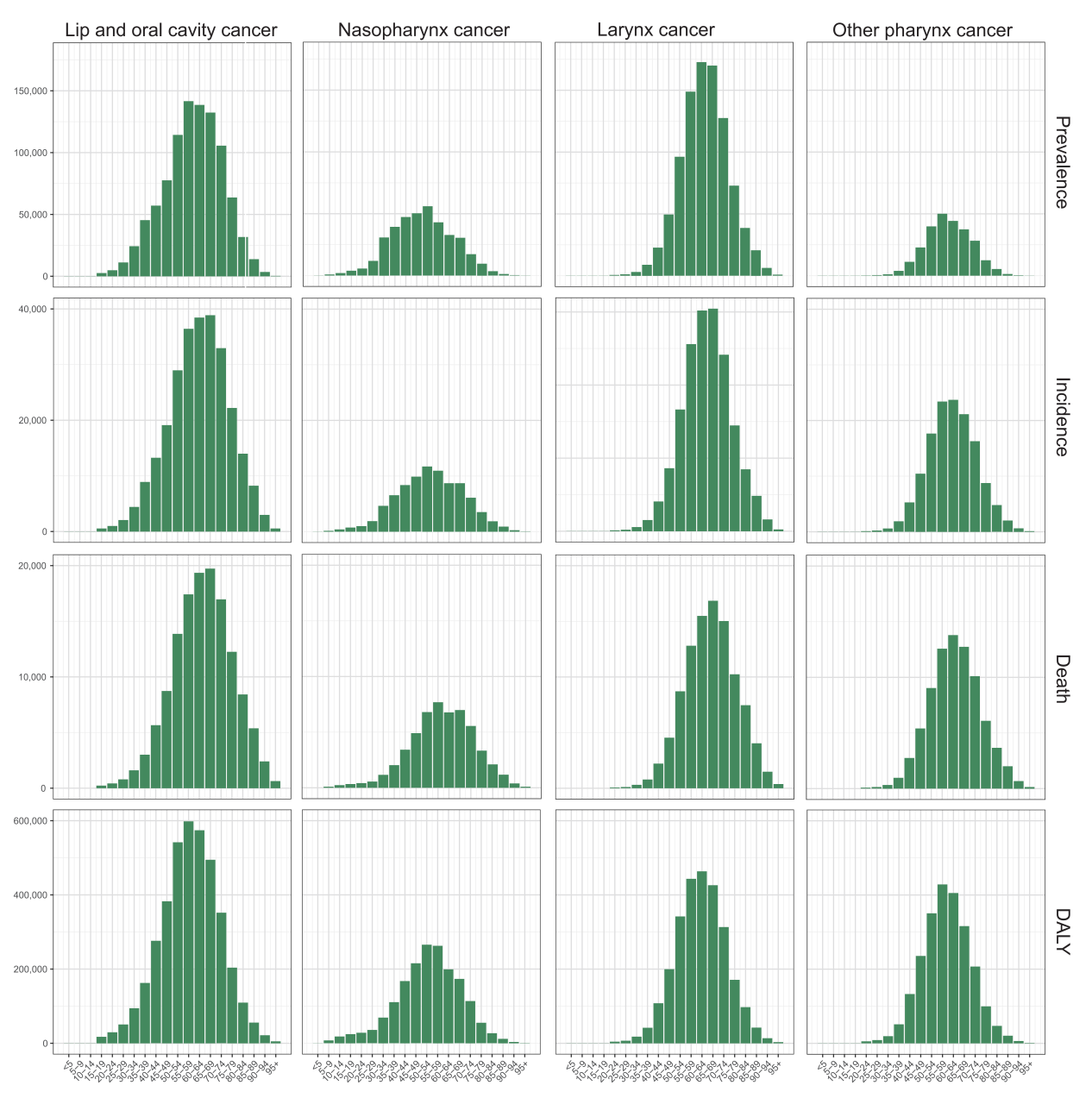


**Figure S9**


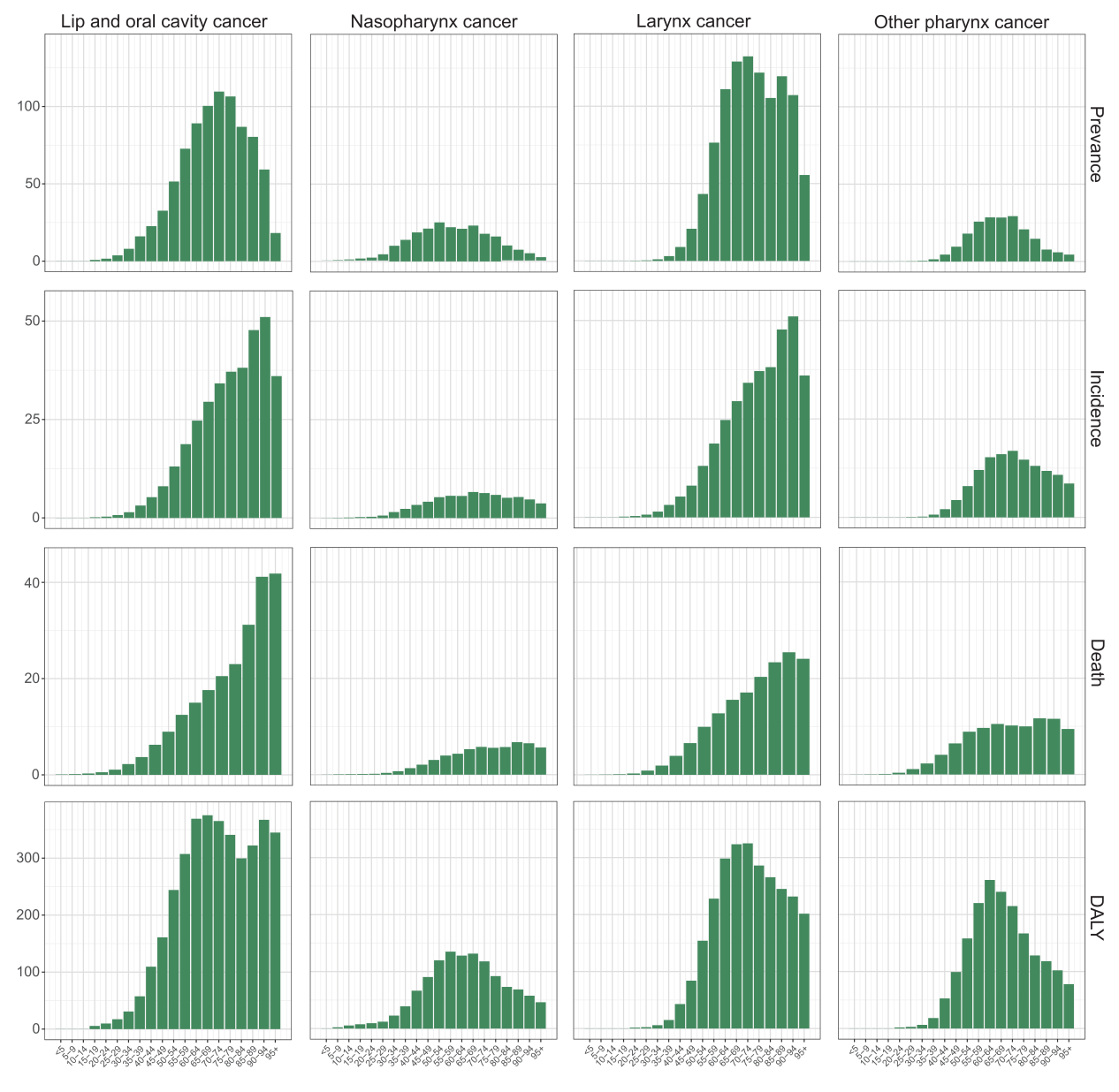


**Figure S10**

**
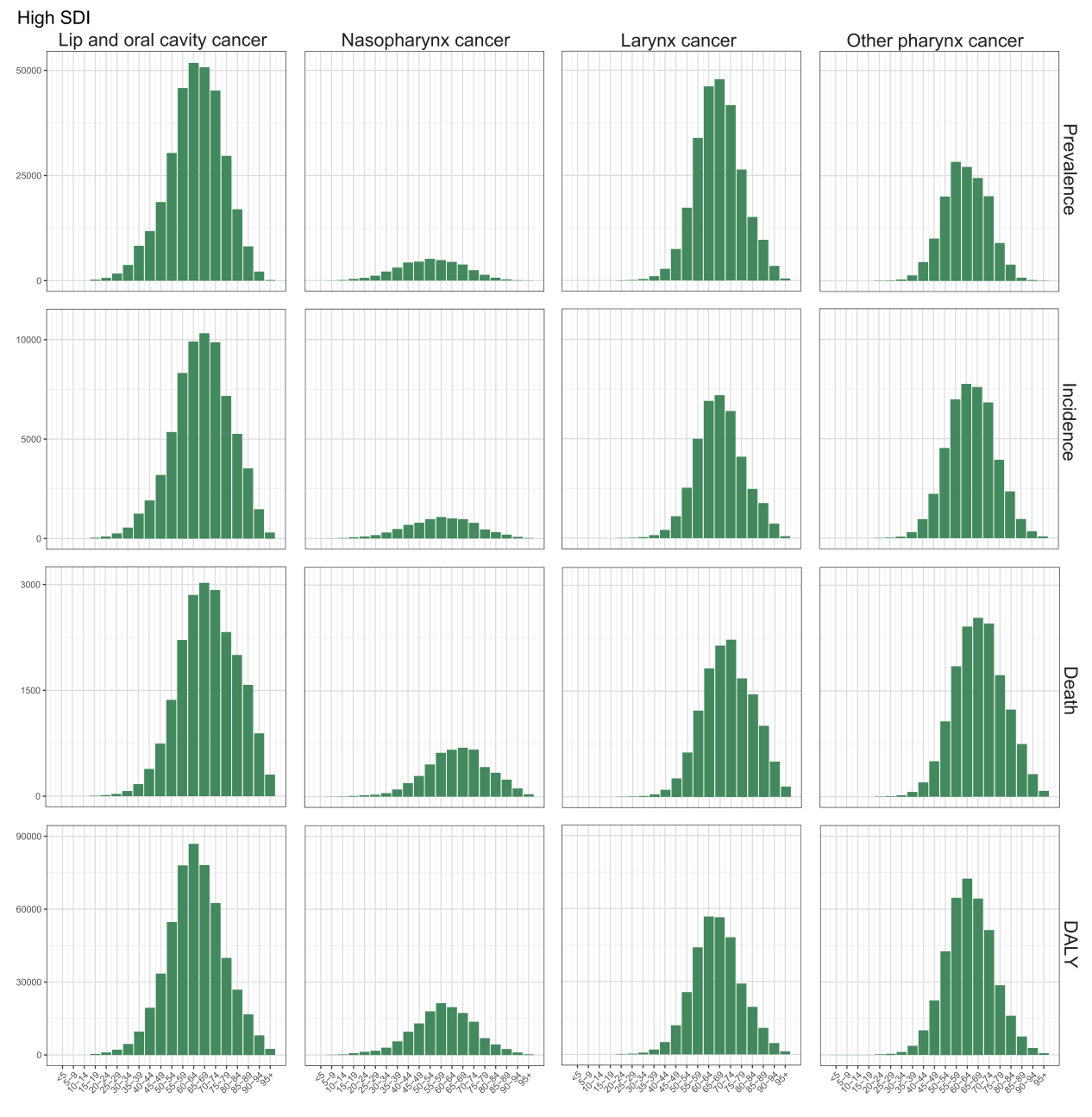
**

**Figure S11**

**
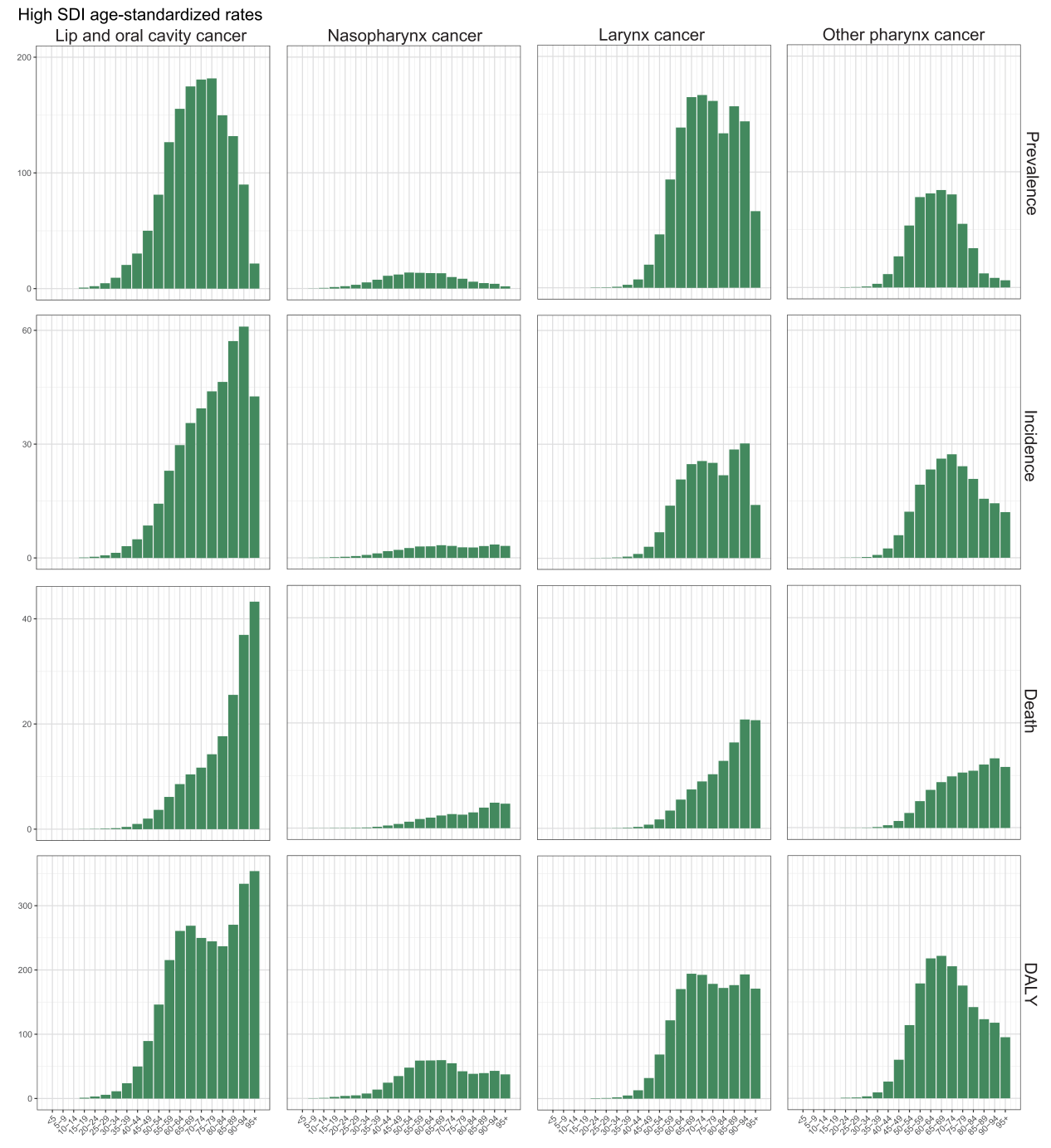
**

**Figure S12**

**
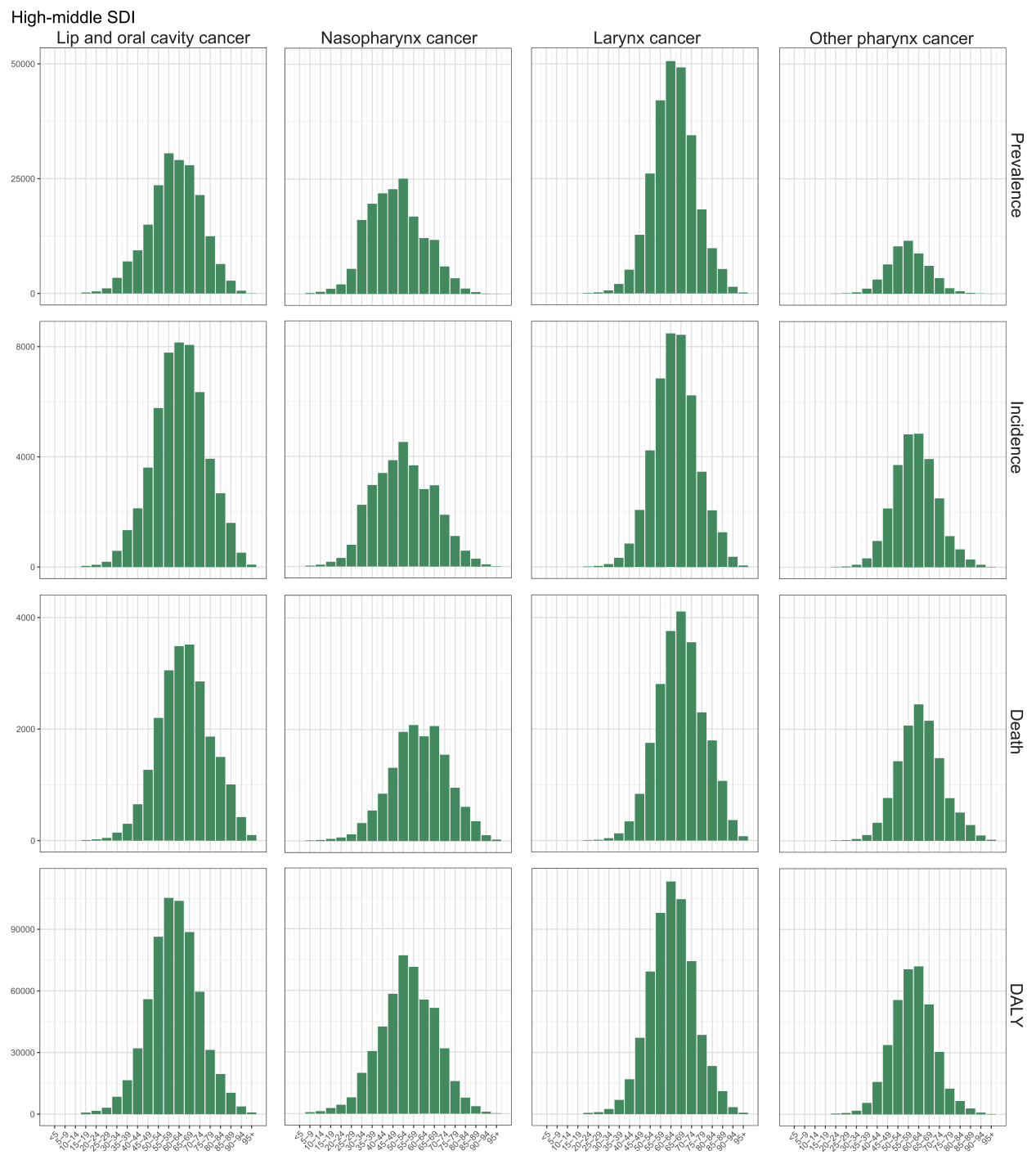
**

**Figure S13**

**
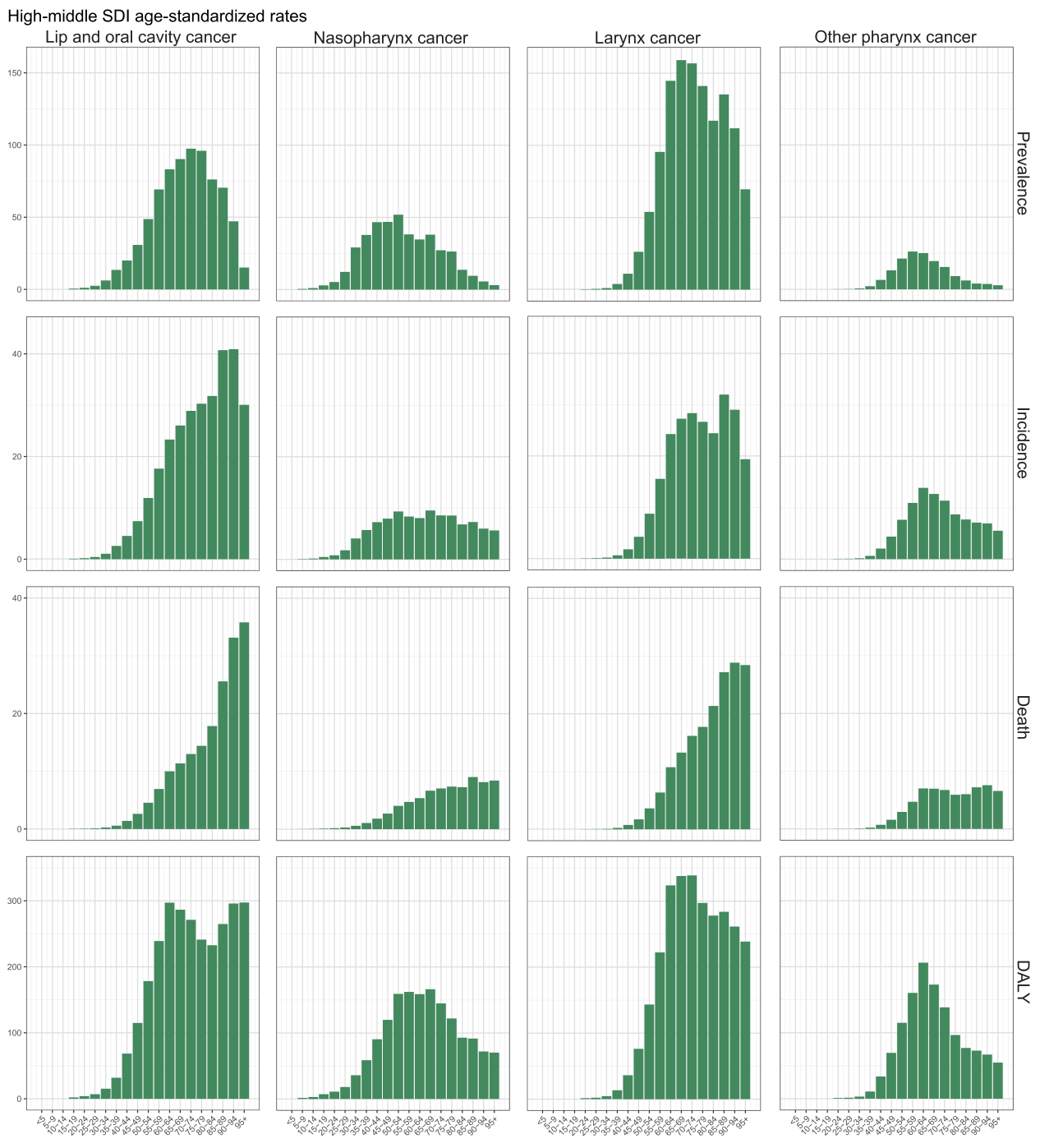
**

**Figure S14**

**
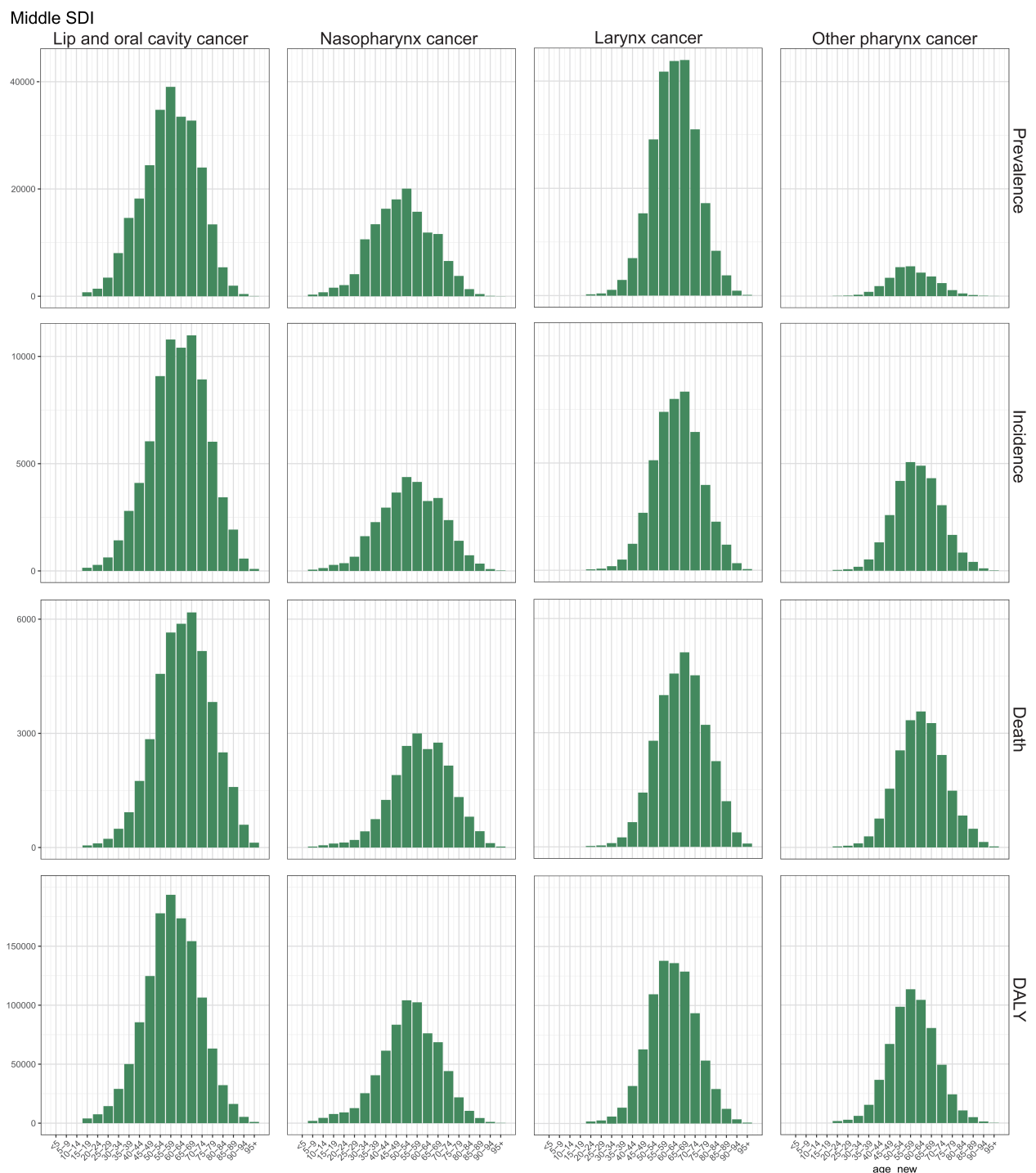
**

**Figure S15**

**
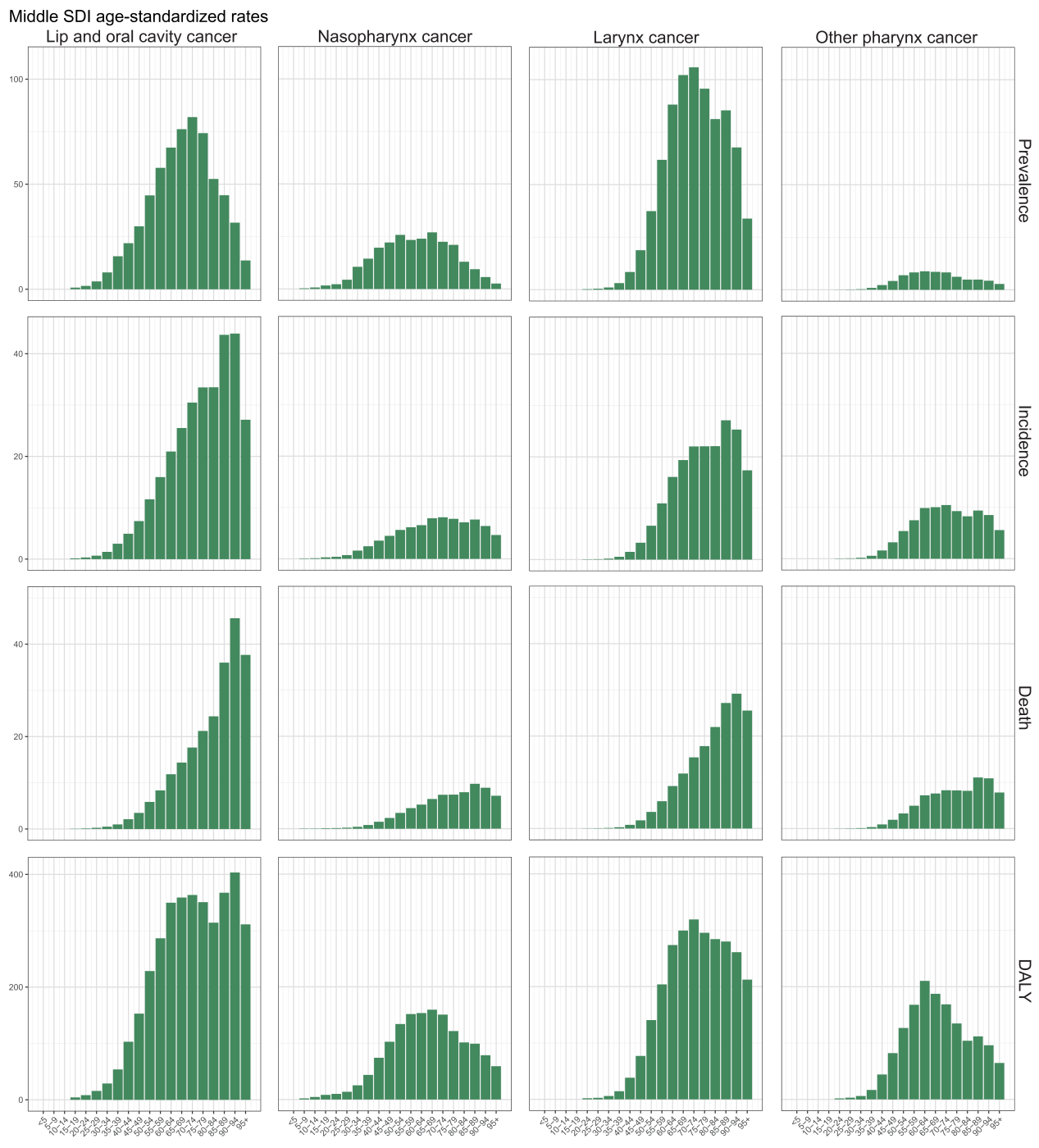
**

**Figure S16**

**
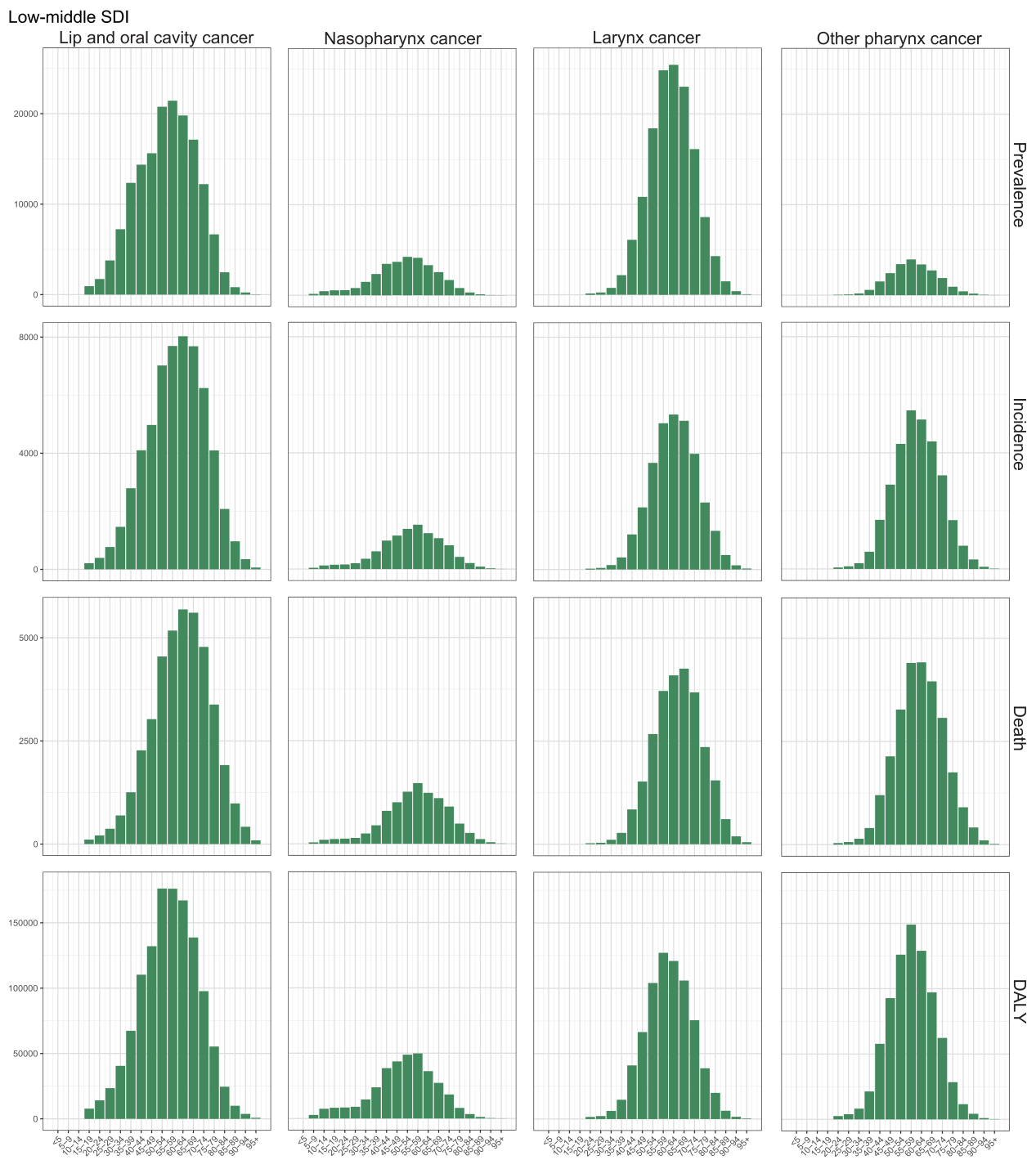
**

**Figure S17**

**
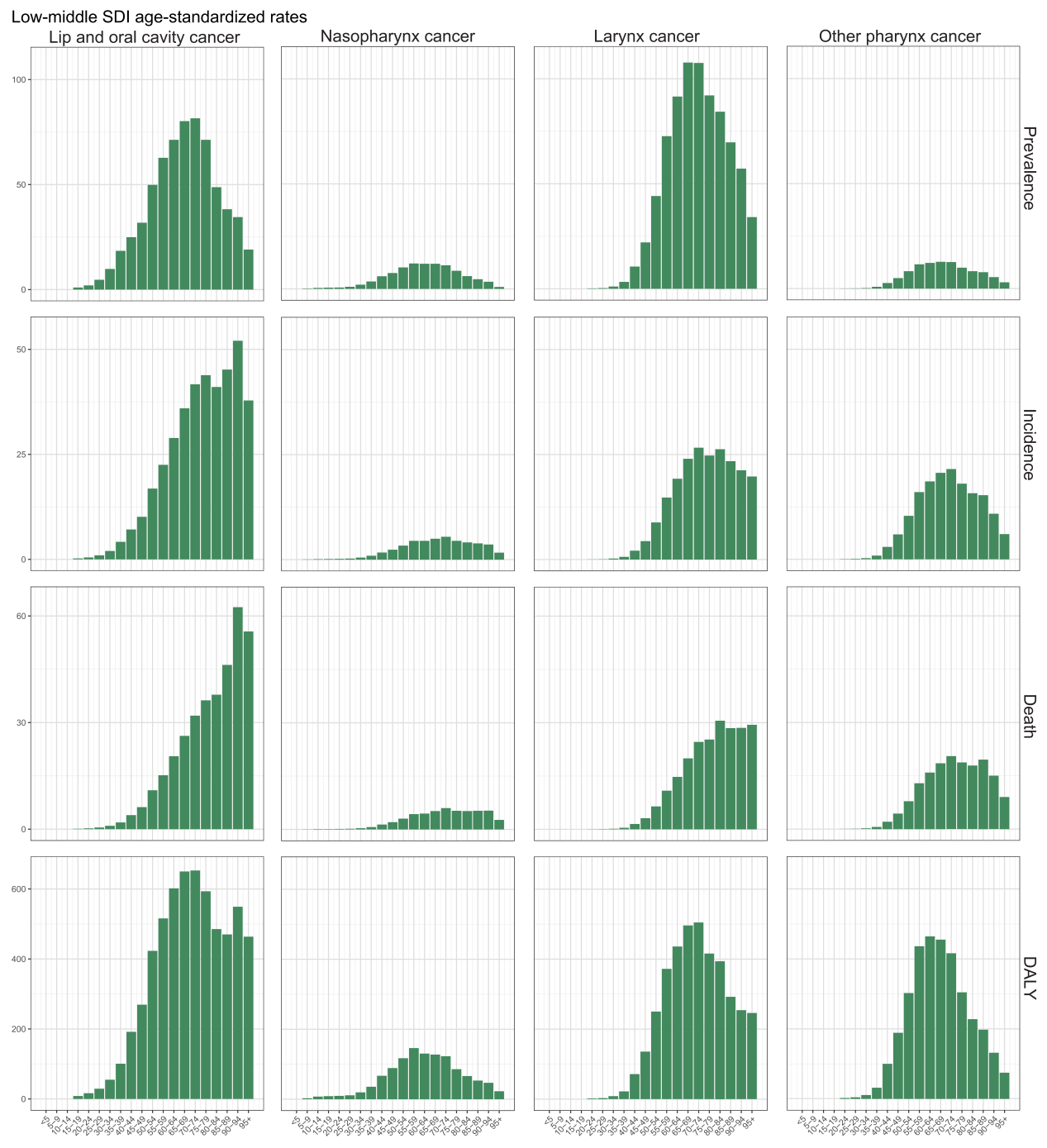
**

**Figure S18**

**
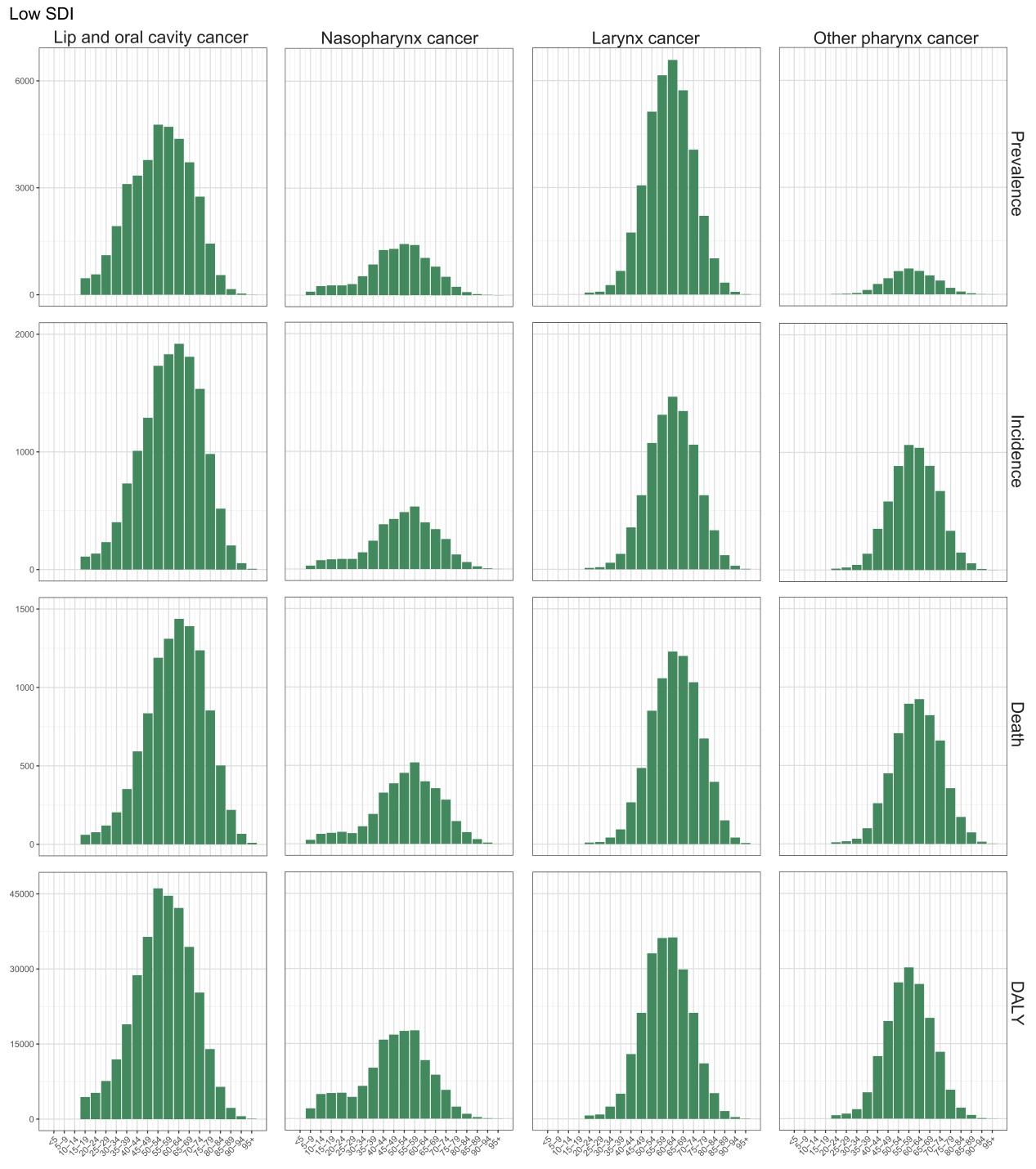
**

**Figure S19**

**
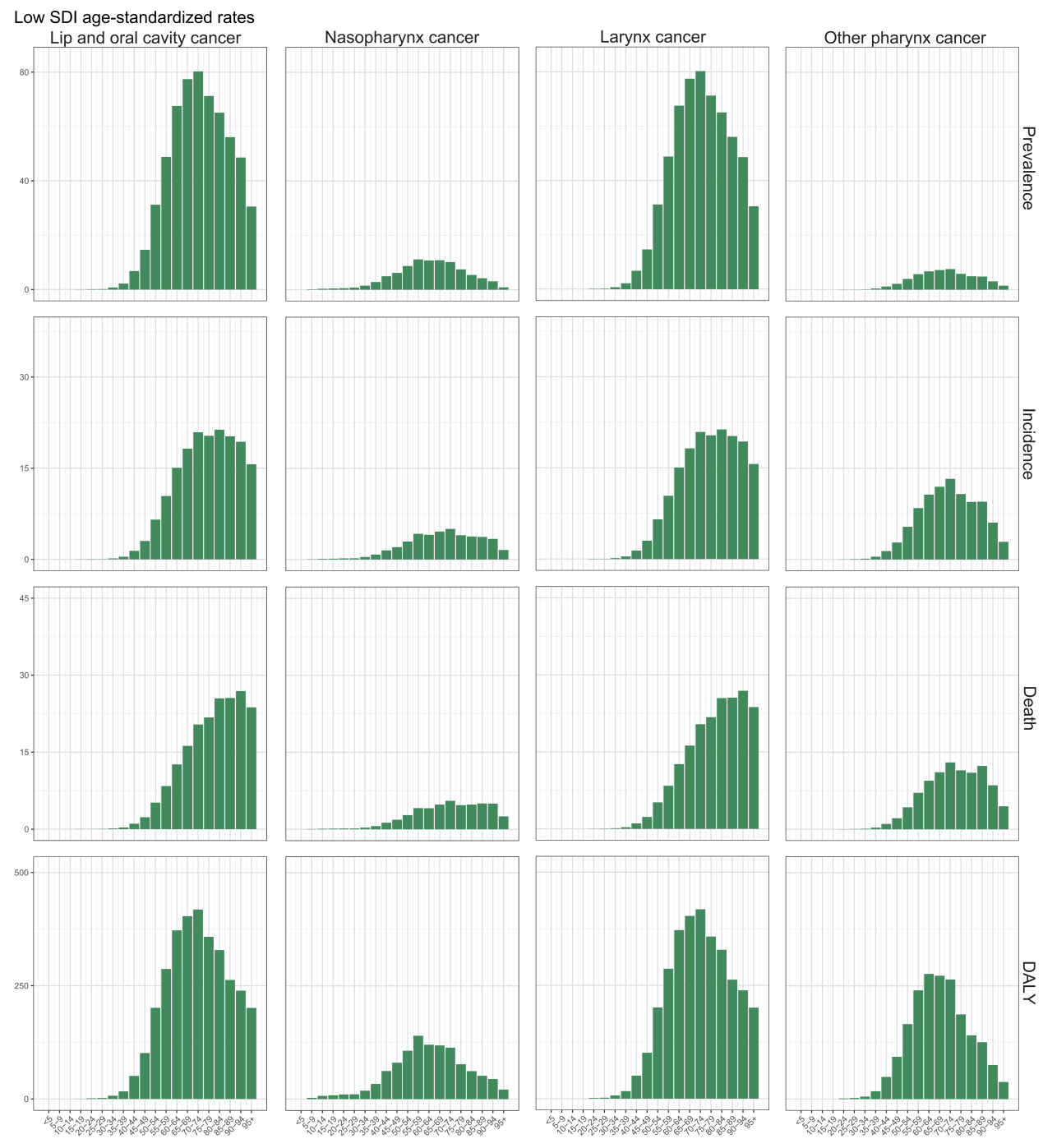
**

**Figure S20**


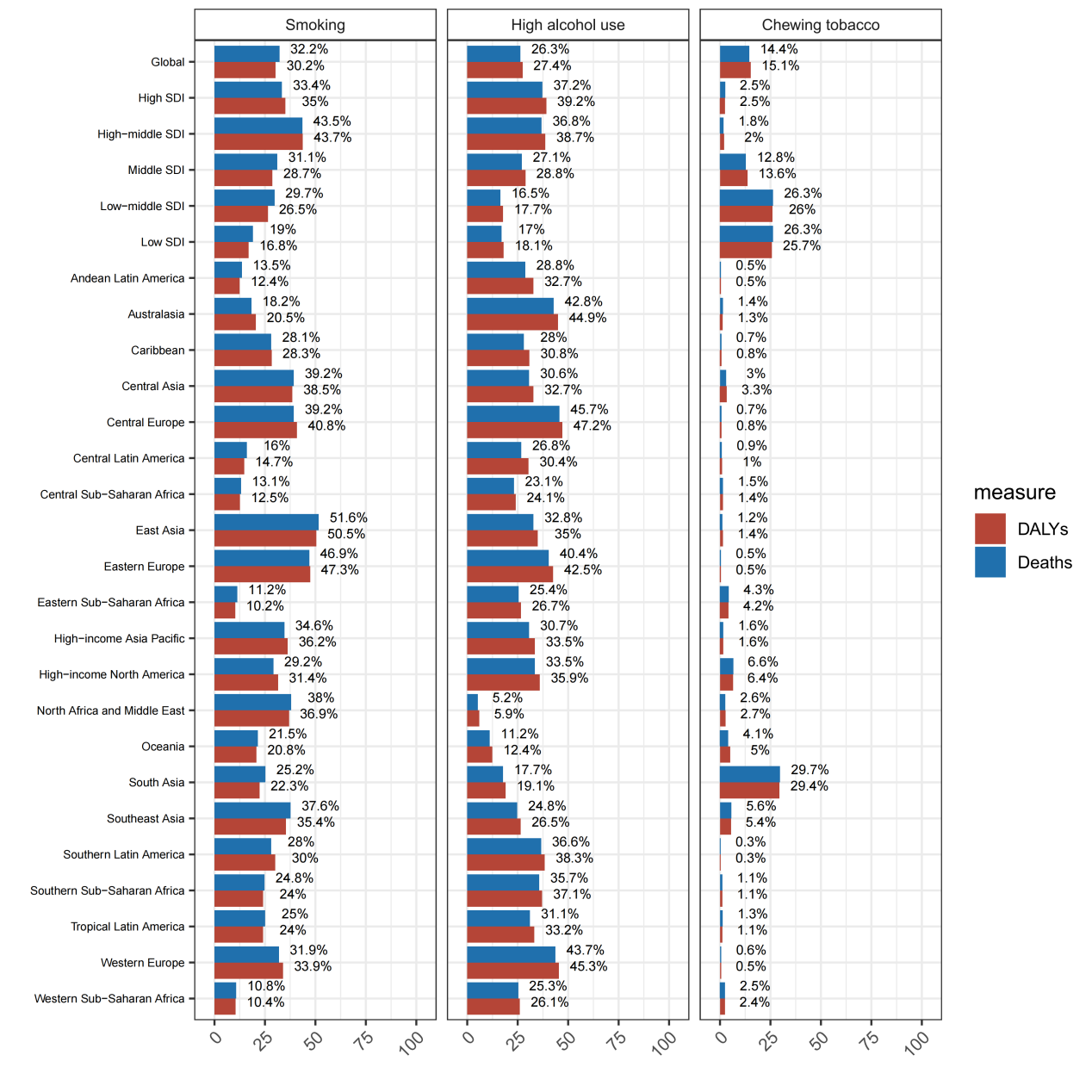


**Figure S21**

**
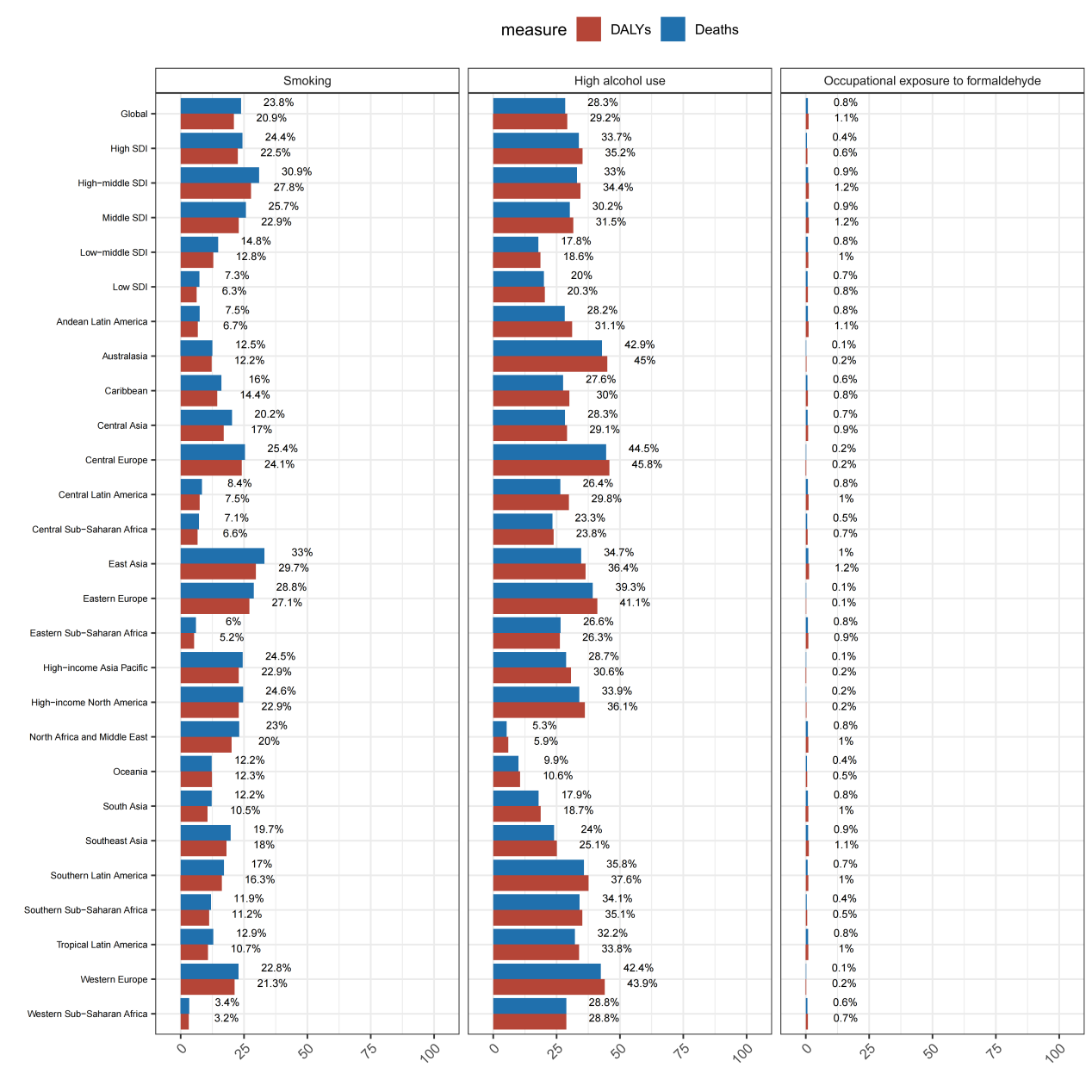
**

**Figure S22**

**
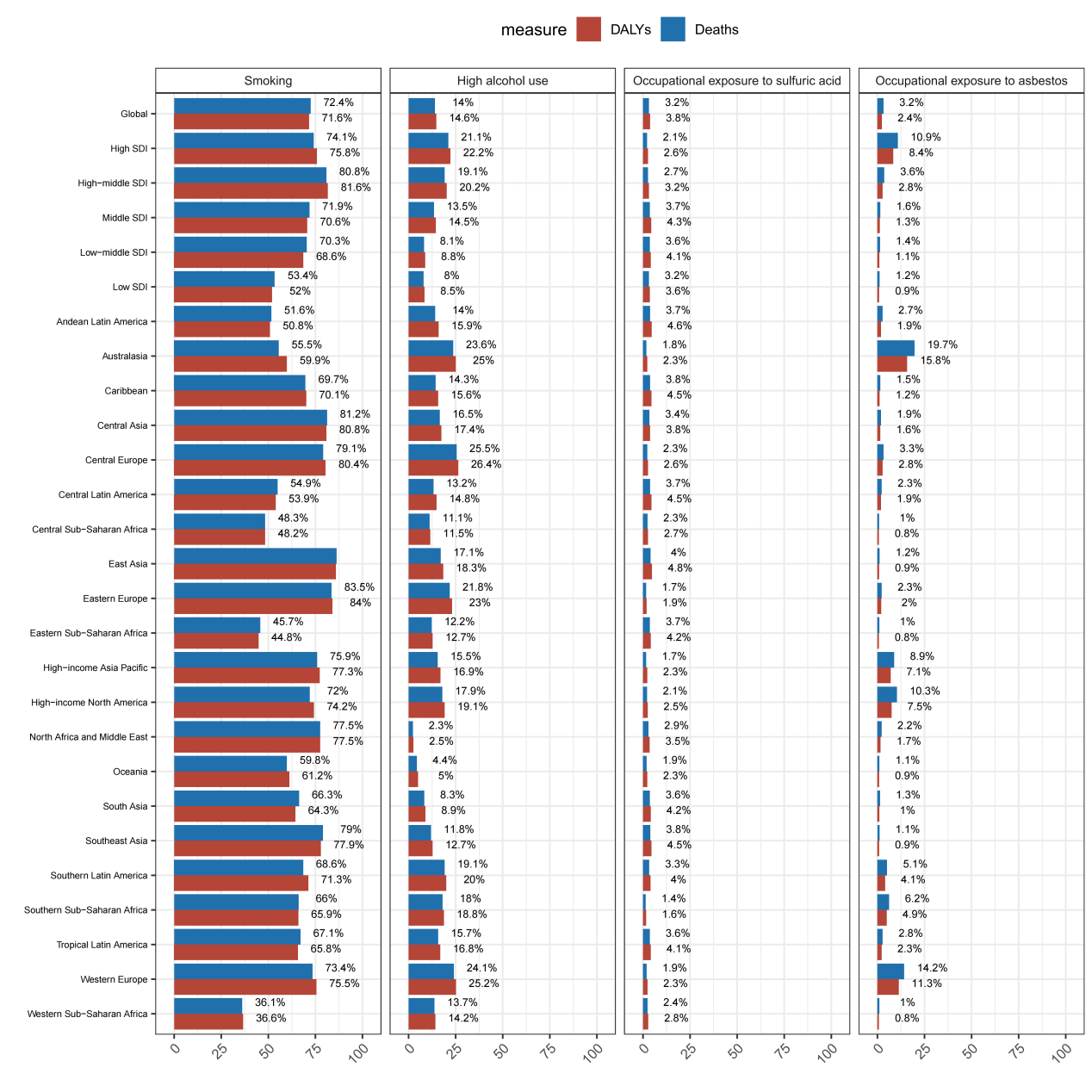
**

**Figure S23**

**
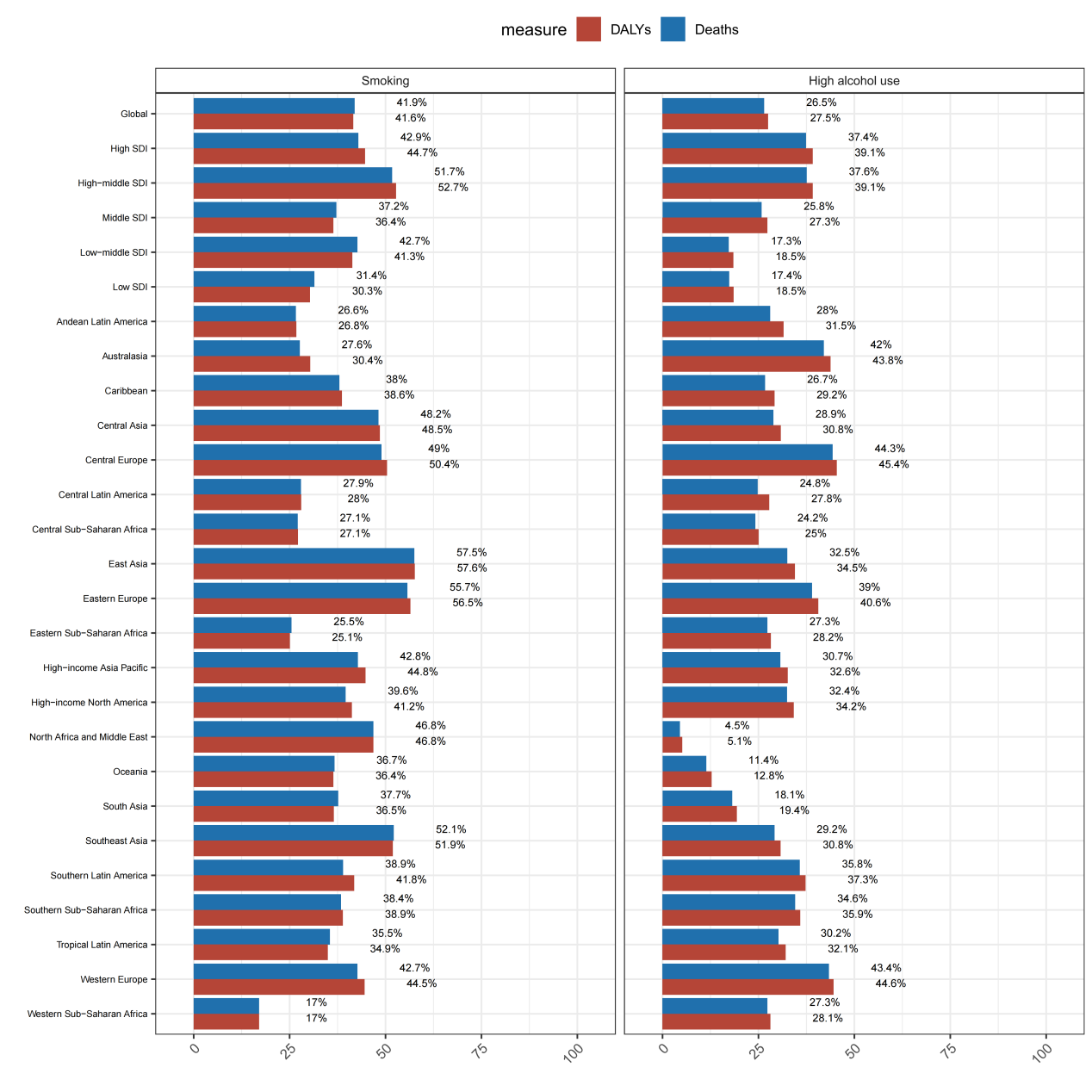
**
